# Effects of health risk assessment and counselling on physical activity in older people: A pragmatic randomised trial

**DOI:** 10.1371/journal.pone.0181371

**Published:** 2017-07-20

**Authors:** Anna Marie Herghelegiu, André Moser, Gabriel Ioan Prada, Stephan Born, Matthias Wilhelm, Andreas E. Stuck

**Affiliations:** 1 National Institute of Gerontology and Geriatrics “Ana Aslan”, Bucharest, Romania; 2 University of Medicine and Pharmacy “Carol Davila”, Geriatrics and Gerontology Department, Bucharest, Romania; 3 Department of Geriatrics, Inselspital, University Hospital, and University of Bern, Bern, Switzerland; 4 Institute of Social and Preventive Medicine, University of Bern, Bern, Switzerland; 5 Department of Cardiology, Interdisciplinary Center for Sports Medicine, University Hospital Bern, and University of Bern, Bern, Switzerland; Weill Cornell Medical College in Qatar, QATAR

## Abstract

**Background:**

Interventions to increase physical activity (PA) among older community-dwelling adults may be enhanced by using multidimensional health risk assessment (HRA) as a basis for PA counselling.

**Methods:**

The study was conducted among nondisabled but mostly frail persons 65 years of age and older at an ambulatory geriatric clinic in Bucharest, Romania. From May to July 2014, 200 participants were randomly allocated to intervention and control groups. Intervention group participants completed an initial HRA questionnaire and then had monthly counselling sessions with a geriatrician over a period of six months that were aimed at increasing low or maintaining higher PA. Counselling also addressed the older persons’ concomitant health risks and problems. The primary outcome was PA at six months (November 2014 to February 2015) evaluated with the International Physical Activity Questionnaire.

**Results:**

At baseline, PA levels were similar in intervention and control groups (median 1089.0, and 1053.0 MET [metabolic equivalent of task] minutes per week, interquartile ranges 606.0–1401.7, and 544.5–1512.7 MET minutes per week, respectively). Persons in the intervention group had an average of 11.2 concomitant health problems and risks (e.g., pain, depressive mood, hypertension). At six months, PA increased in the intervention group by a median of 180.0 MET minutes per week (95% confidence interval (CI) 43.4–316.6, p = 0.01) to 1248.8 MET minutes per week. In the control group, PA decreased by a median of 346.5 MET minutes per week (95% CI 178.4–514.6, p<0.001) to 693.0 MET minutes per week due to a seasonal effect, resulting in a difference of 420.0 MET minutes per week (95% CI 212.7–627.3, p< 0.001) between groups.

**Conclusion:**

The use of HRA to inform individualized PA counselling is a promising method for achieving improvements in PA, and ultimately health and longevity among large groups of community-dwelling older persons.

**Trial registration:**

International Standard Randomized Controlled Trial Number: ISRCTN11166046

## Introduction

Physical activity (PA) guidelines recommend a minimum of 75 vigorous or 150 moderate intensity minutes of PA per week for adults of all age groups, and PA beyond this amount for additional health benefit [1,2]. Adherence to this recommendation is poor, especially among older adults. According to the 2016 OECD report in European countries, approximately 36% of adults aged 16 years and older, and as many as 64% of adults aged 65 years and older do not meet this minimum PA level [3,4]. This epidemic of physical inactivity has detrimental health effects [5]. Physical inactivity is thought to determine 9% of premature mortality and its elimination would remove 6–10% of major noncommunicable diseases such as coronary heart disease, type 2 diabetes mellitus, and breast and colon cancers [6]. Among older adults, PA has shown additional benefits that include the prevention or delay of functional decline [7,8], protective effects on cognitive function [9], mood improvement in depressive disorders [10], reduced fear of falling and improved balance [11], and prevention of fractures by increasing muscle strength, balance, and bone mineral density [12]. Not only is the minimal recommended level of PA beneficial for health, but studies have shown a favourable dose-response relationship of further increases of PA up to 3 to 5 times the minimum recommended level [13].

So, why do older persons not engage in more PA? Evidence for the favourable effects of PA on health is abundant, and most older persons give high priority to staying healthy in old age. Many are aware of general health benefits of PA but believe that high levels of PA may be unnecessary or perhaps even harmful for them personally [14,15]. More importantly, several studies have shown that many older persons remain at low levels of PA due to problems or concerns related to their individual health status, such as pain or discomfort with PA, physical limitation, fear of falling, or presence of one or multiple chronic conditions [14–16]. Not surprisingly, multiple intervention studies have found only modest effects of interventions to increase PA on older persons’ PA levels [16].

We therefore designed a novel intervention for increasing PA and reducing sedentary behaviour in older persons. To address potential barriers to PA relevant in old age, we combined PA counselling with an initial multidimensional health assessment to detect concomitant health problems and risks that may negatively influence older persons’ levels of PA. The intervention began with a tool of health risk assessment (HRA) that has been validated for measuring social, functional, somatic, psychological, and environmental factors in community-dwelling older persons [17–22]. We based subsequent PA counselling on behavioural change techniques and arranged monthly counselling sessions over a period of six months that aimed at raising study participants’ PA awareness, and developing, implementing, and possibly modifying individual PA related goals while addressing potential PA barriers [23–26]. We hypothesized that this intervention would substantially improve older persons’ PA levels. A secondary study question explored whether effects differ for different types of PA recommendations and between subgroups of older persons.

## Methods

### Study organisation

The Ethics Committee of the National Institute of Gerontology and Geriatrics “Ana Alan” approved the study (972/2014). Participants were recruited from patients referred to the “Ana Aslan” outpatient geriatric clinic by their general practitioners and gave written informed consent. The RAHEO (Medical Risk Assessment and Health Education in Older People) trial is a randomised controlled trial on HRA and specialist geriatric counselling in older persons conducted in Bucharest, Romania. The ethics committee approved the study protocol January 28, 2014 (S1 and S2 Text). Based on this protocol, the trial was registered on June 17, 2014. Due to a management error, the study was registered shortly after patient enrolment started (first patient enrolled May 12, 2014). According to the original study protocol submitted to the ethics committee and to trial registration, the trial was planned for an ambulatory and a hospital-based setting. However the study in the hospital setting was terminated in August 2014 because the required sample size was not reached within four months after the start of the study. The study in the ambulatory setting was carried out as planned with no deviations from the study protocol. The RAHEO trial was conducted by the geriatrics office of the Ambulatory Clinic of the National Institute of Gerontology and Geriatrics “Ana Aslan”, Bucharest, Romania. The overall trial was designed to address effects of HRA-based counselling in the following domains: physical activity, nutrition, psychosocial factors, and preventive care. This is the first published report of the RAHEO trial.

### Participants and randomisation

Participants were recruited from patients referred to the “Ana Aslan” outpatient geriatric clinic by their general practitioners. From May 12 to July 7 2014, all patients aged 65 years and older were consecutively evaluated for eligibility by trained researchers at the study centre in Bucharest. Patients who met any of the following criteria were excluded: moderate to severe dementia (equivalent to a Mini-Mental State Examination score < 20) [27], severe disability (need of human help in one or more basic activities of daily living) [28], terminal illness, major surgery within the last three months, not living in catchment area (more than 2–4 hours travel time), living in nursing home, not speaking the Romanian language, inability or unwillingness to complete the prerandomisation questionnaire, and not wishing to give written informed consent. Recruitment was continued until the required sample size of 200 study participants was reached.

The independent study centre in Bern, Switzerland randomly divided the consenting participants equally between intervention and control groups using a computer generated random allocation sequence. Persons allocated to the intervention group were invited to monthly counselling sessions over a six-month follow-up period at the geriatrics clinic, with the first session taking place four to eight working days after randomisation. Participants in the control group were invited to a follow-up consultation session six months after randomisation. Patients of both intervention and control groups continued to receive usual care by their primary care physicians.

### Data collection

The Romanian and English versions of the questionnaires used in this trial are included in the study protocol (S2 Text). Baseline data were obtained prior to randomisation, and consisted of a brief face-to-face interview with the participant by a trained interviewer and the HRA for Older Persons questionnaire. In the interview trained researchers collected information on participant income (pension slip), body weight and height, blood pressure, and medication. The HRA for Older Persons questionnaire is a multidimensional self-administered questionnaire composed of validated questions identifying potential health and disability risk factors in relevant domains among older persons [29]. Its development relied on an extensive literature review and focus group work, and it has shown high acceptance among older persons and primary care physicians in various European countries [29]. A Romanian version of the questionnaire was developed based on a scientific update, cultural adaptation, backward translation, and coordination with existing Romanian guidelines. Participants completed this questionnaire on site. The completed forms were scanned and sent to the independent study centre for double data entry.

Baseline information on PA was obtained from participants’ answers to a brief version of the International Physical Activity Questionnaire (IPAQ) that was part of the HRA for Older Persons questionnaire [30]. The IPAQ enquires about frequency and time spent doing different types of PA lasting at least 10 minutes at one of three different levels of PA (vigorous activity such as jogging, cycling at high speed; moderately vigorous activity such as brisk walking, swimming, dancing, cycling at normal speed; and moderate activity with time spent walking at a normal pace). Each set of questions is introduced by a brief instruction while the types of physical activities are exemplified with pictures. In addition, the IPAQ asks about usual sitting time per day, and we added a question on the intention to increase one’s level of PA based on the transtheoretical model of behaviour change [26]. Appraisal was based on the participants’ own assessments and recollection of their activities during the previous week. Collection of six-month follow-up data took place between November 26, 2014 and February 3, 2015. Participants from intervention and control groups were invited for a final on-site visit at the study centre and were asked by research assistants who were blinded for participants’ treatment allocation status to complete a shortened version of the self-administered HRA for Older Persons questionnaire including the IPAQ.

### Outcome measures

The primary outcome was derived from analyses of the participants’ answers to the IPAQ questionnaires at six months according to the IPAQ scoring standards [31], and defined as the metabolic equivalent of task (MET, multiples of the resting metabolic rate) minutes per week; one MET minute corresponds to 1 kilocalorie in an adult who weighs 60 kg. Secondary outcomes were (1) vigorous activity (measured as the proportion of persons who reported at least 10 minutes of vigorous activity at least once during the preceding week), (2) moderately vigorous or vigorous activity (similarly, measured as the proportion of persons who reported such an activity at least once during the preceding week), (3) walking (measured as number of minutes walking per week), and (4) physical inactivity (measured as hours sitting per day).

### Intervention

All sessions took place at the outpatient geriatric clinic in Bucharest and consisted of face-to-face health counselling meetings of the participant with an experienced geriatrician lasting 15 to 30 minutes. The health counsellor relied upon the individualized computer-generated health profile report derived from each participant’s answers to the HRA for Older Persons questionnaire. This report covered PA and other health domains relevant for PA counselling (including pain, cardiovascular risk factors, nutrition, social network, and preventive care).

At the first counselling session, the geriatrician counsellor asked the participant about additional details of previous PA, his or her motivation to increase PA, and potential problems related to engaging in PA such as pain, fear of falls, unsafe neighbourhood for outdoor activity, and depressive symptoms. The counsellor addressed barriers to performing PA, and options such as pain management, referral for additional work-up, and social group activities to overcome them and explored options for increasing the current type of PA or starting new kinds of PA. Examples of PA were discussed, along with resistance training exercises [32]. The counsellor explained benefits and risks of increasing PA with each participant, taking into account findings from the participant’s health profile report, such as cardiovascular risk factors, pain, and emotional function. Finally, the counsellor and participant agreed upon an action plan with realistic, goal-oriented PA recommendations. The plan could include an increase in PA, or alternatively, maintenance of a high level of PA on a regular daily basis. Subsequently, the counsellor and the participant discussed the need for action in other domains such as nutrition and preventive care. At the end of the first counselling session, the participant was given the computer-generated individualised health profile report and a written summary with the individualised recommendations.

During the monthly follow-up sessions, participants were asked how successful they had been in reaching their goals for PA, and about reasons that may have prevented them from following their PA programmes. If a goal was achieved, the counsellor offered praise and reinforced motivation by adding new suggestions and diversifying the daily exercise schedule. If a goal was not achieved, the participant’s knowledge and motivation were re-evaluated while difficulties in achieving recommended PA were identified. When new problems were identified, recommendations were adapted as needed. At the end of each follow-up session, participants received a brief written note with a summary of the updated recommendations as well as the date and time for the next appointment. To improve participants’ adherence to recommendations, counselling sessions took place in a calm, assuring, and friendly manner, and participants’ full attention was regularly confirmed during discussions to support self-management of recommended actions by participants. The final counselling session included additional reinforcement to sustain implementation of recommendations.

To facilitate comparisons of the findings of our study with those of others, S1 Table summarizes the study design based on the Behaviour Change Technique Taxonomy v1 [24]. According to this classification, our study’s intervention is based on ten out of 93 possible behaviour change techniques. S1 Table presents examples illustrating how we implemented each of these ten techniques. Information from the HRA for Older Persons questionnaire was relevant for all elements of the intervention, and the counselling steps were based on the principles of the transtheoretical model of behaviour change [26].

### Statistical analysis

The estimated sample size to detect a 20% increase in the proportion of physically active persons was 200, based on an expected drop-out rate of 10%, a two-sided alpha of 0.05, a power of 0.8, and a control group PA prevalence of 60% (based on a power analysis for two-sample proportions test). All analyses were conducted according to a detailed analysis plan (S3 Text). Group differences were tested by the chi-square test for binary variables, and by the Kruskal-Wallis test for continuous variables. For binary outcome variables we report odds ratios (OR) with 95% confidence intervals (CI) from logistic regression models as effect measures. For continuous variables we report median group differences from quantile regression models with 95% CI [33]. Median values of primary outcomes were calculated with an interquartile range (IQR, range between first quartile and third quartile). All p-values are two-sided and p<0.05 was considered statistically significant. Possible selection bias was assessed by an inverse probability of censoring weighting approach [34,35]. We used age, gender, and MET minutes per week as predictors for censoring in the inverse probability of censoring weighting analysis. An exploratory subgroup analysis was performed to investigate effects of intervention on specific subgroups of the study population. All statistical analyses were performed in R V.3.1.1 (R Project, University of Vienna, Austria). Graphics were done with the package ggplot2 [36].

## Results

**Fig 1:**
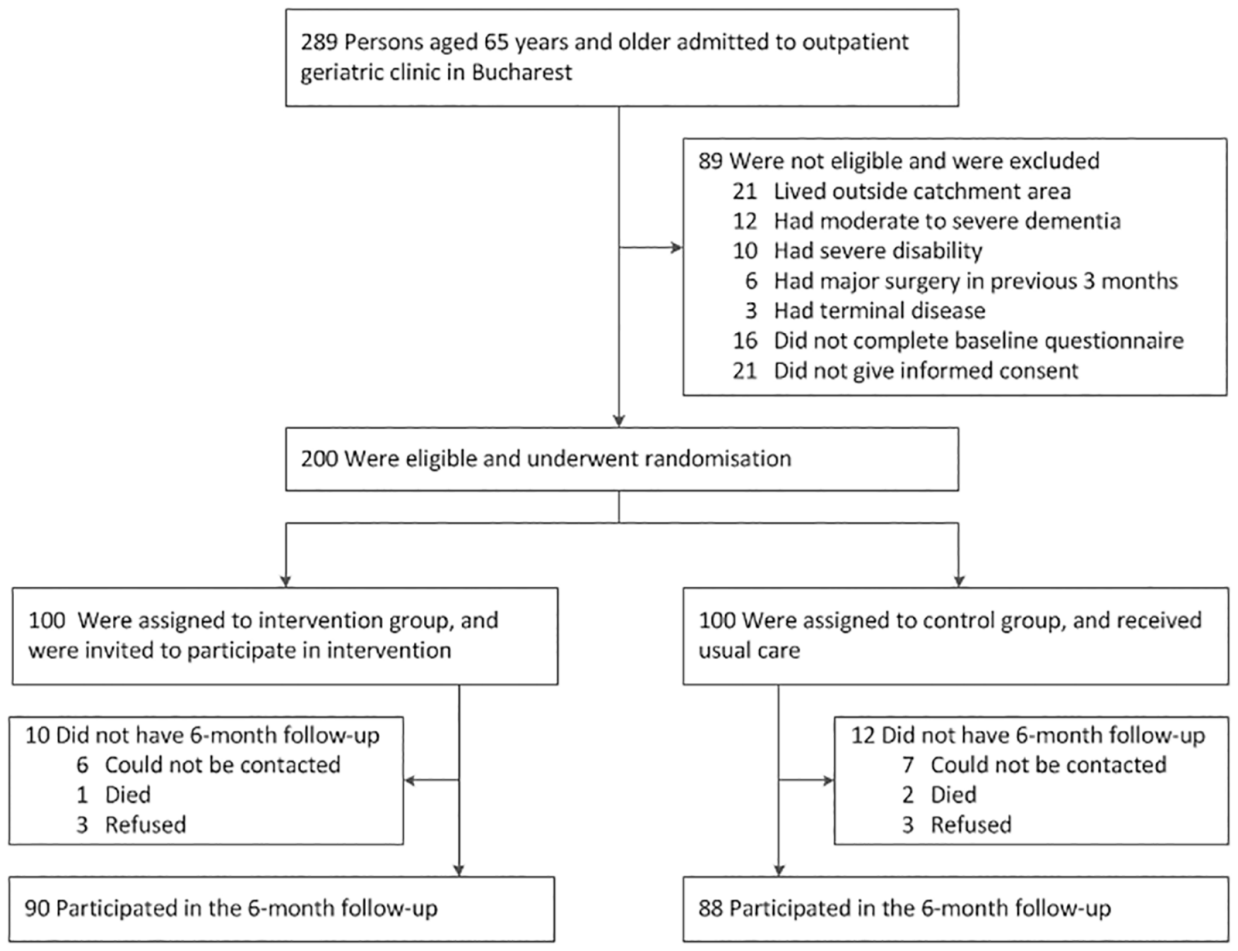
Study CONSORT flow diagram.

From the 289 persons who were assessed for eligibility, 200 were included in the two study groups, with 100 persons allocated to the intervention group, and 100 persons to the control group (for CONSORT flow diagram, see Fig 1).

Participants were predominantly female, had a relatively high level of education, were mostly independent, but often complained about poor health and pain (Table 1). At baseline, about 80% of participants engaged in the minimum recommended level of PA of 450 MET minutes or more, with many persons reporting high numbers for walking, but only low numbers for vigorous types of activities. Intervention and control groups did not differ in any of the baseline characteristics (Table 1).

**Table 1:** Study group characteristics at baseline.

Characteristic	Intervention group *n* = 100	Control group *n* = 100
**Age at randomisation (years) ─ median (IQR)**	74.8 (71.0–81.0)	75.0 (69.8–80.0)
**Women ─ n (%)**	77 (77.0%)	72 (72.0%)
**Income < 848 RON**^a^ **─ n (%)**	25 (25.0%)	19 (19.0%)
**Education: high school or more ─ n (%)**	93 (93.0%)	92 (92.0%)
**Living alone ─ n (%)**	7 (7.0%)	9 (9.0%)
**Number of chronic conditions ─ median (IQR)**	6.0 (5.0–7.0)	6.0 (5.0–7.0)
**Fair or poor self-perceived health ─ n (%)**	65 (65.0%)	57 (57.0%)
**Presence of moderate to severe pain ─ n (%)**	95 (95.0%)	98 (98.0%)
**Limitation (need for help) in instrumental ADL ─ n (%)**	14 (14.0%)	10 (10.0%)
**PA: MET minutes per week ─ median (IQR)**	1089.0 (606.0–1401.7)	1053.0 (544.5–1512.7)
**PA: ≥ 450 MET minutes per week ─ n (%)**	80 (80.0%)	78 (78.0%)
**PA: ≥ 900 MET minutes per week ─ n (%)**	57 (57.0%)	55 (55.0%)
**Vigorous PA at least once per week ─ n (%)**	2 (2.0%)	10 (10.0%)
**Moderately vigorous or vigorous PA at least once per week ─ n (%)**	42 (42.0%)	47 (47.0%)
**PA: Minutes of walking per week ─ median (IQR)**	210 (140.0–330.0)	210 (105.0–330.0)
**PA: Sitting ≥4 hours per day ─ n (%)**	77 (77.0%)	77 (77.0%)

ADL, activities of daily living; IQR interquartile range; MET, metabolic equivalent of task; PA, physical activity; for definition of variables see Methods section.

^a^848 RON is the 2014 average pension rate for Romania [37].

The baseline questionnaire also enquired about intention to change PA behaviour. Among those with no intention to increase their PA level (63%), the main reported reason was “I am already active”, an answer that could reflect either a lack of information about optimal PA levels or difficulty in self-assessing daily activities. Other main reasons for not planning to increase PA were increased pain during PA and the existence of limiting health conditions. The two groups did not differ in any of these factors (Table 2).

**Table 2:** Self-reported reasons for not increasing physical activity among the subgroup of participants with no intention to increase physical activity at baseline.

Self-reported reason	Intervention group *n* = 62	Control group *n* = 64
**Is already regularly active ─ n (%)**	27 (43.5%)	33 (51.6%)
**Experiences pain with physical activity ─ n (%)**	20 (32.3%)	8 (12.5%)
**Has illness limiting ability to be physically active ─ n (%)**	10 (16.1%)	17 (26.6%)
**Does not have anyone to do it with ─ n (%)**	5 (8.1%)	9 (14.1%)
**Does not know suitable activity opportunities ─ n (%)**	4 (6.4%)	2 (3.1%)
**Does not have time ─ n (%)**	0 (0%)	3 (4.7%)
**Weather ─ n (%)**	1 (1.6%)	1 (1.6%)
**Has a physical disability ─ n (%)**	1 (1.6%)	0 (0%)
**Costs ─ n (%)**	0 (0%)	0 (0%)

Data are *n* (percent). Percentages add to >100% because persons could report more than one reason.

Table 3 summarizes the prevalence of concomitant problems and risks identified in older persons in the intervention group. These findings are based on the participants’ answers to the baseline HRA for Older Persons questionnaire prior to the start of the intervention. Given the eligibility criteria for study participation, the study participants were neither demented nor disabled (i.e., had no need for human assistance for performing basic ADL). However, they were generally frail with an average of 11.2 concomitant health problems and risks (e.g., pain, limitation in instrumental ADL, depressive mood, hypertension).

**Table 3:** Prevalence of concomitant health-related risk factors in the elderly persons allocated to the intervention group (N = 100)^a^.

Risk factor domain	Description	Number (%)
**Activities of daily living**	Impaired basic ADL	26 (26.0)
	Impaired instrumental ADL	71 (71.0)
**Alcohol use**^a^	Possible hazardous alcohol use	17 (17.0)
**Chronic conditions**	High blood pressure	79 (79.0)
	High cholesterol	89 (89.0)
	Diabetes	13 (13.0)
	Heart disease	69 (69.0)
	Stroke	6 (6.0)
	Pulmonary disease	11 (11.0)
	Osteoporosis	53 (53.0)
	Rheumatism	79 (79.0)
**Falls**	Repeated falls	7 (7.0)
	Fear of falling	42 (42.0)
**Hearing**	Impaired hearing	64 (64.0)
**Incontinence**	Urinary incontinence	40 (40.0)
**Memory**	Memory problem	58 (58.0)
**Mood**	Possible depression	88 (88.0)
**Nutrition**	Underweight	0 (0.0)
	Overweight	28 (28.0)
**Pain**	Moderate or severe pain	95 (95.0)
**Social factors**	Low emotional support	50 (50.0)
	Risk for social isolation	69 (69.0)
**Tobacco use**	Tobacco use	24 (24.0)
**Vision**	Impaired vision	38 (38.0)

^a^ Source: self-administered HRA for Older Persons questionnaire (for definition of variables, and sources of information, see S2 Table).

At six-month follow-up, 90 participants in the intervention group and 88 participants in the control group completed the study (Fig 1). Reasons for failing to complete the study were loss to follow-up with unknown survival status (intervention group, 6; control group, 7), death (intervention group, 1; control group, 2), and withdrawal of informed consent (intervention group, 3; control group, 3). The 90 intervention group participants who completed the six-month follow-up attended most of the planned counselling sessions. On average, the number of completed counselling sessions was 5.4 per participant, with an average direct contact time between counsellor and participant of 19.7 minutes per session. As planned, control group participants continued to receive usual care. None of the control group participants received the intervention during the study period. None of the study participants experienced any harm or other unintended effects related to the study.

**Fig 2:**
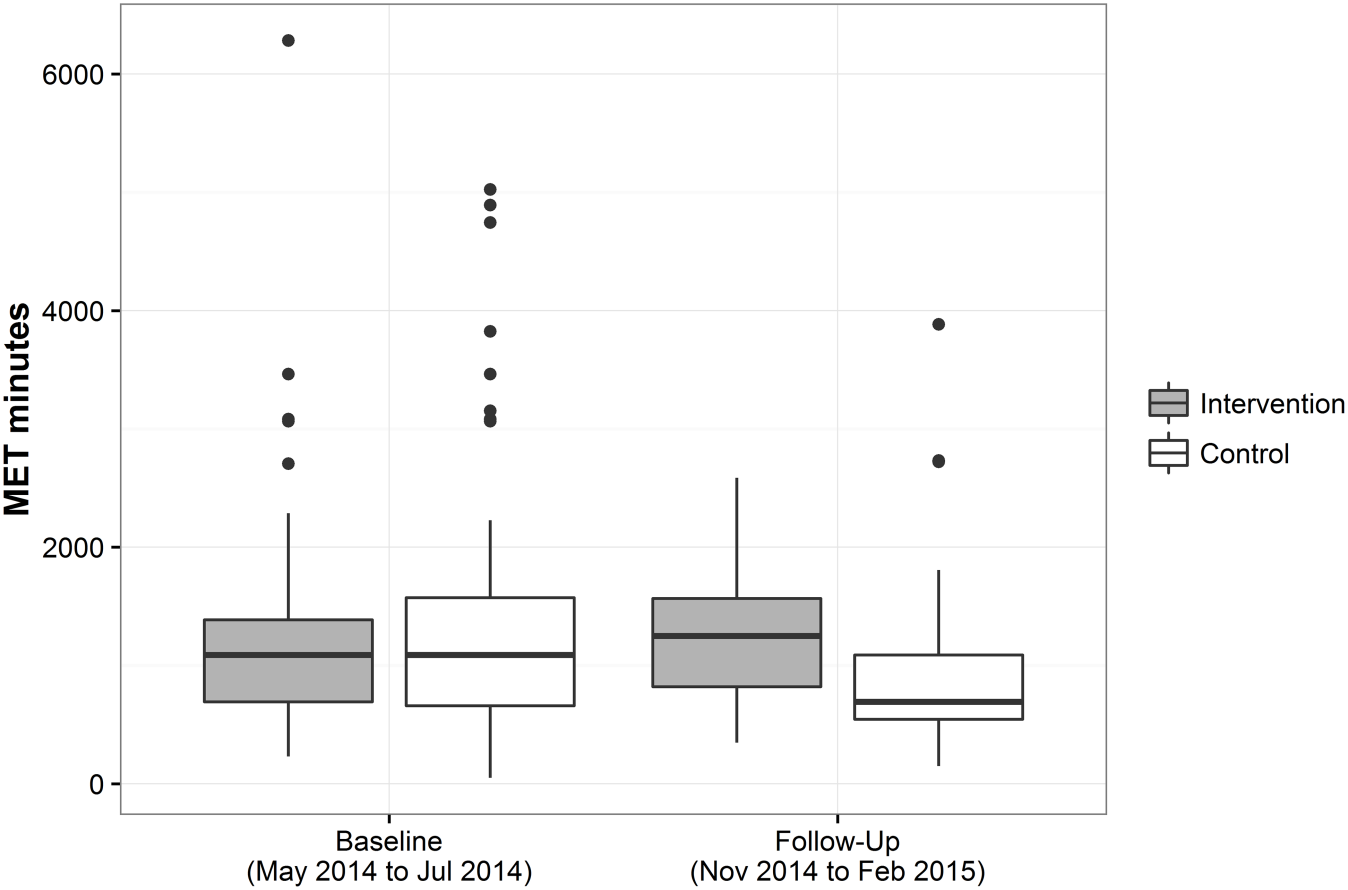
Primary outcome: Energy expenditure (MET minutes per week) in intervention and control groups at baseline (May to July 2014) and six-month follow-up (November 2014 to February 2015). Median values with interquartile range (IQR) are depicted with Tukey boxplots. The upper whisker extends from the hinge to the highest value that is within 1.5 * IQR of the hinge. The lower whisker extends from the hinge to the lowest value within 1.5 * IQR of the hinge. Data beyond the end of the whiskers are plotted as points [33].

At six-month follow-up energy expenditure was higher in the intervention group than in the control group (MET minutes per week, 1248.8, IQR 820.9–1566.0; vs. 693.0, IQR 544.5–1089.0; p< 0.001, Fig 2, Table 4). Overall, the median difference between intervention and control group was 420.0 MET minutes per week (95% CI 212.7─627.3). At follow-up, 95.6% of participants in the intervention group engaged in PA of 450 MET minutes per week (the recommended minimum of PA for adults) or more, compared to 78.4% among persons in the control group. A notable 71.1% of intervention group participants reported a level of physical activity of ≥900 MET minutes per week (i.e., at least twice the recommended minimum of PA), as compared to only 38.6% of controls.

**Table 4:** Outcomes for PA at six-month follow-up.

Parameter	Intervention group, *n* = 90	Control group, *n* = 88	OR (odds ratio)/ Δ (difference), (95% CI)	*p*-Value
**MET minutes per week ─ median (IQR)**	1248.8 (820.9–1566.0)	693.0(544.5–1089.0)	Δ: 420.0 (212.7–627.3)	<0.001
**Physical activity of ≥ 450 MET minutes per week ─ n (%)**	86 (95.6%)	69 (78.4%)	OR: 5.9 (1.9–16.7)	<0.01
**Physical activity of ≥ 900 MET minutes per week ─ n (%)**	64 (71.1%)	34 (38.6%)	OR: 3.9 (2.1–7.3)	<0.001
**Vigorous physical activity at least once per week ─ n (%)**	7 (7.8%)	4 (4.5%)	OR: 1.8 (0.5–6.3)	0.38
**Moderately vigorous or vigorous physical activity at least once per week ─ n (%)**	45 (50.0%)	19 (21.6%)	OR: 3.6 (1.9–7.0)	<0.001
**Minutes of walking per week ─ median (IQR)**	330.0 (210.0–420.0)	187.5 (158.8–229.7)	Δ: 120.0 (55.3–184.7)	<0.001
**Sitting ≥4 hours per day during last week ─ n (%)**	61 (67.8%)	73 (83.0%)	OR: 0.4 (0.2–0.9)	0.02

CI, confidence interval; IQR interquartile range; MET, metabolic equivalent of task; PA, physical activity; for definition of parameters, see Methods section.

The difference between intervention and control groups was due to an increase in PA among participants in the intervention group between baseline and follow-up and a simultaneous decrease of PA among control group participants. The median increase among participants in the intervention group was 180 MET minutes per week (95% CI 43.4 to 316.6; p = 0.01) and the median decrease in the control group 346.5 MET minutes per week (95% CI 178.4 to 514.6; p<0.001). The decrease in the control group is explained by a seasonal effect with important differences in weather conditions in Bucharest between baseline and follow-up (see Discussion section for detailed meteorological data).

The analyses revealed that the difference in PA between intervention and control groups at follow-up mainly involved walking (Table 4). Patients in the intervention group spent significantly more time walking (330.0, IQR 210.0–420.0) than did controls (187.5, IQR 158.8–330.0; p<0.001), resulting in a difference of 120 minutes (95% CI 55.3–184.7; p< 0.001) of walking per week. This is equivalent to approximately 360 MET minutes per week, which is a major proportion of the observed intervention effect of 420 MET minutes per week. A remaining small part of the intervention effect was related to a difference in the proportion of persons engaging in moderately vigorous levels of PA between intervention and control groups. Finally, intervention group participants spent less time sitting compared to persons in the control group at follow-up. The sensitivity analyses that were conducted to assess possible selection bias (inverse probability of censoring weighting) revealed similar results for primary and secondary outcomes (for detailed results, see S3 Table).

Table 5 presents the prespecified subgroup analyses. Given the limited sample size, no statistical tests are included in the table. Overall, in all subgroups median energy expenditure was higher for persons in the intervention group compared to those in the control group. This was true as well for the subgroup of participants who stated at baseline that they had no intention to increase PA.

**Table 5:** Physical activity (MET minutes per week) in various subgroups at follow-up.

Subgroup type	Definition of subgroup (*n*)	Intervention group: median (IQR)	Control group: median (IQR)
**Gender**	Women (132)	1113.0 (708.0–1566.0)	693.0 (544.5–1386.0)
	Men (46)	1386.0 (949.5–1541.2)	618.8 (346.5–1089.0)
**Income** ^a^	≥ 848 RON (138)	1386.0 (904.5–1566.0)	693.0 (544.4–1386.0)
	<848 RON (40)	1071.0 (693.0–1476.0)	544.5 (346.5–715.5)
**Intention to increase PA at baseline**	Yes (68)	1386.0 (934.5–1546.0)	693.0 (544.5–1089.0)
	No (110)	1089.0 (753.0–1566.0)	693.0 (445.5–1089.0)
**Self-perceived health status at baseline**	Good/ very good (75)	1386.0 (1089.0–1746.0)	1071.0 (693.0–1386.0)
	Fair/ poor (103)	1089.0 (693.0–1386.0)	544.5 (346.5–834.8)

IQR, interquartile range; MET, metabolic equivalent of task; PA, physical activity.

^a^848 RON is the 2014 average pension rate for Romania [37].

## Discussion

This innovative intervention combining HRA with monthly goal-oriented PA counselling in a specialized geriatric care setting resulted in substantially higher levels of PA at six-month follow-up than usual care alone in older community-dwelling adults. The exploratory subgroup analyses suggest that this effect is found for all subgroups, including persons with poor self-perceived health status, low income, and those who had no intention at baseline to increase their level of PA. The difference was mainly related to an increase in walking, and to a smaller extent to higher levels of moderately vigorous activities.

This study observed a seasonal effect with a substantial decline of PA among control group participants between baseline and follow-up. According to official meteorological data, the summer months May to July 2014 (the time period when baseline assessments took place) were mostly mild, with hot air temperatures >30°C on only 12 out of 90 days. In contrast, the winter months November 2014 to February 2015 (the follow-up period) were mostly cold and wet with snow and ice, and air temperatures at or below the freezing point on 75 of 120 days and precipitation on 53 of 90 days (meteorological information from National Administration of Meteorology, Meteo Romania, Bucharest, Romania (for details see S4 Text). Levels of PA change in regions with greater seasonal variation in temperature or precipitation [38–40], and outdoor walking, a highly weather sensitive activity, was the main type of participants’ PA. It is therefore remarkable that PA among persons in the intervention group significantly increased from baseline to follow-up despite this seasonal effect.

Given the high prevalence of health risks and problems in the elderly, the potential for PA counselling based upon HRA information is high. Yet many older persons report health-related barriers to increasing PA, and/or a lack of awareness that they might benefit from an increase in PA. To realize counselling’s potential for increasing PA, counsellors need time sufficient to build mutual trust with the older participant and work out the specific pros and cons of PA with each one. That our study’s effect on PA was stronger than those reported in both previous HRA-based studies [20–22] and interventions designed to increase PA among older persons [16] is most likely explained by its unique combination of an initial multidimensional assessment with strong theory-based and highly individualized counselling.

Among this study's limitations is a possible selection effect. Compared to international data, baseline levels of PA were relatively high in the study population: about 80% of participants exceeded the minimum recommended level of 450 MET minutes per week of PA at baseline. This may have arisen from the fact that patients referred for specialized geriatric care were in general well-educated and motivated for health promotion. Although this possible selection bias and the single-centre design limit generalisability of our findings, the study results were consistent across persons with varying socioeconomic backgrounds, health status, and initial motivation for behaviour change.

A further limitation may be located in participant self-reporting of PA with the IPAQ [41–45]. Direct outcome measurement with an accelerometer was not possible due to budgetary constraints. A recent systematic review of the measurement properties of self-report PA questionnaires found good to excellent reliability of the IPAQ but concluded that its validity requires further investigation [45]. The review also showed that IPAQ validity partly depends on the version of the IPAQ, and may vary by IPAQ domain. For example, the IPAQ short form covering the “last 7 days” (i.e., the questionnaire version used in our study) has shown good agreement for the IPAQ walking component if compared with accelerometer data [45], a relevant finding since walking was the main type of PA observed in our study. Most previous IPAQ validation studies have been conducted in samples of relatively young adults, and only few studies included healthy older persons. One such study suggested fair validity if used in older persons, with positive correlations (r = 0.28 to 0.47) between self-report and accelerometer data (sitting, walking, and both moderate and vigorous PA) [42]. Most studies evaluated the IPAQ in the English language and translations may lead to inconsistent results. However, the Romanian version used in our study was based on a translation/ back-translation approach to avoid inconsistencies in translation. In summary, use of the self-report IPAQ results in some uncertainty about the validity of the absolute values of energy expenditure levels, but it is unlikely that self-reporting led to biased estimates of intervention effects in our study. A further limitation may involve follow-up which was not possible for all participants. The number of participants lost to follow-up was small, though, and sensitivity analyses suggest that this did not affect results. It seems unlikely that other problems impede the internal validity of the main study findings (e.g., leakage, maturation, testing bias). Finally, this study was designed to explore the effects of a short-term intervention and short-term effects on PA, and did not have a long-term perspective. From other intervention studies it is known that PA interventions have to be continued over the long-term to attain sustained effects [25]. Yet this study's promising outcome suggests its short-term scope was less a limitation than a time frame appropriate for obtaining evidence to motivate in-depth investigation of this kind of PA intervention over the long term.

In spite of these limitations, we believe this study can inform clinical interventions to improve PA. The standardised HRA approach has been shown to be feasible in diverse health care settings in several European countries and the U.S at low cost [19, 29]. Replication of this method of HRA and PA counselling should align with a setting's health care system by involving health professionals in the system who care for older adults, thereby also ensuring that the intervention is pursued for longer-term follow-up. For practice implementation, relying on senior clinicians for health counselling in the long run is probably not feasible due to cost and resource constraints. However, counselling can be organised in the general practice system with nurses or other health professionals as counsellors [46]. Reinforcement via programmes implemented on mobile communication and data acquisition platforms may be added to further focus and increase the efficacy of PA counselling [46,47]. The finding that HRA with counselling has strong effects on PA in older persons is promising, given the fact that HRA has been shown to result in favourable long-term health benefits in nondisabled older persons [22]. Future studies can refine the intervention methodology and test the reproducibility of findings in other settings.

We conclude that using HRA to inform individualized PA counselling is a promising method for achieving improvements in PA, and ultimately health and longevity among large groups of community-dwelling older persons.

## Supporting information

S1 Table Description of key elements of the intervention, classified according to Michie et al. (2013). (DOCX)

S2 Table Instruments for the assessment and definition of concomitant health-related problems and risk factors identified with the HRA for older persons questionnaire. (PDF)

S3 Table Outcomes for physical activity at six-month follow-up using inverse probability of censoring weighting (sensitivity analysis). (PDF)

S1 Text Ethical approval. (PDF)

S2 Text Study protocol. (PDF)

S3 Text Analytic plan. (PDF)

S4 Text Information from National Administration of Meteorology, Bucharest. (PDF)

S5 Text Completed CONSORT checklist. (PDF)

S1 Data Minimal dataset underlying the study findings: Codebook. (XLSX)

S2 Data Minimal dataset underlying the study findings: Anonymyzed individual participant data. (CSV)

## Abbreviations

ADL: activities of daily living
CI: confidence interval
HRA: health risk assessment
IPAQ: International Physical Activity Questionnaire
IQR: interquartile range
MET: metabolic equivalent of task
OR: odds ratio
PA: physical activity

## References

[1] US Department of Health and Human Services. 2008 Physical Activity Guidelines for Americans. PDPHP Publication No. U0036, October 2008. http://www.health.gov/paguidelines

[2] World Health Organization. Global recommendations on physical activity for health. WHO Library Cataloguing-in-Publication Data Geneva, 2010 ISBN 978 92 4 159 997 9. http://apps.who.int/iris/bitstream/10665/44399/1/9789241599979_eng.pdf

[3] OECD/EU. Health at a glance: Europe 2016 –State of health in the EU cycle, OECD Publishing, Paris http://dx.doi.org/10.1787/9789264265592-en

[4] European Commission. Eurostat. Data exported on March 2, 2017. http://ec.europa.eu/eurostat/web/health/health-status-determinants/data/database

[5] Kohl HW, Craig CL, Lambert EV, Inoue S, Alkandari JR, Leetongin G, et al The pandemic of physical inactivity: global action for public health. Lancet. 2012;380:294–305. doi: 10.1016/S0140-6736(12)60898-8 22818941 10.1016/S0140-6736(12)60898-8

[6] Lee IM, Shiroma EJ, Lobelo F, Puska P, Blair SN, Katzmarzyk PT, et al Effect of physical inactivity on major non-communicable diseases worldwide: an analysis of burden of disease and life expectancy. Lancet. 2012;380:219–229. doi: 10.1016/S0140-6736(12)61031-9 22818936 10.1016/S0140-6736(12)61031-9 PMC3645500

[7] Pahor M, Guralnik JM, Ambrosius WT, Blair S, Nonds DE et al Effect of structured physical activity on prevention of major mobility disability in older adults. JAMA. 2014;311:2387–2396. doi: 10.1001/jama.2014.5616 24866862 10.1001/jama.2014.5616 PMC4266388

[8] van der Vorst A, Zijlstra GAR, De Witte N, Duppen D, Stuck AE, Kempen GIJM, et al Limitations in activities of daily living in community-dwelling people aged 75 and over: a systematic literature review of risk and protective factors. PLoS ONE. 2016;11:e016512: doi: 10.1371/journal.pone.0165127 27760234 10.1371/journal.pone.0165127 PMC5070862

[9] Tan ZS, Spartano NK, Beiser AS, DeCarli C, Auerbach SH, Vasan RS et al Physical activity, brain volume, and dementia risk: The Framingham Study. J Gerontol A Biol Sci Med Sci. 2017, Vol. 72, No. 6, 789–795 doi: 10.1093/gerona/glw130 27422439 10.1093/gerona/glw130 PMC6075525

[10] Black SV, Cooper R, Martin KR, Brage S, Kuh D, Strafford M. Physical activity and mental well-being in a cohort aged 60–64 years. Am J Prev Med. 2015;49:172–180. doi: 10.1016/j.amepre.2015.03.009 26070782 10.1016/j.amepre.2015.03.009 PMC4518501

[11] Kendrick D, Kumar A, Carpenter H, Zijlstra GA, Skelton DA, Cook JR et. al Exercise for reducing fear of falling in older people living in the community. Cochrane Database Syst Rev. 2014;11:CD009848 doi: 10.1002/14651858.CD009848.pub2 25432016 10.1002/14651858.CD009848.pub2 PMC7388865

[12] Greenwood-Hickman MA, Rosenberg DE, Phelan EA, Fitzpatrick AL. Participation in older adult physical activity programs and risk for falls requiring medical care, Washington State, 2005–2011. Prev Chronic Dis. 2015; 12:E90 doi: 10.5888/pcd12.140574 26068411 10.5888/pcd12.140574 PMC4467255

[13] Arem H, Moore SC, Patel A, Hartge P, Berrington de Gonzalez A, Visvanathan K et al Leisure time physical activity and mortality: a detailed pooled analysis of the dose-response relationship. JAMA Intern Med. 2015;175(6):959–967. doi: 10.1001/jamainternmed.2015.0533 25844730 10.1001/jamainternmed.2015.0533 PMC4451435

[14] Franco MR, Tong A, Howard K, Sherrington C, Ferreira PH, Pinto RZ, Ferreira ML. Older people's perspectives on participation in physical activity: a systematic review and thematic synthesis of qualitative literature. Br J Sports Med. 2015;49:1268–1276. doi: 10.1136/bjsports-2014-094015). 25586911 10.1136/bjsports-2014-094015

[15] Jefferis BJ, Sartini C, Lee IM, Choi M, Amuzu A, Gutierrez C. Adherence to physical activity guidelines in older adults, using objectively measured physical activity in a population-based study. BMC Public Health. 2014;14:382 doi: 10.1186/1471-2458-14-382 24745369 10.1186/1471-2458-14-382 PMC4021412

[16] Chase JD. Interventions to increase physical activity among older adults: a meta-analysis. The Gerontologist. 2015;55: 706–718. doi: 10.1093/geront/gnu090 25298530 10.1093/geront/gnu090 PMC4542588

[17] Fries JF, McShane D. Reducing need and demand for medical services in high-risk persons. A health education approach. West J Med. 1998;169:201–207. 9795579 PMC1305287

[18] Breslow L, Beck JC, Morgenstern H, Fielding JE, Moore AA, Carmel M, et al Development of a health risk appraisal for the elderly (HRA-E). Am J Health Promot. 1997; 11:337–343. 10167368 10.4278/0890-1171-11.5.337

[19] Oremus M, Hammill A, Raina P. Health risk appraisal. Technology Assessment Report. McMaster University Evidence-based Practice Center, Hamilton, Canada, 2011. https://www.cms.gov/Medicare/Coverage/DeterminationProcess/downloads/id79ta.pdf

[20] Harari D, Iliffe S, Kharicha K, Egger M, Gillmann G, von Renteln-Kruse W, et al Promotion of health in older people: a randomised controlled trial of health risk appraisal in British general practice. Age and Aging. 2008;37:565–571. doi: 10.1093/ageing/afn150 18755784 10.1093/ageing/afn150

[21] Dapp U, Anders JA, von Renteln-Kruse W, Minder CE, Meier-Baumgartner HP, Swift CG, et al A randomized trial of effects of health risk appraisal combined with group sessions or home visits on preventive behaviors in older adults. J Gerontol A Biol Sci Med Sci. 2011;66:591–598. doi: 10.1093/gerona/glr021 21350242 10.1093/gerona/glr021

[22] Stuck AE, Moser A, Morf U, Wirz U, Wyser J, Gillmann G, et al Effect of health risk assessment and counselling on health behaviour and survival in older people: A pragmatic randomised trial. PLoS Med. 2015; 12(10): e1001889 doi: 10.1371/journal.pmed.1001889 26479077 10.1371/journal.pmed.1001889 PMC4610679

[23] Pears S, Morton K, Bijker M, Sutton S, Hardeman W. Development and feasibility study of very brief interventions for physical activity in primary care. BMC Public Health. 2015;15(1), 333 doi: 10.1186/s12889-015-1703-8 25887643 10.1186/s12889-015-1703-8 PMC4451719

[24] Michie S, Richardson M, Johnston M, Abraham C, Francis J, Hardeman W, et al The behavior change technique taxonomy (v1) of 93 hierarchically clustered techniques: building an international consensus for the reporting of behavior change interventions. Ann Behav Med. 2013;46(1), 81–95. doi: 10.1007/s12160-013-9486-6 23512568 10.1007/s12160-013-9486-6

[25] Foster C, Hillsdon M, Thorogood M, Kaur A, Wedatilake T. Interventions for promoting physical activity. The Cochrane Library. 2005 CD003180 doi: 10.1002/14651858.CD003180.pub2 15674903 10.1002/14651858.CD003180.pub2 PMC4164373

[26] Prochaska JO, Velicer WF. The transtheoretical model of health behavior change. Am J Health Promot. 1997;12:38–48. 10170434 10.4278/0890-1171-12.1.38

[27] Folstein MF, Folstein SE, McHugh PR. “Mini-mental state”. A practical method for grading the cognitive state of patients for the clinician. J Psychiatr Res. 1975;12:189–198. 1202204 10.1016/0022-3956(75)90026-6

[28] Katz S, Ford AB, Moskowitz RW, Jackson BA, Jaffe MW: Studies of illness in the aged. The index of ADL: A standardized measure of biological and psychosocial function. JAMA. 1963, 85:914–919. 10.1001/jama.1963.03060120024016 14044222

[29] Stuck AE, Kharicha K, Dapp U, Anders J, von Renteln-Kruse W, Meier-Baumgartner HP, et al Development, feasibility and performance of a health risk appraisal questionnaire for older persons. BMC Medical Research Methodology. 2007;7:1 doi: 10.1186/1471-2288-7-1 17217545 10.1186/1471-2288-7-1 PMC1783663

[30] Craig CL, Marshall AL, Sjöström M, Bauman AE, Booth ML, Ainsworth BE, et al International Physical Activity Questionnaire: 12-country reliability and validity. Med Sci Sports Exerc. 2003;35:1381–1395. doi: 10.1249/01.MSS.0000078924.61453.FB 12900694 10.1249/01.MSS.0000078924.61453.FB

[31] Guidelines for data processing and analysis of the International Physical Activity Questionnaire (IPAQ)–Short and long forms. https://docs.google.com/viewer?a=v&pid=sites&srcid=ZGVmYXVsdGRvbWFpbnx0aGVpcGFxfGd4OjE0NDgxMDk3NDU1YWRlZTM 25376692

[32] Christmas C, Andersen RA. Exercise and older patients: Guidelines for the Clinician. J Am Geriatr Soc. 2000;48:318–324. 10733061 10.1111/j.1532-5415.2000.tb02654.x

[33] Koenker R, Bassett G. Regression quantiles. Econometrica. 1978;46:33–50.

[34] Robins JM, Hernán MA, Brumback B. Marginal structural models and causal inference in epidemiology. Epidemiology. 2000;11:550–560. 10955408 10.1097/00001648-200009000-00011

[35] Weuve J, Tchetgen Tchetgen EJT, Glymour MM, Beck TL, Aggarwal NT, et al Accounting for bias due to selective attrition: the example of smoking and cognitive decline. Epidemiology. 2012;23:119–128. doi: 10.1097/EDE.0b013e318230e861 21989136 10.1097/EDE.0b013e318230e861 PMC3237815

[36] Wickham H. ggplot2: Elegant graphics for data analysis. 1st ed New York: Springer; 2009.

[37] National Institute of Statistics. Press Release No.225 Sept. 2014. Number of pensioners and medium monthly pension in second trimester 2014. http://www.insse.ro/cms/ro/content/numarul-de-pensionari-si-pensia-medie-lunara

[38] Merchant AT, Dehghan M, Akhtar-Danesh N. Seasonal variation leisure-time physical activity among Canadians. Can J Publ Health. 2007;98:203–208. URL: http://www.jstor.org/stable/41994912. 10.1007/BF03403713 PMC6975733 17626385

[39] Shephard RJ, Aoyagi Y. Seasonal variations in physical activity and implications for human health. Eur J Appl Physiol. 2009;107:251–271. doi: 10.1007/s00421-009-1127-1 19609553 10.1007/s00421-009-1127-1

[40] Uitenbroek DG. Seasonal variation in leisure time physical activity. Med Sci Sports Exerc. 1993;25:755–760. 8321115

[41] Tomioka K., Iwamoto J., Saeki K., & Okamoto N. (2011). Reliability and validity of the International Physical Activity Questionnaire (IPAQ) in elderly adults: the Fujiwara-kyo Study. J Epidemiol. 2011; 21(6), 459–465. doi: 10.2188/jea.JE20110003 21946625 10.2188/jea.JE20110003 PMC3899462

[42] Hurtig-Wennlöf A., Hagströmer M., & Olsson L. A. (2010). The International Physical Activity Questionnaire modified for the elderly: aspects of validity and feasibility. Public Health Nutrition. 2010;13, 1847 doi: 10.1017/S1368980010000157 20196910 10.1017/S1368980010000157

[43] Warren JM, Ekelund U, Besson H, Mezzani A, Geladas N, Vanhees L. Assessment of physical activity: a review of methodologies with reference to epidemiological research: a report of the exercise physiology section of the European Association of Cardiovascular Prevention and Rehabilitation. Eur J Cardiovasc Prev Rehab. 2010;17:127 doi: 10.1097/HJR.0b013e32832ed875 20215971 10.1097/HJR.0b013e32832ed875

[44] Lee PH, Macfarlane DJ, Lam TH, Stewart SM. Validity of the International Physical Activity Questionnaire Short Form (IPAQ-SF): A systematic review. Int J Behav Nutr Physical Activity. 2011;8:115 doi: 10.1186/1479-5868-8-115 22018588 10.1186/1479-5868-8-115 PMC3214824

[45] Silsbury Z, Goldsmith R, Rushton A. Systematic review of the measurement properties of self-report physical activity questionnaires in healthy adult populations. BMJ Open 2015;5:e008430 doi: 10.1136/bmjopen-2015-008430 26373402 10.1136/bmjopen-2015-008430 PMC4577932

[46] Harris T, Kerry SM, Victor CR, Ekelund U, Woodcock A, Iliffe S, et al A primary care nurse-delivered walking intervention in older adults: PACE (Pedometer Accelerometer Consultation Evaluation)-Lift cluster randomised controlled trial. PLoS Med. 2015;12:e1001783 doi: 10.1371/journal.pmed.1001783 25689364 10.1371/journal.pmed.1001783 PMC4331517

[47] King AC, Hekler EB, Grieco LA, Winter SJ, Sheats JL, Buman MP, et al Effects of three motivationally targeted mobile device applications on initial physical activity and sedentary behaviour change in midlife and older adults: a randomized trial. PLoS ONE. 2016;11(6):e0156370 doi: 10.1371/journal.pone.0156370 27352250 10.1371/journal.pone.0156370 PMC4924838

