# Supplementary material for: Effects of health risk assessment and counselling on physical activity in older people: A pragmatic randomised trial
Source: PLoS One. 2017 Jul 20;12(7):e0181371. doi: 10.1371/journal.pone.0181371 (PMC5519086; doi:10.1371/journal.pone.0181371)
Supplement: S1 Table — (DOCX) [file pone.0181371.s001.docx]

**S1 Table. Description of key elements of the intervention, classified according to Michie et al.***

| **Intervention component** | **Definition in the context of the present study** | **General approach used by counsellor** | **Role of information from the participants’ responses to the HRA for older persons questionnaire, and examples of how counsellor implemented the intervention component** |
| --- | --- | --- | --- |
| **5.1. Information about health consequences** | Provide information (written, verbal, visual) about health consequences of performing the behaviour. | Explains that physical inactivity is a risk factor for cardiovascular morbidity and mortality, while PA has favourable effects on multiple outcomes in old age (such as on sleep, mood, cognition, functional reserve). Emphasizes PA risks and benefits in the context of the older person’s comorbidities. | If a person had an increased cardiovascular risk due to hypertension, then the counsellor emphasized the importance of reducing the cardiovascular risk by a combination of blood pressure control and by maintenance of a high level of PA. If a person suffered from sleep problems, the counsellor emphasized the favourable impact of PA on sleep. |
| **1.1. Goal setting (behaviour)** | Set or agree on a goal defined in terms of the behaviour to be achieved. | Proposes a goal, taking into account previous types of PA, current level of PA, and relevant factors influencing goal setting. Selects the type of PA recommended based on the type of comorbidity (e.g., osteoporosis, pulmonary disease, pain), the patient’s functional reserve (e.g., mobility limitation, sensory impairment), and environmental and other factors. | If a person reported vigorous PA once a week, the counsellor explored the potential for increasing the frequency of this type of PA. If a person reported mostly sedentary behaviour, then moderate PA type activities were considered as a first step. The counsellor also took into account the pattern of previous PA of the older person. Or, if a person had osteoporosis, then the counsellor gave a special emphasis on proposing weight-bearing exercise and prevention of falls. |
| **1.2. Problem solving** | Analyse factors influencing the behaviour and generate or select strategies that include overcoming barriers and/or increasing facilitators. | Identifies barriers for increasing PA and initiates steps to overcome these barriers. | The counsellor prescribed a pain medication to a patient with impairments due to pain, or explained what type of PA may not increase pain, or recommended use of walking aid in a patient with walking difficulty. |
| **3.2. Social support (practical)** | Advise on, arrange, or provide practical help. | Advises patient to find someone for help with transportation when going to exercise group. | If the person reported social isolation, then the counsellor developed a plan with the patient on how the patient might be able to find someone to help him/her with transportation. |
| **3.3. Social support (emotional)** | Advise on, arrange, or provide emotional social support. | Advises patient to find a companion for walking once a week. | If the patient had a good neighbourhood network but limited family network, the counsellor proposed a plan for how the patient could persuade his/her neighbour to go out for a walk with him/her once a week. |

| **1.4. Action planning** | Agree upon a concrete and detailed plan of PA behaviour (including type, context, frequency, duration). | Defines a detailed daily and weekly schedule for PA. When making the action plan, takes into account other health priorities of the patient. | If the patient needed vision correction, the counsellor gave priority to the vision correction at the first counselling session, and emphasized PA recommendations once this initial issue had been resolved. |
| --- | --- | --- | --- |
| **1.8. Behavioural contract** | Create a written specification of the behaviour to be performed, agreed on by the person, and witnessed by another. | Writes down the agreed upon PA plan, and gives this plan to the patient like a prescription. | After each counselling session, the counsellor gave a personal written plan to the patient with the agreed-upon actions of the patient. |
| **1.5. Review behaviour goal(s)** | Review behaviour goal(s) jointly with the person and consider modifying goal(s) or behaviour change strategy in light of achievement. | At follow-up counselling session, reviews goals, with resetting, modifying, or redefining goals as needed. | At each counselling session, the counsellor explored whether the patient reported any change in health or psychosocial situation (compared with HRA-based information), and reviewed implementation status of the behaviour-change goals. Then the counsellor decided, in agreement with the patient, whether the goals should remain unchanged, or whether goal modification or new goal definition were needed. |
| **8.4 Habit reversal** | Identify patient’s habits related to sedentary behaviour. | Asks the patient about his/her usual daily activities. Identifies preferred and repeated activities related to sedentary behaviour and points them out to the patient. | If the patient spent a lot of time sitting, watching television, she/he was advised to limit this time to an hour at a time and to use the commercial breaks for short walks or exercises. |
| **8.3 Habit formation** | Identify ways to incorporate PA into patients’ daily routine. | Asks the patient about his/her usual daily schedule and activities. Together with the patient, identifies methods of incorporating PA into daily routine. | The counsellor suggested methods of increasing PA based on patients’ preferences and situation. If the patient reported “I have no one to do it [PA] with,” or “I do not know any opportunities for activity suitable for my age group,” the counsellor explored whether the person might be interested in adopting a dog and take it for a walk twice a day. If the patient reported that she/he frequently “felt downhearted and low” the counsellor advised the person to go out for a walk for a minimum of 30 minutes every time she/he felt that way. |

*Michie, S., Richardson, M., Johnston, M., Abraham, C., Francis, J., Hardeman, W. et al. (2013). The behaviour change technique taxonomy (v1) of 93 hierarchically clustered techniques: building an international consensus for the reporting of behaviour change interventions. Annals of Behavioral Medicine, 46(1), 81-95. doi:10.1007/s12160-013-9486-6
