# Supplementary material for: Effects of health risk assessment and counselling on physical activity in older people: A pragmatic randomised trial
Source: PLoS One. 2017 Jul 20;12(7):e0181371. doi: 10.1371/journal.pone.0181371 (PMC5519086; doi:10.1371/journal.pone.0181371)
Supplement: S2 Table — (PDF) [file pone.0181371.s002.pdf]

S2 Table. Instruments for the assessment of, and definition of concomitant health-related problems and risk factors identified with the HRA for older persons questionnaire

Risk factor domain	Instrument for assessment	Definition of risk	Description
**Activities of daily living (ADL)**	Basic ADL [Katz, 1969]	Difficulty/ need for human assistance in ≥ 1 item of basic activities of daily living	Impaired basic ADL
	Instrumental ADL [Lawton and Brody, 1969]	Difficulty/ need for human assistance in ≥ 1 item of instrumental activities of daily living (IADL)	Impaired instrumental ADL
**Alcohol use**[a]	Alcohol Use Disorders Identification Test [Babor et al., 1992]	Drinking more than age- and gender-specific limits of quantity and frequency of alcohol[b]	Possible hazardous alcohol use
**Chronic conditions**	Chronic Conditions [Human Population Laboratory, 1965]	Self-reported high blood pressure	High blood pressure
		Self-reported high cholesterol	High cholesterol
		Self-reported high diabetes	Diabetes
		Self-reported coronary heart disease/ heart failure	Heart disease
		Self-reported stroke	Stroke
		Self-reported chronic bronchitis/ asthma	Pulmonary disease
		Self-reported osteoporosis	Osteoporosis
		Self-reported arthritis or rheumatism	Rheumatism
**Falls**	Study of Osteoporotic Fractures Research Group Survey [Kelsey et al., 1992]	Repeated (≥1) falls in past 12 months	Repeated falls
	Fear of falling [Tinetti et al., 1988]	Self-reported limitation of activities due to fear of falling	Fear of falling
**Hearing**	Hearing Handicap Inventory for the Elderly [Lichtenstein et al., 1988]	Impaired hearing (self-perceived hearing fair/ poor/ deaf)	Impaired hearing
**Incontinence**	Medical, Epidemiological and Social Aspects of Aging Project Questionnaire [Diokno et al., 1986]	Urinary incontinence on >5 days in past 12 months	Urinary incontinence
**Memory**	Memory Self Report [Riege, 1982]	Memory problems	Memory problem
**Mood**	5-item Mental Health Inventory Screening Test [Stewart et al., 1988]	Depressive mood	Possible depression
**Nutrition**	Self-reported height and weight	Body mass index <20 kg/m^2	Underweight
	Self-reported height and weight	Body mass index ≥ 27 kg/m^2	Overweight
**Pain**	Geriatric Pain Measure [Ferrell et al., 2000]	Presence of moderate to severe pain	Moderate or severe pain
**Social factors**	Medical Outcomes Study Social Support Survey [Sherbourne and Stewart, 1991]	Low level of emotional support	Low emotional support

	Lubben Social Network Scale [Lubben et al., 1988]	High risk of social isolation	Risk for social isolation
**Tobacco use**	Tobacco Use Questionnaire [Breslow et al., 1997]	Current tobacco uses	Tobacco use
**Vision**	Visual Functioning Questionnaire [Mangione et al., 1998]	Impaired vision (self-perceived vision fair/ poor/ blind)	Impaired vision

[a] Possible hazardous alcohol was defined as drinking more than age- and gender-specific limits of quantity and frequency of alcohol (men < 70 years >14 drinks per week, men >70 years >11 drinks per week, women <70 years >11 drinks per week, women >70 years >8 drinks per week), or as meeting the criteria of binge drinking (>4 drinks at one occasion monthly or more frequently).

References (in alphabetical order):

Atchison KA, Dolan TA. Development of the Geriatric Oral Health Assessment Index. J Dent Education. 1990;54(11):680-687.

Babor TF, de la Fuente JR, Saunders J, Grant M: AUDIT – The Alcohol Use Disorders Identification Test: guidelines for use in primary health care. Geneva: World Health Organization; 1992.http://whqlibdoc.who.int/hq/1992/who_psa_92.4.pdf (Accessed March 16, 2015).

Beers MH. Explicit criteria for determining potentially inappropriate medication use by the elderly. Arch Intern Med. 1997;157(14):1531-1536.

Berkman LF, Syme SL. Social networks, host resistance, and mortality: A nine-year follow-up study of Alameda County residents. Am J Epidemiol. 1979;109(2):186-204.

Breslow L, Beck JC, Morgenstern H, Fielding JE, Moore AA, Carmel M, Higa J. Development of a health risk appraisal for the elderly (HRA-E). Am J Health Promot. 1997;11(5):337-343.

Exerc. 2003;35:1381-1395.Diokno AC, Brock BM, Brown MB, Herzog AR. Prevalence of urinary incontinence and other urological symptoms in the noninstitutionalized elderly. J Urol. 1986;136(5):1022-1025.

Ferrell BA, Stein WM, Beck JC. The Geriatric Pain Measure: Validity, reliability and factor analysis. J Am Geriatr Soc. 2000;48(12):1669-1673.

Fried LP, Bandeen-Roche K, Chaves PH, Johnson BA. Preclinical mobility disability predicts incident mobility disability in older women. J Gerontol. 2000;55(1):M43-M52.

Human Population Laboratory. Health and Ways of Living, Human Population Laboratory (HPL) 1965 Men's Form.

Katz S, Ford AB, Moskowitz RW, Jackson BA, Jaffe MW: Studies of illness in the aged. The index of ADL: A standardized measure of biological and psychosocial function. JAMA 1963, 85:914-919.

Kelsey JL, Browner WS, Seeley DG, Nevitt MC, Cummings SR. Risk factors for fractures of the distal forearm and proximal humerus. Am J Epidemiol. 1992;135(5):477-489.

Lawton MP, Brody EM. Assessment of older people: Self-maintaining instrumental activities of daily living. Gerontologist. 1969;9(3):179-186.

Lichtenstein MJ, Bess FH, Logan SA. Validation of screening tools for identifying hearing-impaired elderly in primary care. JAMA. 1988;259(19):2875-2878.

Lubben JE. Assessing social networks among elderly populations. Fam Comm Health. 1988;11(3):42-52.

Mangione CM, Lee PP, Pitts J, Gutierrez P, Berry S, Hays RD. Psychometric properties of the National Eye Institute Visual Function Questionnaire (NEI-VFQ). Arch Ophthalmol. 1998;116(11):1496-1504.

National Center for Chronic Disease Prevention and Health Promotion. Behavioral Risk Factor Survey. Department of Health and Human Services, 1993.

Riege WH. Self-report and tests of memory aging. Clin Gerontol. 1982;1(2):23-36.

Sherbourne CD, Stewart AL. The MOS social support survey. Soc Sci Med. 1991;32(6):705-714.

Stewart AL, Hays RD, Ware JE. The MOS short-form general health survey. Reliability and validity in a patient population. Med Care. 1988;26(7):724-732.

Stoy DB, Curtis RC, Dameworth KS, Dowdy AA, Hegland J, Levin JA, Sousoulas BG. The successful recruitment of elderly black subjects in a clinical trial: the CRISP experience. Cholesterol Reduction in Seniors Program. J Natl Med Assoc. 1995;87(4):280-287.

Tinetti ME, Speechley M, Ginter SF. Risk factors for falls among elderly persons living in the community. N Engl J Med. 1988; 319(26):1701-1707.

Wasson J, Nierenberg D, Landgraf J, Whaley F, Malenka, D. Johnson D, Keller A, Dartmouth Primary Care COOP The effect of a patient questionnaire on drug-related symptoms in elderly outpatients. Ann Rev Geriatr Gerontol. 1992(12):109-125.
