# Supplementary material for: Effects of health risk assessment and counselling on physical activity in older people: A pragmatic randomised trial
Source: PLoS One. 2017 Jul 20;12(7):e0181371. doi: 10.1371/journal.pone.0181371 (PMC5519086; doi:10.1371/journal.pone.0181371)
Supplement: S3 Table — (PDF) [file pone.0181371.s003.pdf]

S3 Table. Outcomes for PA at six-month follow-up using inverse probability of censoring weighting [a].

Parameter	Intervention group *n*=90	Control group *n*=88	OR (odds ratio)/ Δ difference (95% CI)	*p*-Value
**MET minutes per week – median (IQR)**	1248.8 (IQR 745.1)	693.0 (IQR 544.5)	Δ: 420.0 (194.4-645.6)	< 0.001
**Vigorous physical activity at least once per week – n (%)**	7 (7.8%)	4 (4.5%)	OR: 1.8 (0.5-6.4)	0.36
**Moderately vigorous or vigorous physical activity at least once per week – n (%)**	45 (50.0%)	19 (21.6%)	OR: 3.7 (1.9-7.3)	<0.001
**Minutes of walking per week – median (IQR)**	330.0 (IQR 210.0)	187.5 (IQR 171.2)	Δ: 165.0 (99.5-230.5)	< 0.001
**Sitting ≥4 hours per day during the last week – n (%)**	61 (67.8%)	73 (83.0%)	OR: 0.4 (0.2-0.9)	0.02

CI, confidence interval; IQR interquartile range; MET, metabolic equivalent of task; PA, physical activity; for definition of parameters, see Methods section.

[a] Inverse probability of censoring weights ranged from 1.0 to 3.8 (mean 1.2 ± SD 0.4). Weights were constructed using a logistic model with outcome being lost-to-follow-up using covariates gender, age and MET minutes per week.
