# Supplementary material for: Effects of health risk assessment and counselling on physical activity in older people: A pragmatic randomised trial
Source: PLoS One. 2017 Jul 20;12(7):e0181371. doi: 10.1371/journal.pone.0181371 (PMC5519086; doi:10.1371/journal.pone.0181371)
Supplement: S1 Text — (PDF) [file pone.0181371.s004.pdf]

Institutul Natiuonal de Gerontologie si Geriatrie "Ana Aslan"
Caldarusani, nr. 9, sect. 1, Bucuresti, Romania
Tel.: 0318059300; 0318059301; 0318059302; 0318059303; Fax: 0212231480
e-mail: manager@ana-aslan.ro; geriatrie@ana-aslan.ro

MINISTERUL SĂNĂTĂȚII
INSTITUTUL NATIONAL DE
GERONTOLOGIE ȘI GERIATRIE
"ANA ASLAN"
INTRARE Nr. 972
2014 Luna 01 Ziua 28

CERTIFICAT DE APROBARE
Comisia de etica cercetarii

Comisia de etica cercetarii din cadrul Institutului National de Gerontologie si Geriatrie "Ana Aslan" s-a reunit in sedinta de lucru in data de 28.01.2014, a analizat si a aprobat solicitarea scrisa (Cerere nr. 972 / 28.01.2014) a domnului Dr. Prada Gabriel, director de proiect, cu privire la avizarea desfasurarii proiectului de cercetare intitulat "MEDICAL RISK ASSESSEMENT AND HEALTH EDUCATION IN OLDER PEOPLE". Proiectul se va dasfasura in cadrul INGG-Ana Aslan, pe o perioada de 14 luni, incepand cu luna ianuarie 2014. Studiul este un trial clinic controlat randomizat, realizat in colaborare cu Universitatea de Geriatrie din Berna, Elvetia, care este si coordonatorul proiectului.

Obiectivele principale ale studiului sunt:

- evaluarea riscului medical la persoanele de 65 ani si peste, precum si oferirea de recomandari specifice in vederea corectarii comportamentelor legate de stilul de viata
- evaluarea fezabilitatii si acceptabilitatii chestionarului HRA-O, adaptat cultural pentru Romania.

Lotul analizat va cuprinde 400 de pacienti care vor indeplini criteriile de includere in studiu, si isi vor da consimtamantul in scris dupa informarea acestora cu privire la obiectivele studiului. Toti pacientii vor completa un chestionar de apreciere a riscului pentru sanatate la persoane adulte batrane (HRA-O) prin raspunsuri legate de numele lor, datele de contact, varsta, sexul, statusul marital, conditiile de locuit, educatie, statusul de sanatate si stilul de viata. Pacientii vor fi distribuiti in *grup de control si grup de interventie*, in scopul ingrijirii geriatrice secundare (Ambulatoriu) si tertiare (Spital). Pacientii din grupul de interventie vor primi consiliere geriatrica specifica lunara pe baza raportului computerizat generat in urma raspunsurilor la chestionarul HRA-O, pe o perioada de 6 luni. Consilierile vor fi realizate de investigatori/medici din Ambulatoriu si Spital. Dupa 6 luni de urmarire, evaluarea va include completarea unei forme scurte de chestionar HRA-O si a unui chestionar de nutritie. Pacientii din grupul de control vor beneficia de ingrijiri uzuale, nu de consilieri specifice.

Toate datele rezultate vor fi stocate intr-o baza electronica de date si analizate statistic de catre partenerii din Universitatea de Geriatrie din Berna. Datele pacientilor vor fi confidentiale.

Pacientii nu vor efectua investigatii biologice sau imagistice suplimentare, nu vor primi nici o substanta sau medicatie noua legata de acest studiu, iar tratamentele medicale curente si legatura acestora cu medicul curant nu vor fi afectate de studiu.

28.01.2014

Presedintele Comisiei de etica cercetarii,
Dr. Lupeanu Elena, CS II

Lupeanu
