# Supplementary material for: Effects of health risk assessment and counselling on physical activity in older people: A pragmatic randomised trial
Source: PLoS One. 2017 Jul 20;12(7):e0181371. doi: 10.1371/journal.pone.0181371 (PMC5519086; doi:10.1371/journal.pone.0181371)
Supplement: S2 Text — (PDF) [file pone.0181371.s005.pdf]

## Supporting Information for: Effects of health risk assessment and counselling on physical activity in older people: a pragmatic randomised trial

## Study protocol submitted to the Ethics Committee for the conduct and analysis of the trial that the ethics committee approved before the trial began

-ProjectPlan

-Annex.I.Flowchart

-Annex.II.Recruitment Form

-Annex.III.Brief Questionnaire EN

-Annex.III.Brief Questionnaire RO

-Annex.IV.Informed Consent EN

-Annex.IV.Informed Consent RO

-Annex.V.Baseline Questionnaire EN

-Annex.V.Baseline Questionnaire RO

-Annex.VI.Abstraction Health Record

-Annex.VII.Documentation of Special Events

-Annex.VIII.Final Questionnaire EN

-Annex.VIII.Final Questionnaire RO

-Annex.IX.Feedback Questionnaire EN

-Annex.X.Intervention Documentation Form

Medical Risk Assessment and Health Education
in Older People – RAHEO Study

A. Project Description

**Objectives:**

- evaluate feasibility and acceptability of the HRA-O cultural adapted questionnaire in Romania
- evaluate performance of HRA-O in secondary and tertiary specialized geriatric care settings in Romania

**Outcomes:**

- feasibility evaluation by analyzing completion rates, acceptability and feed back
- performance field testing by analyzing effects of specialized geriatric counseling, based on HRA-O generated report, in patients admitted to inpatient tertiary geriatric care and ambulatory secondary geriatric care (prevalence rates of identified health risk factors, co morbidities, health status and health behavior, self-perceived health status, participants' intention to change health behavior, prior and after specialized geriatric counseling).

**Type of study:**

Randomized Controlled Clinical Trial

**Methodology:**

► **Cultural adaptation and translation**

• A backward and forward translation method will be used for Brief Questionnaire (Annex III), Informed Consent (Annex IV), Health Risk Appraisal – Older People (HRA-O) instruments (Annex V, VIII) and Feed-back Questionnaire (Annex IX).

• Cultural adaptation will be done by discussing each item in researchers meetings and workshops.

• Simple translation processes will be applied for software items, and Ethics Committee approval.

• Recruitment Form (Annex II), Abstraction Health Record (Annex VI), Documentation of Special Events (Annex VII) and Intervention Documentation Form (Annex X) will remain in English language only.

► **Randomized Clinical Trial**

All activities of the Clinical Trial will take place in the sequence described in the Flowchart (Annex I).

■ **Subject selection and randomization**

Subject selection based on:

***Inclusion Criteria:*** - 65 years old and over

***Exclusion Criteria:***

- moderate to severe dementia: - equivalent to a MMSE score < 20
- severe disability : - needs human help in one or more basic activities of daily living
- has terminal illness
- has had major surgery within the last 3 months
- does not live in catchment area : - Bucharest and within 2 to 4 hours travel time
- lives in nursing home
- does not speak/understand Romanian language
- inability or unwillingness to fully complete Brief Questionnaire/Severe disability based on self-report
- person unwilling to give informed consent
- inability or unwillingness to complete HRA-O baseline questionnaire (at least all items included in the follow-up HRA-O questionnaire).

***Number of study subjects:***

Number of patients aged 65 and over admitted to inpatient geriatric care to be screened for this study: 300. Number of patients aged 65 and over referred to ambulatory geriatric care to be screened for this study: 300. Total number of patients aged 65 and over to be screened for this study is 600. Based on inclusion and exclusion criteria, a number of 200 patients in hospital setting and 200 patients in the ambulatory setting will be included in this study.

• All consecutive patients, aged 65 years and older (exact, based on date of birth) are evaluated for inclusion. Exclusion criteria are checked with the Recruitment Form (Annex II) checklist.

• Persons meeting inclusion criteria receive the Brief Questionnaire (Annex III). The Brief Questionnaire is checked. If incomplete, the patient is asked to complete all questions. If patient is unable/unwilling to fully complete the questionnaire, the patient is excluded. Also, the questionnaire is checked for the exclusion criterion of ADL disability.

• Persons meeting inclusion criteria are asked for informed consent.

Patients not giving informed consent are excluded.

• Persons receive a full base-line HRA-O questionnaire (Annex V) and are asked to complete the questionnaire (if needed with the help of a private proxy who is present).

The HRA-O is checked. If incomplete, the patient is asked to complete all questions. If patient is unable/unwilling to complete the questionnaire (at least all items included in the follow-up HRA-O questionnaire), the patient is excluded.

If patient completes HRA-O questionnaire (at least all items included in the follow-up HRA-O questionnaire), patient is included in study. No exclusion after this point.

Patient receives envelope with study number.

The sealed envelope with the corresponding study number is opened. (The sealed envelopes are provided by the statistical study center in Bern, based on a 1:1 randomization ratio and send to Bucharest 2 weeks prior to study initiation).

- After the person completes the HRA-O questionnaire, investigator will fill in the Abstraction Health Record Form (Annex VI) during a short (5 to 10 minutes interview with the subject). Record of medication intake will be based on bag with medications and/or medical written prescription.

- All persons' name, gender, data of birth, study number, date of future follow-up appointments and contact information will be recorded in a data base (Romanian).

• According to group allocation, older persons get the necessary information. Patients in Intervention Group will receive appointment for 1$^{st}$ counseling session within the next 4 to 8 working days after randomization. Patients in Control Group will receive usual care and appointment for follow-up after 6 months/ 6 months +2 weeks.

***Data collection at baseline (intervention and control groups):***

The following data will be collected at baseline:

- Recruitment Form (Annex II)
- Brief Questionnaire (Annex III)
- Informed Consent (Annex IV)
- Baseline HRA-O questionnaire (Annex V)
- Abstraction Health Record (Annex VI)
- Name, gender, date of birth, study number, date of future follow-up appointments, contact information – Romanian data base

***Data exchange:***

Annex II, III, IV, V, VI in both groups (Intervention and Control) will be scanned and sent to data base on a weekly basis on a Friday (Bucharest → Bern)

Participant HRA-O report will be sent to invetigators within 2 working days after having received scanned documents mentioned above (Bern → Bucharest).

■ **Intervention**

***Principle***:

Subjects in Intervention group will receive specialized geriatric counseling based on the HRA-O report. Subjects in Control group will receive usual care.

- personal feed-back report for participant, as generated by HRA-O system
- personal individual health counseling and preventive follow-up by a geriatrician in monthly intervals over a 6 month period
- the professional principles behind the intervention are given in the guidance notes
- the main objective is to improve the outcomes listed in the outcome section

***First counseling:***

Personal office visit in the clinic or in the hospital, duration 30 minutes, 4 to 8 working days after randomization, including:

- geriatrician explains and hands over personal feed-back report to older person
- geriatrician follows a checklist for all domains, and defines concrete and realistic goals/ recommendations for the patient until the next counseling session
- if needed, the geriatrician organizes interventions as needed (e.g. hearing check, vision check, additional work up of hypertension etc)
- the geriatrician summarizes main recommendations for the following month in print on a recommendation prescription
- at the end of the counseling session, the geriatrician defines the format and concrete date and time of the subsequent visit within one month: on site counseling of 15 minutes or on site counseling for 30 minutes

***Consecutive counseling:***

- 1 month/ 2 month/ 3 month/ 4 month/ 5 month follow-up ± 14 days
- same principle as above
- printed recommendation prescription is renewed as needed
- at each visit, next visit is scheduled

***Final counseling:***

- 6 calendar months ± 14 days after randomization
- same principle as above

- this session will have an additional 10 minutes duration when geriatrician will offer final reinforcement and final recommendation prescription, telling the older person what recommendations are important for continued implementation in the future.

Participants who do not participate to scheduled appointments for intervention will be contacted by telephone or mail using the contact details provided. Participants who do not receive counselling are the following cases: a) death, b) withdraw informed consent c) lost to follow-up as no contact is possible or d) living outside catchment area or not present for counselling for various personal reasons not related to study design.

***Design of usual care in the control group***

Persons allocated to the control group do receive usual care only. After randomization they get the information that they are invited for a six month follow-up.

***Data collection during intervention (intervention group):***

The following data will be collected during intervention:

- Documentation of special events (Annex VII)
- Intervention Documentation Form (Annex X)
- Reccomendation Prescription Form
-Abstraction Health Record (Annex VI)

***Data exchange:***

Annex VII will be scanned and sent to data base on a weekly basis on a Friday (Bucharest → Bern).

Annex VI, X and Recommendation Prescription Form will be filed in Romania for possible future analysis and Follow-up.

**■ 6 months Follow-up Evaluation**

All subjects in the intervention group will receive the final counseling session.

All subjects (intervention and control groups) will receive the short version of the HRA-O questionnaire (Follow-up Questionnaire - Annex VIII) for self completion.

All subjects in intervention group will receive the Feedback Questionnaire (Annex IX) for self completion.

***Outcome data collected with self-report questionnaire (extract of HRA-O questionnaire – Annex VIII):***

- physical activity
- low fat nutrition
- high fruit fiber nutrition

- alcohol use
- tobacco use
- medication self-management
- hearing screening
- eyesight screening
- fear of falling
- social network
- social activity
- depressive symptoms

***Information collected by objective evaluations:***

- blood pressure value
- information on antihypertensive medication (name of med, dose)
- information on anti-diabetic medication (name of med, dose)
- information on anti-cholesterol medication (name of med, dose)
- body weight
- influenza vaccination status (only if intervention is in vaccination season)

***Data collection at 6 months follow-up (intervention and control groups):***

The following data will be collected at 6 months follow-up:

- Documentation of special events (Annex VII)- Intervention Group
- Intervention Documentation Form (Annex X)- Intervention Group
- Final Reccomendation Prescription Form- Intervention Group
- Abstraction Health Record (Annex VI)- Intervention and Control Group
- Follow-up HRA-O short version Questionnaire (Annex VIII)- Intervention and Control Group
- Feedback Questionnaire (Annex IX)- Intervention Group

***Data exchange:***

Annex VI,VII, VIII, IX and X will be scanned and sent to data base on a weekly basis on a Friday (Bucharest → Bern). Final recommendation Prescription Form will be filed in Romania for possible future analysis.

**■ Statistical Analysis**

Database completion and Statistical analysis will be performed by partners at InselSpital, Geriatrics University, Bern, Switzerland. Results will be discussed between partners prior to conclusion and development of the publication draft.

Managers will ensure fluid communication and data transmission during the entire study span. Managers will monitor research activities, progress and expenses. The managers will supervise that work is progressing according to research plan and milestones (see GanttChart and Financial Plan).

B. Tasks and Work-packages

**Tasks:**

1. Project Management
   - Project Plan and Design
   - Organize Tasks, Activities and Work-packages
   - Develop short version of HRA-O as outcome instrument
   - Develop Acceptability and Feedback Questionnaires
   - Instruct research teams
   - Prepare publication draft
   - Workshops, Team Meetings, Dissemination
2. Development of the Romanian version of the instruments
   - Back to back translations/Simple translations
   - Cultural Adaptation
   - Workshops, Team Meetings
3. Intervention Study
   - Obtain Ethics Committee approval
   - Select subjects
   - Obtain signed informed consent
   - Randomize subjects
   - Instruct subjects to complete the questionnaires
   - Baseline evaluation
   - Software generated HRA-O reports
   - Intervention
   - Follow-up evaluation
   - Collect and send data to data base
4. Statistical analysis, results discussion and formulation of study conclusions
   - Data collection and statistical analysis
   - Analyse and discuss results for conclusions
   - Prepare draft article for publication

**Partners:**

Partner 1. - Research Institution

National Institute of Gerontology and Geriatrics "Ana Aslan", Bucharest – ROMANIA

Main Tasks: - Project Plan and Design

- Project Management
- Development of the Romanian version of the HRA-O instrument
- Intervention Study
- Dissemination

Secondary Tasks: - Organize Team Meetings, Workshops

- Participate to partners meetings
- Prepare publication draft

Partner 2. – Research Institution – InselSpital, Geriatrics University, Bern, SWITZERLAND

Main tasks: - Project Plan and Design

- Project Management
- Instructions for Field Test
- Send Software generated HRA-O reports
- Data collection and statistical analysis
- Analyse and discuss results for conclusions
- Prepare publication

Secondary Tasks: - Organize Team Meetings, Workshops

- Participate to partners meetings

**Individual Work-packages**

| **WP number:** | **1** | WP duration: | M1-M17 |
|---|---|---|---|
| WP title | **Project Management** | | |
| Activity type | Management, Organisation, Coordination, Monitoring, Communication | | |
| Participant no. | 1 | 2 | |
| Participant short name | NIGG<br>Ana Aslan | | |
| Name of<br>Person Responsible | Gabriel Ioan Prada | | |
| Person-months per participant | 13 | | |
| Objectives of the WP | | | |

| |
|---|
| - ensure that the project meets its objectives within specified budget, following the methodology and scheduled timetable |
| Description of work and role of partners<br>Task: - project initiation<br>- plan and organize activities<br>- instruct team<br>- organize and participate to meetings and workshops<br>- ensure fluid and rapid communication with partner<br>- monitor project progress<br>- monitor expenses<br>- track deliverables<br>- responsible for final report and publication draft<br>Partner 1: The manager will initiate the project and will organize team meeting for setting: role of team members, responsibilities, level of effort, milestones, and budget. The project manager will be responsible for ensuring the team members are being correctly instructed about the project methodology and timetable. He will monitor research activities, progress and expenses. The manager will supervise that work is progressing according to research plan and milestones. The manager is the designated person for communication with partner. The manager will maintain the project plan and provide revisions when necessary after consulting with partner. The manager will provide project reports to partner according to schedule. The manager will organize technical team meetings and workshops and also meetings with partner. The project manager will be responsible for scheduling and organising meetings, for producing agendas and minutes and coordinating discussions. The manager will also be responsible for documents management. When project manager is not available, all tasks are redirected to second researcher.<br>Partner 2: The project manager will be responsible for ensuring the team members are being correctly instructed about the project methodology and timetable. The manager will supervise that work is progressing according to research plan and milestones. The manager will supervise that data base is being completed, reports are being generated and statistical analysis is being done according to schedule. The manager is the designated person for communication with partner. The manager will maintain the project plan and provide revisions when necessary after consulting with partner. The manager will provide project reports to partner according to schedule. The manager will organize meetings with partner. |
| Deliverables of the WP: |

| |
|---|
| Month 3: Partner 1: Report on Cultural Adaptation and Translation Process/Report on Ethics Committee Approval/Initiate Intervention Study<br>Month 7: Partner 1: Report on Recruitment Process, Baseline Evaluation and Intervention<br>Month 10: Partner 1: Report on Intervention<br>Month 13: Report on Follow-up Evaluation/End of Study<br>Month 15: Partner 2: Report on Statistical Analysis and Results<br>Month 17: Partner 1: Final Project Report<br>Month 17: Partner 2: Final Project Report |

| **WP number:** | **2** | WP duration: | M1-M17 |
|---|---|---|---|
| WP title | **Intervention Study** | | |
| Activity type | Research and Development | | |
| Participant no. | 1 | 2 | |
| Participant short name | NIGG<br>Ana Aslan | | |
| Person-months per participant | 24 | | |
| Objectives of the WP<br>- evaluate feasibility and acceptability of the HRA-O cultural adapted questionnaire in Romania<br>- evaluate performance of HRA-O in secondary and tertiary specialized geriatric care settings in Romania | | | |
| Description of work and role of partners<br>Task: - develop short version of HRA-O as outcome instrument<br>- develop Acceptability and Feedback Questionnaires<br>- development of the Romanian version of the instruments<br>- obtain ethics approval<br>- obtain informed consent<br>- instruct subjects to complete questionnaires<br>- recruit and randomize subjects<br>- software generated HRA-O reports<br>- specialized geriatric counselling based on HRA-O reports/usual care<br>- collect and send data to database<br>- data collection and statistical analysis | | | |

| |
|---|
| - analyse results/ further changes or modifications/conclusions<br>- prepare publication draft<br>Partner 1: Conduct intervention study according to methodology and timetable. Send data to data base. Team meeting and discussion with partner about results and conclusions. Promote project at national conferences and congresses (oral presentations/posters). Prepare publication draft.<br>Partner 2: Collect data into database. Generate HRA-O reports and communicate to partner according to the timetable. Conduct statistical analysis. Discuss results and conclusion with partner. Promote project and prepare publication draft. |
| Deliverables of the WP:<br>Month 3: Partner 1: Complete Cultural Adaptation and Translation Process<br>Month 5: Partner 2: Complete Database update<br>Month 5: Partner 1: Initiate Intervention Study<br>Month 7: Partner 1: Finalize Recruitment Process<br>Month 13: Partner 1: Finalize Intervention and Follow-up/End of Study<br>Month 14: Partner 2: Statistical Analysis/Study Results generation<br>Month 15: Partner 2: Report results/Discuss results with partner/Conclusions<br>Month 15: Partner 1: Discuss results with partner/Conclusions<br>Month 17: Partner 1: Publication Draft<br>Month 17: Partner 2: Publication Draft |

Participant 1 – Romania – National Institute of Geriatrics and Gerontology “Ana Aslan”, Bucharest
Researcher 1.1 – Assoc. Prof. Gabriel Ioan Prada MD, PhD
In-patient Department
Researcher 1.2 – Anna Marie Herghelegiu MD, PhD
Out-patient Department

Participant 2 – Switzerland – InselSpital, Geriatrics University, Bern
Professor Andreas Stuck
Stephan Born

**Annex I**

## HRA-O Study in Romania: Flow-Chart part I

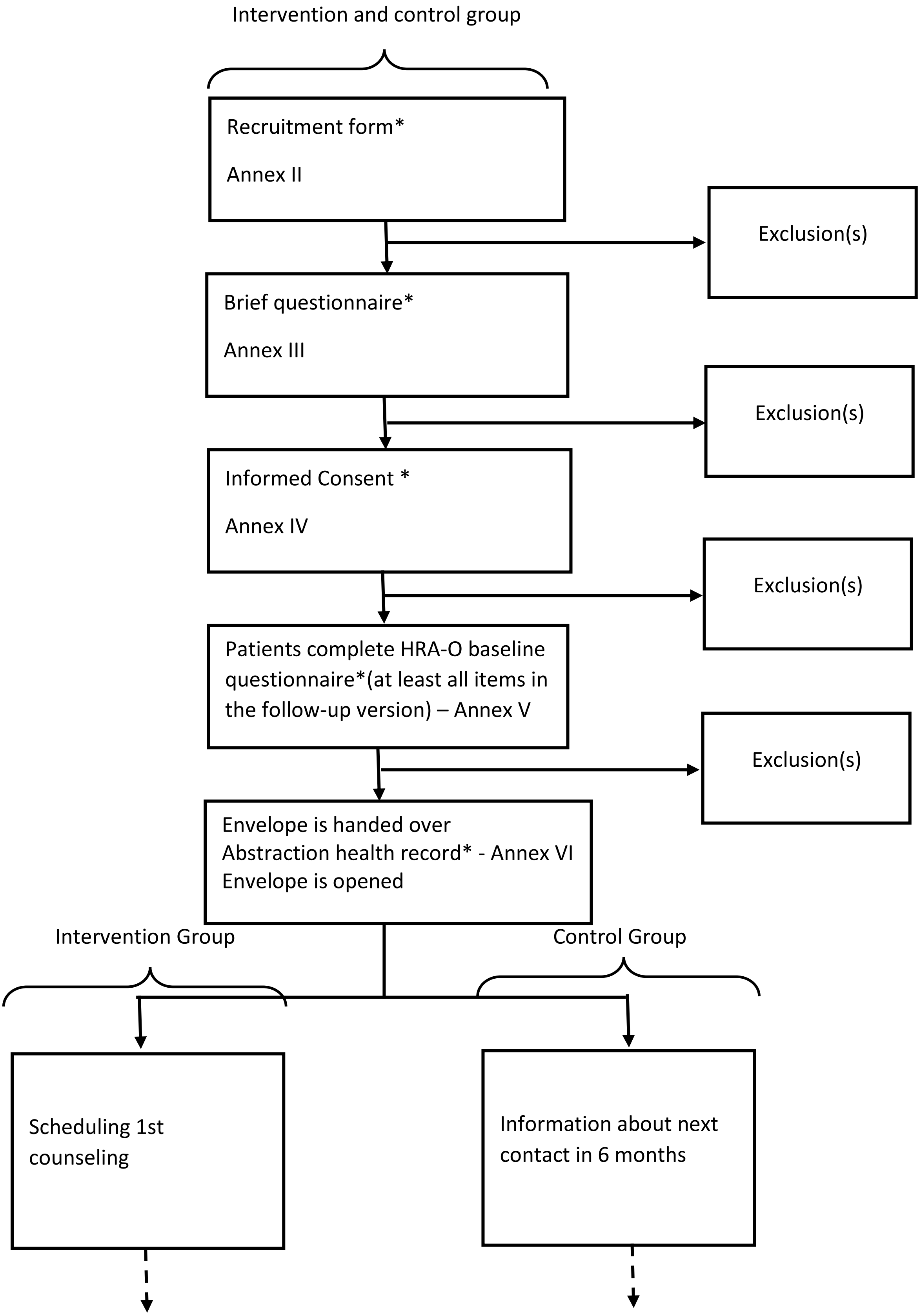


* Documents to be uploaded to Bern

**HRA-O Study in Romania: Flow-Chart part II**

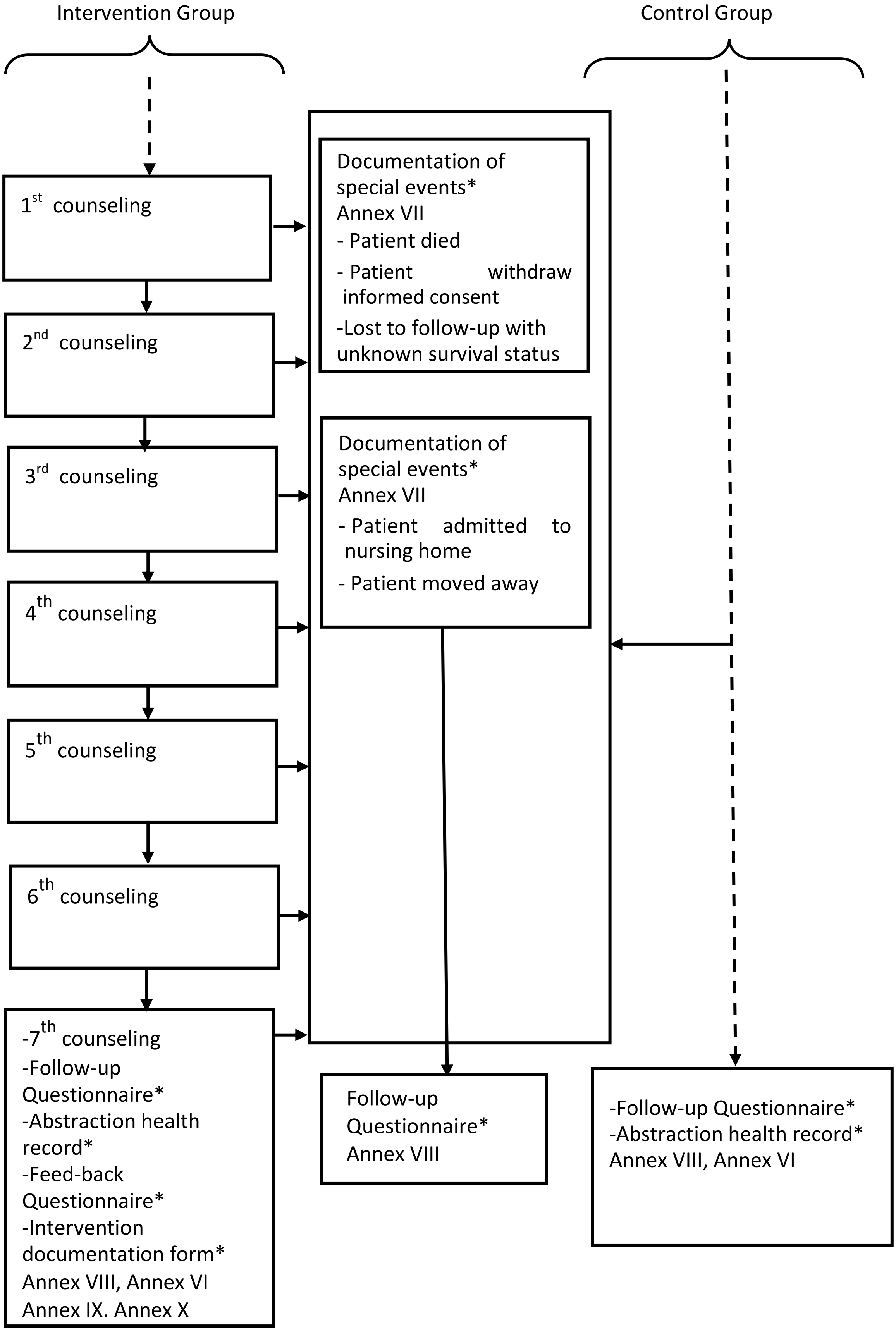


* Documents to be uploaded to Bern

## Annex II

## Recruitment form

*Please complete one form for each consecutively admitted person aged 65 years and older*

| | |
|---|---|
| Date of recruitment (DD/MM/YY) | |
| Location of recruitment | ☐1 Hospital ☐2Out-patient |
| First name | |
| Surname | |
| Date of birth (DD/MM/YY) | |
| Gender | ☐1 female ☐2 male |

*Please check for the following exclusion criteria in the order listed. If one of the answers is "Yes", stop with checking the criteria and exclude the patient.*

| | | |
|---|---|---|
| No check done for logistical reason (no capacity for recruitment of patients) *it is planned that this will not occur, but if it occurs it should be recorded* | ☐1 Yes | ☐2 No |
| Does not live in catchment area:<br><br>Bucharest and within 2 to 4 hours travel time | ☐1 Yes | ☐2 No |
| Is already included in the study | ☐1 Yes | ☐2 No |
| Has already been excluded from the study at an earlier admission | ☐1 Yes | ☐2 No |
| Lives in nursing home | ☐1 Yes | ☐2 No |
| Does not speak/ understand Romanian language | ☐1 Yes | ☐2 No |
| Has moderate to severe dementia:<br><br>equivalent to a MMSE score< 20 | ☐1 Yes | ☐2 No |
| Has severe disability<br><br>Needs human help in one or more basic activities of daily living | ☐1 Yes | ☐2 No |
| Has terminal illness | ☐1 Yes | ☐2 No |

| | | |
|---|---|---|
| Had major surgery within last 3 months | ☐1 Yes | ☐2 No |

If all items are answered with NO, continue with brief questionnaire

| | | |
|---|---|---|
| Inability or unwillingness to fully complete brief questionnaire | ☐1 Yes | ☐2 No |
| Severe disability based on self-report | ☐1 Yes | ☐2 No |

If all items are answered with NO, continue with informed consent

| | | |
|---|---|---|
| Person unwilling to give informed consent. | ☐1 Yes | ☐2 No |

If person gives informed consent continue with HRA-O baseline questionnaire.

| | | |
|---|---|---|
| Inability or unwillingness to fully complete HRA-O baseline questionnaire | ☐1 Yes | ☐2 No |

If person completes at least all items in the follow-up version of the HRA-O questionnaire, person is included in the study.

**Annex III**

# Brief Questionnaire Romania

| Name or stamp of Geriatrician: |
|---|
| |

| | |
|---|---|
| Location | ☐1 Hospital ☐2 Out patient |
| First name | |
| Surname | |
| Date of Birth (DD/MM/YY) | |
| Date of examination | |

*Please complete this Brief Questionnaire.*

*Please do the following:*
- *Read each question carefully and follow the instructions.*
- *Put a cross in the relevant box for each answer, like this:* ☒
- *Do not write any comments in blank spaces or in the margins, they will not be considered.*
- *Answer the questions in each section even if the topic does not apply to you directly.*
- *Please check at the end that you have completed every page and every question on each page.*

© 2013 This questionnaire is protected by copyright

# Your Health Measurements

1. All in all, would you say that your health is generally …
   - ☐1 ... Excellent
   - ☐2 ... Good
   - ☐3 ... Fair
   - ☐4 ... Poor

2. What is your height (without shoes)?

   ☐ m ☐☐ cm

3a. Has your height decreased as you have become older?
   - ☐1 No → *Skip to question 4 on this page*
   - ☐2 Yes ↓

3b. What was your height when you were a young adult?

   ☐ feet ☐☐ inches or ☐ m ☐☐ cm

4. What is your weight (wearing light clothing without shoes)?

   ☐☐☐ kg

5. Have you lost weight <u>in the last three months</u>?
   - ☐1 Yes, I have lost more than 3 kilos (7 pounds)
   - ☐2 Yes, I have lost between 1 and 3 kilos (2 to 6 pounds)
   - ☐3 No, I have not lost weight

6. What is your blood pressure?

   ☐☐☐ Top number (Systolic) ☐☐☐ Bottom number (Diastolic)

   - ☐1 Don't know/not sure

7. <u>In the last three months</u> have you had a sudden illness, an operation or experienced psychological stress?
   - ☐1 No
   - ☐2 Yes

© 2013 This questionnaire is protected by copyright

# Medical History

| Has a doctor <u>ever</u> told you that you have any of the following:<br>***Please answer each question with No or Yes.*** | | **No** | **Yes** |
|---|---|---|---|
| 1. | High blood pressure | ☐1 | ☐2 |
| 2. | High cholesterol | ☐1 | ☐2 |
| 3. | Diabetes | ☐1 | ☐2 |
| 4. | Anaemia | ☐1 | ☐2 |
| 5. | Coronary heart disease or heart attack | ☐1 | ☐2 |
| 6. | Heart failure | ☐1 | ☐2 |
| 7. | Stroke | ☐1 | ☐2 |
| 8. | Venous thrombosis (in either surface or deep veins) or varicose veins | ☐1 | ☐2 |
| 9. | Chronic bronchitis or emphysema | ☐1 | ☐2 |
| 10. | Asthma | ☐1 | ☐2 |
| 11. | Osteoporosis | ☐1 | ☐2 |
| 12. | Arthritis or rheumatism | ☐1 | ☐2 |
| 13. | Depression | ☐1 | ☐2 |
| 14. | Dementia (including Alzheimer's disease) | ☐1 | ☐2 |
| 15. | Glaucoma | ☐1 | ☐2 |
| 16. | Cancer of any kind | ☐1 | ☐2 |
| 17. | Stomach or duodenal ulcers | ☐1 | ☐2 |
| 18. | Liver disease of any kind | ☐1 | ☐2 |
| 19. | Kidney disease | ☐1 | ☐2 |

© 2013 This questionnaire is protected by copyright

# Functioning

*The following questions are about basic activities of daily living.* ***Please say if you have difficulties with any of them, or need the help of another person to do them.***

| Do you manage the following activities on your own or with help from others? | | Yes, without difficulty | Yes, with diffi-culty | Yes, with help from someone |
|---|---|---|---|---|
| 1. | Feed yourself | ☐1 | ☐2 | ☐3 |
| 2. | Get yourself to the toilet | ☐1 | ☐2 | ☐3 |
| 3. | Dress yourself | ☐1 | ☐2 | ☐3 |
| 4. | Bath or shower yourself | ☐1 | ☐2 | ☐3 |
| 5. | Move from your bed to a chair or to standing up | ☐1 | ☐2 | ☐3 |

## Thank you for completing this questionnaire!

© 2013 Geriatric Research Fund, Spital Netz Bern AG, Inselspital, PO Box 20, CH-3010 Bern, Switzerland. This questionnaire is protected by copyright and may not (as a whole or in part) be reproduced or used for other purposes without prior written permission of the copyright owners.

# Scurt Chestionar despre Starea de Sănătate

*Va rugăm să nu completați acest tabel.*

ID-number ☐☐☐☐☐☐☐☐☐☐☐☐☐☐☐☐☐☐

Study Group ☐ 1 **A** Intervention ☐ 2 **B** Placebo

Location ☐ 1 Hospital ☐ 2 Out patient

First name ☐☐☐☐☐☐☐☐☐☐☐☐☐☐☐☐☐☐

Surname ☐☐☐☐☐☐☐☐☐☐☐☐☐☐☐☐☐☐

Date of examination Day ☐☐ Month ☐☐ Year ☐☐☐☐

*Vă rugăm să completați acest chestionar*

*Vă rugăm să:*

- *Citiți fiecare întrebare cu atenție și urmăriți instrucțiunile.*
- *Puneți un X în pătratul relevant pentru fiecare întrebare astfel:* ☒
- *Nu scrieți comentarii în spațiile libere sau pe margini, nu vor fi luate în considerare.*
- *Răspundeți întrebărilor de la fiecare capitol, chiar dacă subiectul respectiv nu se aplică în cazul dvs.*
- *La sfârșit verificați dacă ați completat fiecare pagină și dacă ați răspuns la toate întrebările de pe fiecare pagină.*

© 2014 Acest chestionar este protejat prin legea dreptului de autor 

# Date despre sănătatea dvs.

1. În general ați spune că starea sănătății dvs. este .....

☐ 1 Excelentă ☐ 2 Bună
☐ 3 Satisfăcătoare ☐ 4 Proastă

2. Care este înălțimea dvs. *(fără încălțăminte)*?

☐ m ☐☐ cm

3a. Ați scăzut în înălțime pe măsură ce ați înaintat în vârstă?

☐ 1 Nu *Mergeți la întrebarea 4 pe această pagină*
☐ 2 Da

3b. Care a fost înălțimea dvs. în tinerețe ?

☐ m ☐☐ cm

4. Care este greutatea dvs. corporală *(purtând haine ușoare și fără încălțăminte)*?

☐☐☐ kg

5. Ați pierdut în greutate <u>în ultimele 3 luni</u> ?

☐ 1 Da, am slăbit mai mult de 3 kilograme
☐ 2 Da, am slăbit 1- 3 kilograme
☐ 3 Nu, nu am slăbit

6. Care sunt valorile tensiunii dvs. arteriale ?

☐☐☐ Valoarea de sus (sistolică) ☐☐☐ Valoarea de jos (diastolică)
☐ 1 Nu știu / nu sunt sigur/ă

7. În ultimele 3 luni ați suferit de vreo afecțiune acută, o operație sau un stres emoțional?

☐ 1 Nu ☐ 2 Da

© 2014 Acest chestionar este protejat prin legea dreptului de autor

# Istoricul dvs. medical

| | V-a spus <u>vreodată</u> un medic că aveți vreuna din următoarele afecțiuni :<br>*Vă rugăm răspundeți la fiecare întrebare cu Da sau Nu.* | Nu | Da |
|---|---|---|---|
| 1. | Tensiune arterială crescută *(hipertensiune)* | ☐ 1 | ☐ 2 |
| 2. | Colesterol crescut *(hipercolesterolemie)* | ☐ 1 | ☐ 2 |
| 3. | Diabet zaharat | ☐ 1 | ☐ 2 |
| 4. | Anemie | ☐ 1 | ☐ 2 |
| 5. | Boala arterelor inimii *(cardiopatie ischemică)* sau atac de cord | ☐ 1 | ☐ 2 |
| 6. | Insuficiență cardiacă | ☐ 1 | ☐ 2 |
| 7. | Accident vascular cerebral | ☐ 1 | ☐ 2 |
| 8. | Tromboză venoasă *(la nivelul venelor superficiale sau pro-funde)* sau varice | ☐ 1 | ☐ 2 |
| 9. | Bronșită cronică sau emfizem | ☐ 1 | ☐ 2 |
| 10. | Astm bronșic | ☐ 1 | ☐ 2 |
| 11. | Osteoporoză | ☐ 1 | ☐ 2 |
| 12. | Artroză sau reumatism | ☐ 1 | ☐ 2 |
| 13. | Depresie | ☐ 1 | ☐ 2 |
| 14. | Demență *(inclusiv boala Alzheimer)* | ☐ 1 | ☐ 2 |
| 15. | Glaucom | ☐ 1 | ☐ 2 |
| 16. | Cancer de orice fel | ☐ 1 | ☐ 2 |
| 17. | Ulcer gastric sau duodenal *(stomac sau duoden)* | ☐ 1 | ☐ 2 |
| 18. | Afecțiune hepatică *(boală a ficatului)* de orice fel | ☐ 1 | ☐ 2 |
| 19. | Afecțiune renală *(boală a rinichilor)* | ☐ 1 | ☐ 2 |

© 2014 Acest chestionar este protejat prin legea dreptului de autor

# Funcționare

*Următoarele întrebări se referă la activitățile obișnite de zi cu zi.* ***Vă rugăm să ne spuneți dacă aveți dificultăți cu oricare dintre ele sau aveți nevoie de ajutorul cuiva pentru a le face.***

| **Puteți desfășura următoarele activități singur/ă sau cu ajutorul cuiva ?** | | **Da, fără dificultate** | **Da, cu dificultate** | **Da, cu ajutorul cuiva** |
|---|---|---|---|---|
| 1. | Să vă hrăniți | ☐ 1 | ☐ 2 | ☐ 3 |
| 2. | Să mergeți la toaletă | ☐ 1 | ☐ 2 | ☐ 3 |
| 3. | Să vă îmbrăcați | ☐ 1 | ☐ 2 | ☐ 3 |
| 4. | Să vă spălați / să faceți baie sau duș | ☐ 1 | ☐ 2 | ☐ 3 |
| 5. | Să vă mutați din pat pe un scaun sau să vă ridicați | ☐ 1 | ☐ 2 | ☐ 3 |

## Vă mulțumim pentru că ați completat acest chestionar!

© 2014 Geriatric Research Fund, Spital Netz Bern AG, Inselspital, PO Box 20, CH-3010 Bern, Switzerland. Acest chestionar este protejat prin legea dreptului de autor și nu poate fi (parțial sau total) reprodus sau utilizat în alte scopuri fără aprobarea prealabilă scrisă a deținătorilor dreptului de autor.

**Annex IV**

**Informed Consent for Participating in Study**

"Medical Risk Assessment and Health Education
in Older People – RAHEO Study"

This research project will take place at the National Institute of Gerontology and Geriatrics "Ana Aslan", Bucharest during a 12 months period starting March 2014. It is developed and coordinated in cooperation with Geriatrics University of Berne, Switzerland.

The main objective of this study is to evaluate health risks of people 65 years of age and older, and to offer specific medical counseling in order to correct unhealthy behavior.

There will be 400 patients included, after meeting inclusion and exclusion criteria and after agreeing to be part of the study. All patients will be invited to answer questions regarding their name, contact details, age, sex, marital status, living arrangements, education, income, health status and lifestyle habits. A group of 200 patients will receive a computer generated report about their health status which will be discussed together with their doctor. Based on this report, specific counseling sessions tailored to patients' health and lifestyle needs in order to improve their general health status, will be offered by their doctor by appointment, on a monthly basis. After 6 months, patients will be asked to answer questions about their lifestyle habits and feed-back questions about their experience being part of this study. Another group of 200 patients will receive usual medical care without specific counseling. After 6 months, these patients will be asked to answer questions about their lifestyle habits. Assignment to these 2 study groups will be done randomly. All data will be stored into an electronic database for analysis. No personal information will be disclosed to other parties. Your participation in this study will be kept confidential. You can withdraw from the study, without any consequence, at any time, whether before it starts or while you are participating. This will not alter your status, nor your relationship with your doctor.

If you agree to take part in the study, please sign the consent form overleaf.

**Consent Form**

I, ……………………………………………………………………………………………… agree to participate in this research study.

The purpose and nature of the study has been explained to me in writing as well as verbally, and I completely understood all the terms of my participation.

I am participating voluntarily.

I give permission that all my personal and medical data as well as my answers to study questions to be stored into an electronic database and analyzed for the purpose of this study.

I understand that my personal information will not be disclosed to other parties and participation in this study will be kept confidential.

I understand that I can withdraw from the study, without any consequence, at any time, whether before it starts or while I am participating.

Signed……………………………………. Date……………….

**Consimţământ informat de participare în studiul**

"Evaluarea Riscului Medical şi Promovarea Sănătăţii
pentru persoanele vârstnice - studiul RAHEO"

Acest proiect de cercetare se va desfăşura la Institutul Naţional de Gerontologie şi Geriatrie "Ana Aslan" , Bucureşti, pe o perioadă de 12 luni, începând cu luna mai 2014 . Proiectul este realizat şi coordonat în cooperare cu Universitatea de Geriatrie din Berna, Elveţia.

Principalul obiectiv al acestui studiu este evaluarea riscului de sănătate pentru persoane în vârstă de cel puţin 65 ani în scopul de a le oferi consiliere medicală specifică care să corecteze obiceiuri de viaţă nesănătoase.

Vor fi incluşi un număr de 400 de pacienţi pe baza unor criterii de includere şi excludere şi după ce aceştia vor semna consimţământul informat de includere în studiu. Toţi pacienţii vor fi invitaţi să răspundă unor întrebări referitoare la numele lor, vârstă, sex, date de contact, stare civilă, condiţii în care locuiesc, educaţie, venituri, starea de sănătate şi obiceiuri de viaţă. Un grup de 200 de pacienţi vor primi un raport/rezumat al stării lor generale de sănătate care va fi discutat împreună cu medicul. În funcţie de acest raport, vor fi organizate consultaţii de consilere, lunare şi pe bază de programare, şedinţe în care medicul va oferi pacienţilor recomandări, informaţii şi sfaturi specifice, bazate pe nevoiele de sănătate ale acestora în scopul de a le îmbunătăţi starea de sănătate. După 6 luni pacienţii vor fi invitaţi să răspundă unor întrebări referitoare la modul lor de viaţă şi întrebări feedback despre experienţa lor de a face parte din acest studiu. Un alt grup de 200 de pacienţi vor primi îngrijiri medicale obişnuite, fără consultaţii de consiliere specifică. După 6 luni aceşti pacienţi vor fi invitaţi să răspundă unor întrebări referitoare la modul lor de viaţă. Distribuirea pacienţior în aceste 2 grupuri va fi făcută randomizat (întâmplător). Toate datele vor fi introduse într-o bază de date electronică pentru a fi analizate. Nici un fel de informaţie personală nu va fi transmisă altor părţi. Participarea dvs. în acest studiu va fi confidenţială. Vă puteţi retrage din studiu în orice moment, fără nici un fel de consecinţe. Acest lucru nu va afecta relaţia dvs. cu medicul.

Dacă sunteţi de acord să faceţi parte din acest studiu vă rugăm să semnaţi pe verso.

**Formular de consimţământ**

Subsemnatul/a ,
..........................................................................................................................................sunt de acord să particip în acest studiu.

Scopul şi natura studiului mi-au fost explicate atât în scris cât şi verbal şi declar că am înţeles pe deplin termenii participării mele în studiu.

Particip în acest studiu voluntar.

Acord permisiunea ca toate datele mele personale şi medicale, cât şi răspunsurile mele la întrebările din chestionare să fie introduse într-o bază de date electronică pentru a fi analizate în scopul de cercetare al acestui studiu.

Înteleg că nici una din datele mele personale nu va fi divulgată altor părţi şi că participarea mea în acest studiu este confidenţială.

Înţeleg că mă pot retrage din acest studiu în orice moment şi fără nici o consecinţă.

Semnătura............................................ Data.......................

**Annex V**

# Baseline Questionnaire Romania

| |
|---|
| *Name or stamp of Geriatrician* |

| | | |
|---|---|---|
| ID-number | | |
| Study Group | ☐1 A Intervention | ☐2B Placebo |
| Location | ☐1 Hospital | ☐2 Out patient |
| First name | | |
| Surname | | |
| Date of examination | | |

*Please complete this Baseline Questionnaire.*

*Please do the following:*

- *Read each question carefully and follow the instructions.*
- *Put a cross in the relevant box for each answer, like this:* ☒
- *Do not write any comments in blank spaces or in the margins, they will not be considered.*
- *Answer the questions in each section even if the topic does not apply to you directly.*
- *Please check at the end that you have completed every page and every question on each page.*

# Medications/Prescriptions

*The following questions are about medicines, including prescribed and over the counter medicines (including herbal remedies).* ***Please also include medicines that you take only occasionally.***

1. How many medicines or remedies have you taken by mouth in tablet, capsule or liquid form in the last 7 days? The last answer is '10 or more'?

| **0** | **1** | **2** | **3** | **4** | **5** | **6** | **7** | **8** | **9** | **more** |
|---|---|---|---|---|---|---|---|---|---|---|
| ☐ 0 | ☐ 1 | ☐ 2 | ☐ 3 | ☐ 4 | ☐ 5 | ☐ 6 | ☐ 7 | ☐ 8 | ☐ 9 | ☐ 10 |

2. In the last 7 days have you taken any other medicines or remedies, but **not** by mouth?

| | | | |
|---|---|---|---|
| a. | Eyedrops | ☐ 1 Yes | ☐ 2 No |
| b. | Skin patches (containing medication) | ☐ 1 Yes | ☐ 2 No |
| c. | Injections | ☐ 1 Yes | ☐ 2 No |
| d. | Inhalers | ☐ 1 Yes | ☐ 2 No |
| e. | Suppositories | ☐ 1 Yes | ☐ 2 No |

*Answer the following questions based on any* ***medications or remedies you have taken in any form*** *in the last 7 days.*

| **Have you taken any of the following medicines or remedies in the last 7 days?** | | **No** | **Yes** |
|---|---|---|---|
| 3. | Medicine for high blood pressure | ☐ 1 | ☐ 2 |
| 4. | Medicine for high cholesterol | ☐ 1 | ☐ 2 |
| 5. | Medicine for diabetes | ☐ 1 | ☐ 2 |
| 6. | Medicine for heart conditions | ☐ 1 | ☐ 2 |
| 7. | Anti-coagulants (for thinning the blood) like Warfarin or medicines to reduce platelet stickiness like Aspirin or Clopidogrel | ☐ 1 | ☐ 2 |
| 8. | Medicine to help you fall asleep | ☐ 1 | ☐ 2 |
| 9. | Painkillers | ☐ 1 | ☐ 2 |
| 10. | Medicine for anxiety (nerves or nervousness) | ☐ 1 | ☐ 2 |
| 11. | Medicine for depression | ☐ 1 | ☐ 2 |
| 12. | Anti-Alzheimer drugs | ☐ 1 | ☐ 2 |
| **(Women only)*:*** | | | |
| 13. | Medicine to replace female hormones | ☐ 1 | ☐ 2 |

14a. Are you allergic to any medicine?

☐1 No → *Skip to question 15 on this page*

☐2 Yes ↓

14b. Would you like to carry a 'medic-alert' or similar information about your medicine allergy?

☐1 No

☐2 Yes

*The following questions ask about problems that you may have in taking medicines.* ***If <u>at the moment</u> you DO NOT take any medicines, go to page 6.***

15. Do you have a medication plan that lists your current medicines?

☐1 No

☐2 Yes

16. Do you use a 'Dosette box' (organiser for your pills) or blister pack?

☐1 No

☐2 Yes

17. Do you have difficulties in understanding when and how you should take your medicines?

☐1 No

☐2 Yes

18. Are you uncertain why you are taking any of your current medicines?

☐1 No

☐2 Yes

19. Are you prescribed medicines by more than one doctor?

☐1 No

☐2 Yes

20. Do you think that you are having any side-effects due to your medications?

☐1 No

☐2 Yes

# Pain

*The following questions ask about any pain that you experience, how severe it is and if it interferes with everyday life. The first 2 questions ask about your experience of pain, based on a scale from zero to ten, with “**0**” meaning “no pain” and “**10**” meaning “the worst pain you can imagine”.*

1. How severe is your pain ***today***?

| 0 | 1 | 2 | 3 | 4 | 5 | 6 | 7 | 8 | 9 | 10 |
|---|---|---|---|---|---|---|---|---|---|---|
| ☐0 | ☐1 | ☐2 | ☐3 | ☐4 | ☐5 | ☐6 | ☐7 | ☐8 | ☐9 | ☐10 |

no pain worst pain

2. In the ***last 7 days***, how severe has your pain been *on* ***average***?

| 0 | 1 | 2 | 3 | 4 | 5 | 6 | 7 | 8 | 9 | 10 |
|---|---|---|---|---|---|---|---|---|---|---|
| ☐0 | ☐1 | ☐2 | ☐3 | ☐4 | ☐5 | ☐6 | ☐7 | ☐8 | ☐9 | ☐10 |

no pain worst pain

| **Because of pain, …** | | **No** | **Yes** |
|---|---|---|---|
| 3. | … have you cut down the amount of time you spend on work or other activities? | ☐1 | ☐2 |
| 4. | … have you been accomplishing less than you would like to? | ☐1 | ☐2 |
| 5. | … have you had to give up work or other activities? | ☐1 | ☐2 |
| 6. | … does the work or activities you do require extra effort? | ☐1 | ☐2 |
| 7. | … do you do things less often with friends or relatives? | ☐1 | ☐2 |
| 8. | … do you have trouble sleeping? | ☐1 | ☐2 |

*The next questions ask about pain that can occur with different activities. Choose the answer that comes closest to being accurate for you.*

| **Do you currently have pain with the following activities?** | | **No** | **Yes** |
|---|---|---|---|
| 9. | Walking 200 yards or less | ☐1 | ☐2 |
| 10. | Walking more than 200 yards | ☐1 | ☐2 |
| 11. | Climbing more than one flight of stairs | ☐1 | ☐2 |
| 12. | Moderate activities *such as moving a heavy table, pushing a vacuum cleaner, bowling, or playing golf* | ☐1 | ☐2 |

# Vision

*The following questions address problems which involve your eyesight or feelings that you have about your eyesight condition. After each question, please choose the response that best describes your situation.* ***If you wear spectacles or contact lenses, answer as if wearing them. Choose the answer that best describes your situation.***

1. At the present time, would you say your eyesight using both eyes (with your glasses *or contact lenses, if you wear them*) is:
   - ☐1 Excellent → *Skip to page 9 (Hearing)*
   - ☐2 Good
   - ☐3 Fair
   - ☐4 Poor
   - ☐5 Very Poor
   - ☐6 Completely Blind

*The next questions are about how much difficulty, if any, you have doing certain activities (wearing your glasses or contact lenses if you use them for that activity).*

2. How much difficulty do you have reading ordinary print in newspapers? Would you say you have:
   - ☐1 No difficulty at all
   - ☐2 A little difficulty
   - ☐3 Moderate difficulty
   - ☐4 Extreme difficulty
   - ☐5 Stopped doing this because of your eyesight
   - ☐6 Stopped doing this for other reasons or not interested in doing this

3. How much difficulty do you have doing work or hobbies that require close vision, *such as cooking, sewing, fixing things around the house, or using hand tools*? Would you say, you have:
   - ☐1 No difficulty at all
   - ☐2 A little difficulty
   - ☐3 Moderate difficulty
   - ☐4 Extreme difficulty
   - ☐5 Stopped doing this because of your eyesight
   - ☐6 Stopped doing this for other reasons or not interested in doing this

4. Because of your eyesight, how much difficulty do you have going down steps, stairs, or kerbs in dim light or at night?
   - ☐1 No difficulty at all
   - ☐2 A little difficulty
   - ☐3 Moderate difficulty
   - ☐4 Extreme difficulty
   - ☐5 Stopped doing this because of your eyesight
   - ☐6 Stopped doing this for other reasons

5. Because of your eyesight, how much difficulty do you have noticing objects off to the side while you are walking along?

- ☐1 No difficulty at all
- ☐2 A little difficulty
- ☐3 Moderate difficulty
- ☐4 Extreme difficulty
- ☐5 Stopped doing this because of your eyesight
- ☐6 Stopped doing this for other reasons

6. Because of your eyesight, how much difficulty do you have finding something on a crowded shelf up close to you?

- ☐1 No difficulty at all
- ☐2 A little difficulty
- ☐3 Moderate difficulty
- ☐4 Extreme difficulty
- ☐5 Stopped doing this because of your eyesight
- ☐6 Stopped doing this for other reasons

7. Are you limited in how long you can work or do other activities because of your eyesight?

- ☐1 None of the time
- ☐2 A little of the time
- ☐3 Some of the time
- ☐4 Most of the time
- ☐5 All of the time

*The next questions are about car driving.*

8a. Are you currently driving, at least once in a while?

- ☐1 Yes → *Skip to question 9 on this page*
- ☐2 No

8b. ***If No:*** Have you never driven a car, or have you given up driving?

- ☐1 Never drove → *Skip to page 9 (Hearing)*
- ☐2 Gave up ↓

8c. Why did you give up driving? Was that mainly because of your eyesight, mainly for some other reason, or because of both your eyesight and other reasons?

- ☐1 Mainly eyesight
- ☐2 Mainly other reasons
- ☐3 Both eyesight and other reasons

*The following question is for current drivers.*

9. How much difficulty do you have driving during the daytime in familiar places
Would you say you have:

- ☐1 No difficulty at all
- ☐2 A little difficulty
- ☐3 Moderate difficulty
- ☐4 Extreme difficulty

# Hearing

*These questions are about any difficulties that you have with hearing.* ***If you wear a hearing aid, answer as if you were using it.***

1. How would you rate your hearing *(with your hearing aid on, if applicable)*?
   - ☐1 Excellent
   - ☐2 Good
   - ☐3 Fair
   - ☐4 Poor
   - ☐5 Very poor
   - ☐6 Deaf

| **Does your hearing (with your hearing aid, if you wear one) cause you ...** | | **No** | **Some-times** | **Yes** |
|---|---|---|---|---|
| 2. | ... to feel embarrassed when you meet new people? | ☐1 | ☐2 | ☐3 |
| 3. | ... to feel frustrated when talking to members of your family? | ☐1 | ☐2 | ☐3 |
| 4. | ... to have difficulty when visiting friends, relatives, or neighbours? | ☐1 | ☐2 | ☐3 |
| 5. | ... to attend religious services or a play at the theatre less often than you would like? | ☐1 | ☐2 | ☐3 |
| 6. | ... to have arguments with family members? | ☐1 | ☐2 | ☐3 |
| 7. | ... to have difficulty when listening to radio or television? | ☐1 | ☐2 | ☐3 |
| 8. | ... to have difficulty when in a restaurant with relatives or friends? | ☐1 | ☐2 | ☐3 |
| 9. | ... to have difficulty hearing when someone speaks in a whisper? | ☐1 | ☐2 | ☐3 |
| 10. | ... to feel handicapped by a hearing problem? | ☐1 | ☐2 | ☐3 |
| 11. | ... to limit/hamper your personal or social life? | ☐1 | ☐2 | ☐3 |

12a. Do you wear a hearing aid?
   - ☐1 No → *Skip to page 10 (Psychosocial & Well Being)*
   - ☐2 Yes ↓

12b. <u>In the last 12 months</u> have you had your hearing aid tested by a specialist?
   - ☐1 No
   - ☐2 Yes

# Psychosocial Health & Well Being

*These questions are about how you feel and how things have been with you during the past month.*

| How much of the time, during the last month, have you … | | None of the time | A little of the time | Some of the time | A good bit of the time | Most of the time | All of the time |
|---|---|---|---|---|---|---|---|
| 1. | ... felt calm and peaceful? | ☐1 | ☐2 | ☐3 | ☐4 | ☐5 | ☐6 |
| 2. | ... been a very nervous person? | ☐1 | ☐2 | ☐3 | ☐4 | ☐5 | ☐6 |
| 3. | ... felt so down in the dumps that nothing could cheer you up? | ☐1 | ☐2 | ☐3 | ☐4 | ☐5 | ☐6 |
| 4. | ... felt downhearted and low? | ☐1 | ☐2 | ☐3 | ☐4 | ☐5 | ☐6 |
| 5. | ... been a happy person? | ☐1 | ☐2 | ☐3 | ☐4 | ☐5 | ☐6 |

*Questions 6 to 15 are about your memory. For each question, please choose the **ONE** answer that comes closest to describing the way you have been feeling.*

| ***Please select the best response for each of the following questions about your memory:*** | | Almost never | Some-times | Often | Almost always |
|---|---|---|---|---|---|
| 6. | Can you remember what clothes you wore yesterday? | ☐1 | ☐2 | ☐3 | ☐4 |
| 7. | In a large department store do you usually remember what entrance you used? | ☐1 | ☐2 | ☐3 | ☐4 |
| 8. | Can you recall where you parked your car or where your lift will pick you up? | ☐1 | ☐2 | ☐3 | ☐4 |
| 9. | In the dark can you distinguish between your car key and your house key? | ☐1 | ☐2 | ☐3 | ☐4 |
| 10. | Do you remember where you put your glasses or keys? | ☐1 | ☐2 | ☐3 | ☐4 |
| 11. | If someone calls you, can you give him or her directions to your house? | ☐1 | ☐2 | ☐3 | ☐4 |
| 12. | If you watch a TV series that runs for several nights, do you remember what happened in the last episode? | ☐1 | ☐2 | ☐3 | ☐4 |
| 13. | Can you describe what you did last Sunday afternoon? | ☐1 | ☐2 | ☐3 | ☐4 |
| 14. | Can you hum a tune after you have heard it several times? | ☐1 | ☐2 | ☐3 | ☐4 |
| 15. | Could you remember how to put back together a small appliance after you have taken it apart? | ☐1 | ☐2 | ☐3 | ☐4 |

# Social Network

*People sometimes look to others for friendship, assistance, or other types of support.*

*The following three questions are about your family members, including your spouse, in-laws, and any other relatives.*

1. How many relatives or family members do you see or hear from at least once a month? (Note: Include spouse, in-laws, and any other relatives.)
   - ☐1 Nine or more
   - ☐2 Five to eight
   - ☐3 Three or four
   - ☐4 Two
   - ☐5 One
   - ☐6 None

2. How many relatives or family members do you feel close to that you can call on for help? (Note: Include spouse, in-laws, and any other relatives.)
   - ☐1 Nine or more
   - ☐2 Five to eight
   - ☐3 Three or four
   - ☐4 Two
   - ☐5 One
   - ☐6 None

3. With how many relatives or family members can you comfortably discuss private matters? (Note: Include spouse, in-laws, and any other relatives.)
   - ☐1 Nine or more
   - ☐2 Five to eight
   - ☐3 Three or four
   - ☐4 Two
   - ☐5 One
   - ☐6 None

*The following three questions are about contacts with friends or neighbours.*

4. How many friends/neighbours do you see or hear from at least once a month?
   - ☐1 Nine or more
   - ☐2 Five to eight
   - ☐3 Three or four
   - ☐4 Two
   - ☐5 One
   - ☐6 None

5. How many friends/neighbours do you feel close to that you can call on for help?
   - ☐1 Nine or more
   - ☐2 Five to eight
   - ☐3 Three or four
   - ☐4 Two
   - ☐5 One
   - ☐6 None

6. With how many friends/neighbours can you comfortably discuss private matters?
   - ☐1 Nine or more
   - ☐2 Five to eight
   - ☐3 Three or four
   - ☐4 Two
   - ☐5 One
   - ☐6 None

*The next questions are about **social support and help**.*

7. How often do you have someone who shows you love and affection if you need it?

| | |
|---|---|
| ☐1 Always | ☐4 Sometimes |
| ☐2 Very Often | ☐5 Seldom |
| ☐3 Often | ☐6 Never |

8. How often do you have someone to share your most private worries and fears with if you need it?

| | |
|---|---|
| ☐1 Always | ☐4 Sometimes |
| ☐2 Very Often | ☐5 Seldom |
| ☐3 Often | ☐6 Never |

9. How often do you have someone to love and make you feel wanted?

| | |
|---|---|
| ☐1 Always | ☐4 Sometimes |
| ☐2 Very Often | ☐5 Seldom |
| ☐3 Often | ☐6 Never |

10. Do you have anyone (partner, relative, acquaintance, neighbour) who, if needed, could look after you for a few days?

☐1 No
☐2 Yes

*The next questions are about **social activities**.*

11. How often each month do you take part in organised activities (for example hobby or leisure groups, clubs, religious or political associations)?

| | |
|---|---|
| ☐1 Nine times or more | ☐4 Twice |
| ☐2 Between 5 and 8 times | ☐5 Once |
| ☐3 3 or 4 times | ☐6 Never |

12. How often do you help others, for example, if they are unwell, doing housework, providing meals, running errands, taking people to hospital, providing child care?

☐1 Never
☐2 Less than once a month
☐3 Once or twice a month
☐4 Once or more a week
☐5 Daily

# Functioning

*Loss of bladder control becomes more common with age; however, in many cases, bladder control can be improved.*

6a. In the last year, have you had leakage of urine and got wet?

☐1 No → *Skip to question 7 on this page*

☐2 Yes ↓

6b. ***If Yes***: Have you had leakage of urine on at least 6 days over the past 12 months?

☐1 No

☐2 Yes

*The following questions are about everyday activities.* ***Please say if you have difficulties with any of them, need the help of another person to do them, or have given up doing them entirely.***

| **Do you manage the following activities on your own or with help from others?** | | **Yes, without difficulty** | **Yes, with diffi-culty** | **Yes, with help from someone** |
|---|---|---|---|---|
| 7. | Get beyond walking distance of your home; for example, by driving or using public transport? | ☐1 | ☐2 | ☐3 |
| 8. | Shop for groceries | ☐1 | ☐2 | ☐3 |
| 9. | Prepare meals | ☐1 | ☐2 | ☐3 |
| 10. | Do housework | ☐1 | ☐2 | ☐3 |
| 11. | Manage money | ☐1 | ☐2 | ☐3 |

# Mobility

*The next questions are about **walking approximately 1 kilometre/half a mile.***

1. Can you walk 1 kilometre/ half a mile?
   - ☐1 No → *Skip to question 5 on this page*
   - ☐2 Yes

2. Is it difficult to walk 1 kilometre/ half a mile because of health problems or physical **difficulties**?
   - ☐1 No, I have no such difficulty
   - ☐2 Yes, I have difficulties

3. <u>In the last 12 months</u> have you altered **the way** in which you walk 1 kilometre/ half a mile? *(For example, by walking more slowly or more cautiously, by altering your posture or stride, by using a stick or similar aid, or by stopping more often than you used to)*
   - ☐1 No
   - ☐2 Yes

4. <u>In the last 12 months</u> have you walked 1 kilometre/ half a mile **less often**? *(For example, you no longer walk every day, only three or four times a week, perhaps because you tire more easily)*
   - ☐1 No
   - ☐2 Yes

*The next questions are about **climbing 10 steps**.*

5. Can you climb up 10 steps?
   - ☐1 No → *Skip to page 15 (Tobacco Use)*
   - ☐2 Yes

6. Do you have difficulties climbing 10 steps because of health problems or physical **difficulties**?
   - ☐1 No
   - ☐2 Yes

7. <u>In the last 12 months</u> have you altered **the way** in which you climb 10 steps? *(For example, you climb more slowly or more cautiously, or have altered your technique, or stop more often, or use the banisters more)*
   - ☐1 No
   - ☐2 Yes

8. <u>In the last 12 months</u> have you climbed 10 steps **less often**? *(For example, you seldom use steps, whenever possible you use the lift, perhaps because you have painful joints)*
   - ☐1 No
   - ☐2 Yes

# Tobacco Use

1. Are you currently a cigarette smoker?
   - ☐1 No, I have never smoked cigarettes
   - ☐2 No, I stopped more than 6 months ago
   - ☐3 No, I stopped in the last 6 months
   - ☐4 Yes, I am currently a cigarette smoker

2. Do you presently use tobacco in other forms (cigar or pipe)?
   - ☐1 No
   - ☐2 Yes

*The next questions apply to current and past smokers of tobacco.* ***If you have NEVER smoked please skip to page 16 Alcohol Use.***

***This section is for CURRENT smokers only.***

3. How many times in the last 12 months have you stopped smoking for at least 24 hours?
   - ☐1 never
   - ☐2 once
   - ☐3 more than once

4. Have you seriously thought about giving up smoking?
   - ☐1 Yes, I want to give up in the next 30 days
   - ☐2 Yes, I want to give up in the next 6 months
   - ☐3 No, I do not want to give up smoking

***For FORMER smokers only.***

5. When did you give up smoking regularly?
   - ☐1 Less than one year ago
   - ☐2 More than one year ago
   - ☐3 More than 5 years ago
   - ☐4 More than 10 years ago
   - ☐5 More than 15 years ago

***For CURRENT and FORMER smokers*.**

6. In the last 10 years have you had an ultrasound scan of the abdominal aorta?
   - ☐1 No
   - ☐2 Yes

# Alcohol Use

*Units of alcohol are counted as follows:*

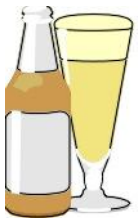

Half a pint (250ml) of medium strength beer = 1 unit

or

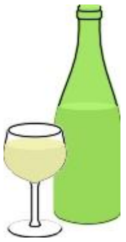

Medium size (175ml) wine glass = 2 units

or

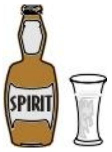


A single pub measure of spirits = 1 unit

or

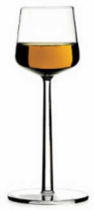

A small glass of sherry = 1 unit

*The next questions ask about how often you drink alcohol.*

1. In the last year how often have you drunk alcohol?
   - ☐ 1 Never → *Skip to page 18 (Physical Activity)*
   - ☐ 2 Monthly or less
   - ☐ 3 Two to four times a month
   - ☐ 4 Two to three times a week
   - ☐ 5 Four or more times a week

2. Thinking about a day in a typical month during the last year, when you did drink alcohol, how many units did you have (on average)?
   - ☐ 1 1 or 2
   - ☐ 2 3 or 4
   - ☐ 3 5 or 6
   - ☐ 4 7 to 9
   - ☐ 5 10 or more

3. In a typical month in the last year, on how many occasions did you have 6 or more units of alcohol?
   - ☐ 1 None
   - ☐ 2 Less than once a month
   - ☐ 3 Once a month
   - ☐ 4 Once a week
   - ☐ 5 Daily or almost daily

*The next questions are about your experience of drinking alcohol.*

4. Have you ever had the feeling that you should reduce the amount of alcohol that you drink?
   - ☐1 No
   - ☐2 Yes

5. Have you felt annoyed when other people criticise your alcohol consumption?
   - ☐1 No
   - ☐2 Yes

6. Have you felt guilty because of your alcohol consumption?
   - ☐1 No
   - ☐2 Yes

7. Have you ever had a drink of alcohol in the morning to calm your nerves or get rid of a hangover?
   - ☐1 No
   - ☐2 Yes

# Physical Activity

*The following questions are about your physically activity <u>in the last 7 days</u>. If the last seven days were not typical for you, think of a more typical week. Include activities you do at work, as part of your housework and gardening, to get from place to place, and in your spare time for leisure, exercise or sport. All the examples shown count as physical activity.*

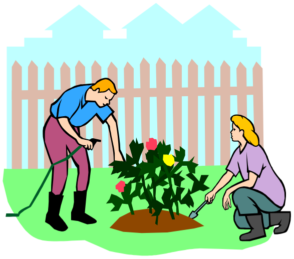
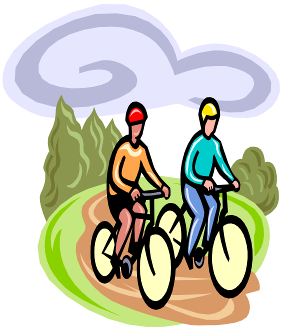
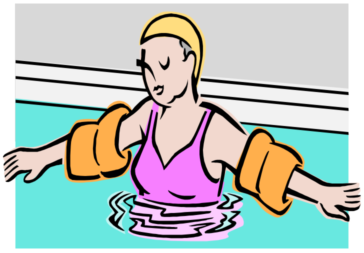
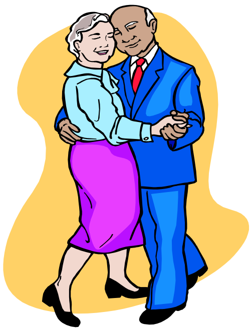

*Think about all the **vigorous physical activities** that you did <u>in the last 7 days</u>. **Vigorous activities** take hard physical effort and make you breathe much harder than normal. This includes heavy work of any sort, including in and around the home or outdoors such as heavy gardening (eg digging), leisure activities and sports like fast cycling (including on an exercise bike), jogging, aerobics or fitness training. Think only about those physical activities that you did for **at least 10 minutes** at a time.*

1. <u>During the last 7 days</u>, on how many days did you do **vigorous physical activities**?

   ☐1 Not at all → *Skip to page 19 question 3*
   ☐2 1 or 2 days a week
   ☐3 3 or 4 days a week
   ☐4 5 to 6 days a week
   ☐5 Everyday

2. How much time did you typically spend doing **vigorous physical activities** on <u>one of those days</u>?

   ☐1 About 10 minutes a day
   ☐2 About 20 minutes a day
   ☐3 About 30 minutes a day
   ☐4 About an hour a day
   ☐5 About 2 – 3 hours a day
   ☐6 4 or more hours a day

*Now, think about all the **moderate physical activities** that you did <u>in the last 7 days</u>. **Moderate activities** take some physical effort and make you breathe a bit harder than normal. Moderately vigorous activities can be done at work, in and around the house, in leisure time or sports like cycling at normal speed, brisk walking, swimming or dancing. Think only about those physical activities that you did for **at least 10 minutes** at a time.*

3. During the <u>last 7 days</u>, on how many days did you do **moderate physical activities**? Do not include walking at a normal pace?

   - ☐1 Not at all → *Skip to question 5 on this page*
   - ☐2 1 or 2 days a week
   - ☐3 3 or 4 days a week
   - ☐4 5 to 6 days a week
   - ☐5 Everyday

4. How much time did you typically spend doing **moderate physical activities** on <u>one of those days</u>?

   - ☐1 About 10 minutes a day
   - ☐2 About 20 minutes a day
   - ☐3 About 30 minutes a day
   - ☐4 About an hour a day
   - ☐5 About 2 – 3 hours a day
   - ☐6 4 or more hours a day

*Think about the time you spent **walking** <u>in the last 7 days</u>. This includes at work and at home, walking to travel from place to place, and any other walking that you might do solely for sport, exercise or leisure.*

5. During <u>the last 7 days</u>, on how many days did you **walk** for at least 10 minutes at a time?

   - ☐1 Not at all → *Skip to page 20 question 7*
   - ☐2 1 or 2 days a week
   - ☐3 3 or 4 days a week
   - ☐4 5 to 6 days a week
   - ☐5 Everyday

6. How much time did you usually spend **walking** on <u>one of those days</u>?

   - ☐1 About 10 minutes a day
   - ☐2 About 20 minutes a day
   - ☐3 About 30 minutes a day
   - ☐4 About an hour a day
   - ☐5 About 2 – 3 hours a day
   - ☐6 4 or more hours a day

*The last question is about the time you spent* ***sitting*** *during the last 7 days. Include time spent at work, at home, during a course or during leisure time. This may include time spent sitting at a desk, with friends, reading, or sitting (or lying down) to watch television.*

7. During the last 7 days, how much time did you spend **sitting** on a typical day?

☐1 Less than one hour a day
☐2 About one to two hours a day
☐3 About three to four hours a day
☐4 More than four hours a day

*In the next question we ask you if you would like to increase the level of your physical activity, and if not, what factors stop you.*

8. Do you intend to increase the amount of physical activity you do?

☐1 No, I do not intend to increase my level of physical activity
☐2 Yes, I intend to increase my level of physical activity in the next six months
☐3 Yes, I will increase my level of physical activity in the next month

9. Which of the following reasons prevent you from becoming more physically active?

a. ☐1 I am already regularly active
b. ☐1 I have no-one to do it with
c. ☐1 I do not know any activity opportunities suitable for my age group
d. ☐1 I do not have time
e. ☐1 Costs
f. ☐1 The weather
g. ☐1 I have an illness that limits my ability
h. ☐1 I have a physical disability
i. ☐1 I experience increased pain when I am physically active

# Nutrition

*The next questions are about your consumption of **fruit and vegetables** <u>in the last week</u>. If the last week was not a typical one, think back to a typical week. The examples below show how much fruit and vegetables make up a portion.*

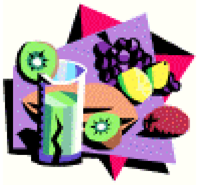

**Fruit or vegetable juice**
200ml of
unsweetened juice

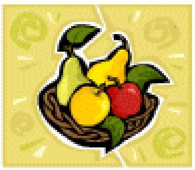

**Fruit and berries**

about 120g,
about one handful

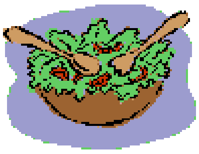

**Salad**

about 120g,
a small plate/bowl

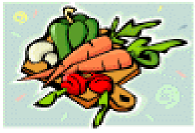

**Vegetables**

about 120g,
or one bowl of soup,
or half a cup of
tomato sauce

1. How often <u>in the last week</u> have you drunk a portion of **100% pure fruit juice** (***excluding** lemonade, vitamin drinks or other drinks that only contain some fruit)? Count all the fruit juices that you have had at or between main meals.*
   - ☐ 1 None in the last week
   - ☐ 2 Once or twice in the last week
   - ☐ 3 Three to six times per week
   - ☐ 4 Once a day
   - ☐ 5 Twice a day
   - ☐ 6 Three or more times a day

2. How often have you eaten a portion of **fruit or berries** <u>in the last week</u>? *(**Not counting** fruit juice) Include fresh, tinned and frozen fruits. Count all fruit and berries, eaten at or between main meals*
   - ☐ 1 None in the last week
   - ☐ 2 Once or twice in the last week
   - ☐ 3 Three to six times per week
   - ☐ 4 Once a day
   - ☐ 5 Twice a day
   - ☐ 6 Three or more times a day

3. How often <u>in the last week</u> have you eaten a portion of **salad** *(**without** sausage, cheese, potato or tuna in it)?*
   - ☐ 1 None in the last week
   - ☐ 2 Once or twice in the last week
   - ☐ 3 Three to six times per week
   - ☐ 4 Once a day
   - ☐ 5 Twice a day
   - ☐ 6 Three or more times a day

4. How often in the last week have you eaten a portion of **vegetables** *(**not including** salad or potatoes)? Count raw, cooked, tinned and frozen vegetables.*
   - ☐1 None in the last week
   - ☐2 Once or twice in the last week
   - ☐3 Three to six times per week
   - ☐4 Once a day
   - ☐5 Twice a day
   - ☐6 Three or more times a day

5. How often in the last week have you eaten a portion of **vegetable soup**? *(**Include** tomato soup, meat soup with vegetables, minestrone or other vegetable soups)*
   - ☐1 None in the last week
   - ☐2 Once or twice in the last week
   - ☐3 Three to six times per week
   - ☐4 Once a day
   - ☐5 Twice a day
   - ☐6 Three or more times a day

6. How often in the last week have you eaten a portion of **tomato sauce (not ketchup)**? *(**Include** tomato sauce eaten with pasta, on a pizza or in any other dish)*
   - ☐1 None in the last week
   - ☐2 Once or twice in the last week
   - ☐3 Three to six times per week
   - ☐4 Once a day
   - ☐5 Twice a day
   - ☐6 Three or more times a day

7. Do you intend to increase the amount of fruit and vegetables in your diet?
   - ☐1 No, I do not intend to increase the amount of fruit and vegetables that I eat
   - ☐2 Yes, I intend to increase the amount of fruit and vegetables I eat in the next 6 months
   - ☐3 Yes, I will increase the amount of fruit and vegetables I eat in the next month

*The following questions are general ones about nutrition.*

8. Are you aware of specific nutritional guidelines?
   - ☐1 No
   - ☐2 Yes

9. In the last 12 months have you discussed nutrition with your doctor or with a nutritionist?
   - ☐1 No
   - ☐2 Yes

10. In the last three months have you eaten less because of lack of appetite, intestinal problems, or difficulties with chewing or swallowing?
    - ☐1 No
    - ☐2 Yes

*The following questions are about your consumption of* ***high-fat foods.***
***Tick ONE answer for each food category.***

| How often do you eat . . .? | | Never or less than once per MONTHS | 1-3 times per MONTHS | 1-2 times per WEEK | 3-4 times per WEEK | 5 times or more per WEEK |
|---|---|---|---|---|---|---|
| 11. | Beef or pork, such as steak, roasts, chops | ☐1 | ☐2 | ☐3 | ☐4 | ☐5 |
| 12. | Fried chicken with skin, fried fish | ☐1 | ☐2 | ☐3 | ☐4 | ☐5 |
| 13. | Hamburgers, cheeseburgers, minced beef | ☐1 | ☐2 | ☐3 | ☐4 | ☐5 |
| 14. | Sausages | ☐1 | ☐2 | ☐3 | ☐4 | ☐5 |
| 15. | Cheese spread or cheese (not low-fat) | ☐1 | ☐2 | ☐3 | ☐4 | ☐5 |
| 16. | Whole milk, semi-skimmed milk or cream | ☐1 | ☐2 | ☐3 | ☐4 | ☐5 |
| 17. | Margarine, butter or gravy | ☐1 | ☐2 | ☐3 | ☐4 | ☐5 |
| 18. | Salad cream (not diet) or mayonnaise | ☐1 | ☐2 | ☐3 | ☐4 | ☐5 |
| 19. | French fries, fried potatoes, or chips | ☐1 | ☐2 | ☐3 | ☐4 | ☐5 |
| 20. | Crisps, popcorn, cracker biscuits | ☐1 | ☐2 | ☐3 | ☐4 | ☐5 |
| 21. | Chocolates, sweets, biscuits | ☐1 | ☐2 | ☐3 | ☐4 | ☐5 |
| 22. | Ice cream (not low-fat), doughnuts, pastries, pies, cake, cookies, biscuits | ☐1 | ☐2 | ☐3 | ☐4 | ☐5 |

23. Do you intend to reduce the amount of high fat food you eat?

☐1 No, I do not intend to reduce the amount of high fat food I eat
☐2 Yes, I intend to reduce the amount of high fat food I eat in the next 6 months
☐3 Yes, I will reduce the amount of high fat food I eat in the next month

# Falls Prevention

1. Do you limit your activities because you are afraid you will fall?
   - ☐1 No
   - ☐2 Yes

2a. During the past 12 months have you fallen to the ground or floor?
   - ☐1 No → *Skip to next section*
   - ☐2 Yes ↓

2b. How many times did you fall
   - ☐1 Once
   - ☐2 More than once

# Preventive Care

*The next questions are about health care services that you may have had.* ***Please answer each question with NO or YES.***

| **Have you had a ...** | | **No** | **Yes** |
|---|---|---|---|
| 1. | Blood pressure measurement within the past year? | ☐1 | ☐2 |
| 2. | Fasting blood glucose measurement within the past 3 years? | ☐1 | ☐2 |
| 3. | Cholesterol measurement within the past 5 years? | ☐1 | ☐2 |
| 4. | Eyesight checkup within the past 2 years? | ☐1 | ☐2 |
| 5. | Hearing checkup within the past year? | ☐1 | ☐2 |
| 6. | Immunisation against influenza (“flu”) within the past year? | ☐1 | ☐2 |

# About You

1. Where were you born?
   - ☐1 Romania
   - ☐2 In another country

2. What is your first language?
   - ☐1 Romanian
   - ☐2 Another language

3. What are your current living arrangements (Please choose one of the following)?
   - ☐1 I live alone
   - ☐2 I live with my spouse/partner
   - ☐3 I live with another family member / other family members
   - ☐4 I live with a friend/someone else
   - ☐5 other

4. Do you live in a…?
   - ☐1 Flat (ground floor or lift accessible)
   - ☐2 Flat (other)
   - ☐3 House

5. What level of education did you receive?
   - ☐1 University
   - ☐2 Vocational Training
   - ☐3 High-school
   - ☐4 Secondary school (8 classes)
   - ☐5 Primary school (4 classes)
   - ☐6 No formal education (illiteracy)

6. What type of work did you do most of your life?
   ***Please tick only ONE response.***
   - ☐1 Employee with management functions
   - ☐2 Employee without management functions
   - ☐3 Self-employed
   - ☐4 Housewife/husband

7. What is your marital status?
   - ☐1 Married
   - ☐2 Divorced/Separated
   - ☐3 Widow/er

# Acceptability Questionnaire

*This brief survey asks questions about your recent experience completing the Older Persons Health Profile Questionnaire. Your honest responses will help us improve it for future use with others.*

1. In general, how difficult was it for you to understand the questions and instructions in the Questionnaire?
   - ☐ 1 Very difficult
   - ☐ 2 Somewhat difficult
   - ☐ 3 Somewhat easy
   - ☐ 4 Very easy

If you answered **Very difficult** or **Somewhat difficult**, what sections, questions, or instructions did you find difficult to understand?

---

2. In general, how difficult was it for you to complete the Questionnaire?
   - ☐ 1 Very difficult
   - ☐ 2 Somewhat difficult
   - ☐ 3 Somewhat easy
   - ☐ 4 Very easy

If you answered **Very difficult** or **Somewhat difficult**, what sections or questions did you find difficult to complete?

---

3. Please mark the response that best corresponds to the assistance you received in completing this questionnaire.
   - ☐ 1 Someone else completed the Questionnaire for me.
   - ☐ 2 I completed it with assistance from someone else.
   - ☐ 3 I competed it without assistance, but would have liked assistance.
   - ☐ 4 I completed it without assistance, and not feel I needed assistance .

4. How would you describe the length of the Questionnaire?
   - ☐ 1 Too long
   - ☐ 2 About right
   - ☐ 3 Too short

5. About how much time did it take you to complete the Questionnaire?

☐☐ minutes

6. Did you dislike any specific sections or questions on the Questionnaire?

☐ 1 No
☐ 2 Yes

**If you answered Yes,** what sections or questions did you dislike?

---

7. Are there any topic areas that were not covered in the Questionnaire that you think should have been covered in the Questionnaire?

☐ 1 No
☐ 2 Yes

**If you answered Yes,** what topic areas should have been covered?

---

*Please take a moment to look back and make sure you have not missed any pages or questions.*

## Thank you for completing this questionnaire!

Version 2.5: This questionnaire was developed by the Geriatric Research Unit Bern (Spital Bern Ziegler, Morillonstrasse 75, CH- 3001 Bern/Switzerland) in collaboration with the Department of Health Care of the Elderly, King's College London, the Department of Primary Care and Population Sciences, University College London, and other organisations based on a license to use granted by the Regents of the University of California, Los Angeles. This questionnaire is protected by copyright and may not (as a whole or in part) be reproduced or used for other purposes without prior written permission of the copyright owners.
Version 2.5: © 2000, Regents of the University of California, Los Angeles.
Section "Physical activity": © 1991 New England Research Institutes, 9 Galen Street, Watertown, MA 02472 USA.

© 2013 Geriatric Research Fund, Spital Netz Bern AG, Inselspital, PO Box 20, CH-3010 Bern, Switzerland. This questionnaire is protected by copyright and may not (as a whole or in part) be reproduced or used for other purposes without prior written permission of the copyright owners.

# Profil de Sănătate pentru Persoane Vârstnice HRA-O

# Chestionar Inițial

*Va rugăm să nu completați acest tabel.*

ID-number ☐☐☐☐☐☐☐☐☐☐☐☐☐☐☐☐☐☐

Study Group ☐ 1 **A** Intervention ☐ 2 **B** Placebo

Location ☐ 1 Hospital ☐ 2 Out patient

First name ☐☐☐☐☐☐☐☐☐☐☐☐☐☐☐☐☐☐

Surname ☐☐☐☐☐☐☐☐☐☐☐☐☐☐☐☐☐☐

| Date of examination | Day | Month | Year |
|---|---|---|---|
| | ☐☐ | ☐☐ | ☐☐☐☐ |

*Vă rugăm să completați acest chestionar.*

*Vă rugăm să:*

- *Citiți fiecare întrebare cu atenție și urmăriți instrucțiunile.*
- *Puneți un X în pătratul relevant pentru fiecare întrebare astfel:* ☒
- *Nu scrieți comentarii în spațiile libere sau pe margini, nu vor fi luate în considerare.*
- *Răspundeți întrebărilor de la fiecare capitol, chiar dacă subiectul respectiv nu se aplică în cazul dvs.*
- *La sfârșit verificați dacă ați completat fiecare pagină și dacă ați răspuns la toate întrebările de pe fiecare pagină.*

© 2014 Acest chestionar este protejat prin legea dreptului de autor 

# Medicamente/Rețete

*Următoarele întrebări sunt despre medicamentele pe care dvs. le luați cu rețetă sau direct de la farmacie (inclusiv cele din plante).* ***Vă rugăm includeți și cele pe care le luați doar la nevoie.***

1. Câte medicamente sub formă de pastile, tablete, capsule sau sub formă lichidă ați luat în ultimele 7 zile? Ultimul răspuns este „10 sau mai multe”?

| **0** | **1** | **2** | **3** | **4** | **5** | **6** | **7** | **8** | **9** | **Mai multe** |
|---|---|---|---|---|---|---|---|---|---|---|
| ☐ 0 | ☐ 1 | ☐ 2 | ☐ 3 | ☐ 4 | ☐ 5 | ☐ 6 | ☐ 7 | ☐ 8 | ☐ 9 | ☐ 10 |

2. În ultimele 7 zile ați luat și alte medicamente, însă care **nu** se înghit?

| | | | |
|---|---|---|---|
| a. | Picături pentru ochi | ☐ 1 Da | ☐ 2 Nu |
| b. | Plasturi ce conțin medicamente | ☐ 1 Da | ☐ 2 Nu |
| c. | Injecții | ☐ 1 Da | ☐ 2 Nu |
| d. | medicamente inhalate *(ex.pentru astm)* | ☐ 1 Da | ☐ 2 Nu |
| e. | Supozitoare | ☐ 1 Da | ☐ 2 Nu |

*Următoarele întrebări sunt despre* ***medicamentele, în orice formă, pe care le-ați luat*** *în ultimele 7 zile.*

| Ați luat vreunul din următoarele medicamente în ultimele 7 zile**?** | | **Nu** | **Da** |
|---|---|---|---|
| 3. | Medicamente pentru tensiune arterială crescută | ☐ 1 | ☐ 2 |
| 4. | Medicamente pentru colesterol crescut | ☐ 1 | ☐ 2 |
| 5. | Medicamente pentru diabet | ☐ 1 | ☐ 2 |
| 6. | Medicamente pentru boli de inimă | ☐ 1 | ☐ 2 |

© 2014 Acest chestionar este protejat prin legea dreptului de autor

| | | | |
|---|---|---|---|
| 7. | Anticoagulante *(pentru subțierea sângelui)* cum sunt Sintrom sau Pradaxa sau antiagregante plachetare cum sunt Aspirina sau Clopidogrel | ☐1 | ☐2 |
| 8. | Medicamente care vă ajută să dormiți | ☐1 | ☐2 |
| 9. | Medicamente pentru durere | ☐1 | ☐2 |
| 10. | Medicamente pentru stări de nervozitate, anxietate | ☐1 | ☐2 |
| 11. | Medicamente pentru stări depresive, de tristețe | ☐1 | ☐2 |
| 12. | Medicamente pentru boala Alzheimer | ☐1 | ☐2 |
| *(Doar pentru doamne):* | | | |
| 13. | Medicamente pe bază de hormoni *(estrogeni)* | ☐1 | ☐2 |

14. Sunteți alergic/ă la vreun medicament?

- ☐ 1 Nu
- ☐ 2 Da

*Următoarele întrebări sunt despre eventualele probleme sau dificultăți pe care le-ați putea avea atunci când luați medicamente*

***Dacă în prezent NU luați nici un medicament, mergeți la pagina 5.***

15. Aveți o listă și un plan *(schemă)* cu medicamentele pe care trebuie să le luați?

- ☐ 1 Nu
- ☐ 2 Da

16. Folosiți un organizator *(cutie)* de medicamente?

- ☐ 1 Nu
- ☐ 2 Da

17. Întâmpinați dificultăți în a înțelege când și cum trebuie să vă luați medicamentele?

- ☐ 1 Nu
- ☐ 2 Da

18. Există vreun medicament pe care nu sunteți sigur/ă de ce îl luați?

- ☐ 1 Nu
- ☐ 2 Da

19. Luați medicamente prescrise de doi sau mai mulți medici?

- ☐ 1 Nu
- ☐ 2 Da

20. Credeți că suferiți vreo reacție adversă sau simptome cauzate de medicamentele pe care le luați?

- ☐ 1 Nu
- ☐ 2 Da

© 2014 Acest chestionar este protejat prin legea dreptului de autor 

# Durere

*Următoarele întrebări sunt despre durere, cât de severă este și dacă vă afectează viața de zi cu zi. Primele 2 întrebări sunt despre intensitatea durerii pe care o simțiți, pe o scală de la 0 la 10; „0" înseamnă „nici o durere" , iar „10" înseamnă „cea mai mare durere pe care v-o puteți imagina"*

1. Cât de severă este durerea **dvs. Astăzi**?

| 0 | 1 | 2 | 3 | 4 | 5 | 6 | 7 | 8 | 9 | 10 |
|---|---|---|---|---|---|---|---|---|---|---|
| ☐ 0 | ☐ 1 | ☐ 2 | ☐ 3 | ☐ 4 | ☐ 5 | ☐ 6 | ☐ 7 | ☐ 8 | ☐ 9 | ☐ 10 |

nici o durere — cea mai mare durere

2. Cât de severă a fost durerea dvs ***(în medie)* în ultimele 7 zile ?**

| 0 | 1 | 2 | 3 | 4 | 5 | 6 | 7 | 8 | 9 | 10 |
|---|---|---|---|---|---|---|---|---|---|---|
| ☐ 0 | ☐ 1 | ☐ 2 | ☐ 3 | ☐ 4 | ☐ 5 | ☐ 6 | ☐ 7 | ☐ 8 | ☐ 9 | ☐ 10 |

nici o durere — cea mai mare durere

| Din cauza **durerii**, ... | | Nu | Da |
|---|---|---|---|
| 3. | ... ați redus timpul petrecut cu munca sau alte activități? | ☐ 1 | ☐ 2 |
| 4. | ... ați realizat mai puține lucruri decât ați fi dorit? | ☐ 1 | ☐ 2 |
| 5. | ... a trebuit să renunțați la muncă sau alte activități? | ☐ 1 | ☐ 2 |
| 6. | ... munca sau alte activități pe care le desfășurați necesită un efort mai mare? | ☐ 1 | ☐ 2 |
| 7. | ... aveți probleme cu somnul? | ☐ 1 | ☐ 2 |
| 8. | ... vă întâlniți mai rar cu prietenii sau rudele? | ☐ 1 | ☐ 2 |

© 2014 Acest chestionar este protejat prin legea dreptului de autor 

*Următoarele întrebări sunt despre durerea pe care o simțiți în timp ce faceți diferite activități. Alegeți răspunsul cel mai apropiat de situația dvs.*

| **În prezent simțiți durere în timpul următoarelor activități?** | | **Nu** | **Da** |
|---|---|---|---|
| 9. | Mers 200 de metri sau mai puțin | ☐ 1 | ☐ 2 |
| 10. | Mers mai mult de 200 de metri | ☐ 1 | ☐ 2 |
| 11. | Urcat mai mult de un etaj pe scări | ☐ 1 | ☐ 2 |
| 12. | Activități fizice moderate cum ar fi : mutat o masă mai grea, dat cu aspiratorul, hranit animale ? | ☐ 1 | ☐ 2 |

© 2014 Acest chestionar este protejat prin legea dreptului de autor

# Văz (Vedere)

*Următoarele întrebări sunt despre problemele pe care le aveți cu vederea sau părerea dvs. despre situția în care se află vederea dvs. Pentru fiecare întrebare vă rugăm alegeți răspunsul care descrie cel mai bine situația dvs.* ***Dacă purtați ochelari de vedere sau lentile de contact, răspundeți considerând că le purtați. Alegeți răspunsul care descrie cel mai bine situația dvs.***

1. În prezent ați spune că vederea dvs. *(purtând ochelari sau lentile de contact dacă aveți)* este:

   - ☐ 1 Excelentă *Mergeți la pagina 10 (Auz)*
   - ☐ 2 Bună
   - ☐ 3 Potrivită
   - ☐ 4 Proastă
   - ☐ 5 Foarte proastă
   - ☐ 6 Sunt complet orb/oarbă

*Următoarele întrebări sunt despre cât de dificil (dacă este cazul) este pentru dvs. să desfășurați anumite activități (purtând ochelari de vedere sau lentile de contact dacă le folosiți pentru acea activitate)*

2. Cât de dificil este pentru dvs. <u>să citiți un articol dintr-un ziar</u>? Ați spune că:

   - ☐ 1 Nu e deloc dificil
   - ☐ 2 E puțin dificil
   - ☐ 3 E destul de dificil
   - ☐ 4 E foarte dificil
   - ☐ 5 Nu mai citesc ziarul din cauza vederii proaste
   - ☐ 6 Nu mai citesc ziarul din alte motive sau nu mă interesează ziarele

© 2014 Acest chestionar este protejat prin legea dreptului de autor

3. Cât de dificil este pentru dvs. să vă desfășurați munca sau alte activități care necesită o bună vedere de aproape *(gătit, cusut, mici reparații în casă, alte activități pentru care folosiți unelte de mână)*? Ați spune că:

- ☐ 1 Nu e deloc dificil
- ☐ 2 E puțin dificil
- ☐ 3 E destul de dificil
- ☐ 4 E foarte dificil
- ☐ 5 Am încetat această activitate din cauza vederii
- ☐ 6 Am încetat această activitate din alte motive decât vederea sau nu mă interesează

4. Din cauza vederii, cât de dificil este pentru dvs. să coborâți scările sau să mergeți pe un drum denivelat *(să treceți de un prag)* în lumină slabă sau noaptea? Ați spune că:

- ☐ 1 Nu e deloc dificil
- ☐ 2 E puțin dificil
- ☐ 3 E destul de dificil
- ☐ 4 E foarte dificil
- ☐ 5 Am încetat această activitate din cauza vederii
- ☐ 6 Am încetat această activitate din alte motive

5. Din cauza vederii, cât de dificil este pentru dvs. să observați obiectele aflate pe margini/în lateral atunci când mergeți ? Ați spune că:

- ☐ 1 Nu e deloc dificil
- ☐ 2 E puțin dificil
- ☐ 3 E destul de dificil
- ☐ 4 E foarte dificil
- ☐ 5 Am încetat această activitate din cauza vederii
- ☐ 6 Am încetat această activitate din alte motive

6. Din cauza vederii, cât de dificil este pentru dvs. să găsiți un obiect aflat pe un raft aglomerat aflat în apropierea dvs. ? Ați spune că:

- ☐ 1 Nu e deloc dificil
- ☐ 2 E puțin dificil
- ☐ 3 E destul de dificil
- ☐ 4 E foarte dificil
- ☐ 5 Am încetat această activitate din cauza vederii
- ☐ 6 Am încetat această activitate din alte motive

© 2014 Acest chestionar este protejat prin legea dreptului de autor

7. Durata muncii sau a altor activități pe care le desfășurați este limitată din cauza vederii?

- ☐ 1 Niciodată
- ☐ 2 Câteodată
- ☐ 3 Adesea
- ☐ 4 Cel mai adesea
- ☐ 5 Tot timpul

*Următoarele întrebări se referă la șofat (condus mașina)..*

8a. Conduceți mașina în prezent, cel puțin câteodată?

- ☐ 1 Da — *Mergeți la întrebarea 9 de pe această pagină*
- ☐ 2 Nu

8b. ***Dacă Nu***: Ați condus vreodată o mașina sau ați renunțat la această activitate?

- ☐ 1 Nu am condus niciodată — *Mergeți la pagina 10 (Auz)*
- ☐ 2 Am renunțat la șofat

8c. De ce ați renunțat la șofat? În special din cauza vederii, din alte motive sau atât din cauza vederii cât și din alte motive?

- ☐ 1 În special din cauza vederii
- ☐ 2 Din alte motive
- ☐ 3 Atât din cauza vederii cât și din alte motive

*Următoarea întrebare este pentru cei care conduc mașina în prezent.*

9. Cât de dificil este pentru dvs. să conduceți mașina în timpul zilei în locuri familiare? Ați spune că:

- ☐ 1 Nu este dificil deloc
- ☐ 2 Este puțin dificil
- ☐ 3 Este destul de dificil
- ☐ 4 Este extrem de dificil

© 2014 Acest chestionar este protejat prin legea dreptului de autor

# Auz

*Următoarele întrebări sunt despre problemele pe care le aveți cu auzul.* ***Dacă folosiți o proteză/, dispozitiv auditiv, vă rugăm răspundeți considerând că o purtați.***

1. Considerați că auzul dvs. este *(dacă este cazul, când purtați proteza/dispozitiv auditiv)*?
   - ☐ 1 Excelent
   - ☐ 2 Foarte bun
   - ☐ 3 Bun
   - ☐ 4 Prost
   - ☐ 5 Foarte prost
   - ☐ 6 Nu aud deloc

| **Din cauza auzului** ***(dacă este cazul, atunci când purtați proteza auditivă)*** **se întâmplă să...** | | **Nu** | **Uneori** | **Da** |
|---|---|---|---|---|
| 2. | ... vă simțiți jenat/ă când faceți cunoștință cu persoane noi? | ☐ 1 | ☐ 2 | ☐ 3 |
| 3. | ... vă simțiți frustrat/ă când vorbiți cu membrii familiei? | ☐ 1 | ☐ 2 | ☐ 3 |
| 4. | ... aveți dificultăți când vă întâlniți cu prietenii, vecinii? | ☐ 1 | ☐ 2 | ☐ 3 |
| 5. | ... participați mai rar decât ați dori la slujbe religioase sau piese de teatru? | ☐ 1 | ☐ 2 | ☐ 3 |
| 6. | ... aveți dispute cu membrii familiei? | ☐ 1 | ☐ 2 | ☐ 3 |
| 7. | ... aveți dificultăți când ascultați radioul sau televizorul? | ☐ 1 | ☐ 2 | ☐ 3 |
| 8. | ... aveți dificultăți într-un local sau restaurant cu familia sau prietenii? | ☐ 1 | ☐ 2 | ☐ 3 |
| 9. | ... aveți dificultăți să auziți atunci când cineva vorbește în șoaptă? | ☐ 1 | ☐ 2 | ☐ 3 |
| 10. | ... simțiți că aveți un handicap? | ☐ 1 | ☐ 2 | ☐ 3 |
| 11. | ... simțiți că viața personală sau socială vă este limitată? | ☐ 1 | ☐ 2 | ☐ 3 |

© 2014 Acest chestionar este protejat prin legea dreptului de autor

12a. Purtați o proteză /dispozitiv auditiv?

☐ 1 Nu *Mergeți la pagina 12 (Psihosocial și starea de bine)*

☐ 2 Da

12b. Ați fost la un medic specialist să vă testeze auzul în ultimele 12 luni?

☐ 1 Nu

☐ 2 Da

© 2014 Acest chestionar este protejat prin legea dreptului de autor 

# Starea de sănătate psihosocială și starea de bine

*Aceste întrebări sunt despre cum v-ați simțit și cum v-a mers **în ultima lună**.*

| Cât de des în ultima lună... | | Niciodată | Rareori | Câteodată | Destul de des | Aproape tot timpul | Tot timpul |
|---|---|---|---|---|---|---|---|
| 1. | ... v-ați simțit calm și împăcat? | ☐ 1 | ☐ 2 | ☐ 3 | ☐ 4 | ☐ 5 | ☐ 6 |
| 2. | ... ați fost nervos /nervoasă? | ☐ 1 | ☐ 2 | ☐ 3 | ☐ 4 | ☐ 5 | ☐ 6 |
| 3. | ... v-ați simțit trist și deprimat și nimic nu v-a putut înveseli? | ☐ 1 | ☐ 2 | ☐ 3 | ☐ 4 | ☐ 5 | ☐ 6 |
| 4. | ... v-ați simțit descurajat și fără energie? | ☐ 1 | ☐ 2 | ☐ 3 | ☐ 4 | ☐ 5 | ☐ 6 |
| 5. | ... v-ați simțit fericit/ă? | ☐ 1 | ☐ 2 | ☐ 3 | ☐ 4 | ☐ 5 | ☐ 6 |

*Întrebările 6-15 sunt despre memoria dvs. Pentru fiecare întrebare vă rugăm alegeți doar **UN SINGUR** răspuns, cel care descrie cel mai bine cum vă simțiți.*

| Vă rugăm selectați, pentru fiecare întrebare, răspunsul cel mai potrivit pentru dvs. | | Aproape niciodată | Câteodată | Destul de des | Aproape întotdeauna |
|---|---|---|---|---|---|
| 6. | Vă amintiți ce haine ați purtat ieri? | ☐ 1 | ☐ 2 | ☐ 3 | ☐ 4 |
| 7. | Atunci când vă aflați într-un magazin mare vă amintiți pe ce intrare ați venit? | ☐ 1 | ☐ 2 | ☐ 3 | ☐ 4 |
| 8. | Vă amintiți unde ați parcat mașina sau unde trebuie să vă întâlniți cu cineva? | ☐ 1 | ☐ 2 | ☐ 3 | ☐ 4 |
| 9. | Puteți distinge între cheile de la mașină și cheile de la casă pe întuneric? | ☐ 1 | ☐ 2 | ☐ 3 | ☐ 4 |
| 10. | Vă amintiți unde ați pus ochelarii sau cheile? | ☐ 1 | ☐ 2 | ☐ 3 | ☐ 4 |

© 2014 Acest chestionar este protejat prin legea dreptului de autor

| | | | | | |
|---|---|---|---|---|---|
| 11. | Dacă cineva vă întreabă, îi puteți explica cum să ajungă la locuința dvs.? | ☐ 1 | ☐ 2 | ☐ 3 | ☐ 4 |
| 12. | Dacă urmăriți un serial, vă amintiți despre ce a fost vorba și ce s-a întâmplat în ultimul episod văzut? | ☐ 1 | ☐ 2 | ☐ 3 | ☐ 4 |
| 13. | Puteți spune ce ați făcut în după-amiaza de duminica trecuta? | ☐ 1 | ☐ 2 | ☐ 3 | ☐ 4 |
| 14. | Puteți fredona o melodie pe care ați ascultat-o de câteva ori? | ☐ 1 | ☐ 2 | ☐ 3 | ☐ 4 |
| 15. | Vă amintiți cum să asamblați la loc o mică unealtă sau alt obiect după ce l-ați desfăcut? | ☐ 1 | ☐ 2 | ☐ 3 | ☐ 4 |

© 2014 Acest chestionar este protejat prin legea dreptului de autor

# Rețea socială

*Uneori oamenii privesc către ceilalți căutând prieteni, suport sau alt fel de ajutor.*

*Următoarele 3 întrebări sunt despre familia dvs. , inclusiv soț/soție, socri și alte rude.*

1. Cu câți dintre membrii familiei sau alte rude vorbiți sau vă întâlniți cel puțin o dată pe lună ? *(Notă: inclusiv soț/soție, socri sau oricare alte rude.)*

☐1 9 sau mai mulți ☐4 2
☐2 5 sau 8 ☐5 1
☐3 3 sau 4 ☐6 nici unul

2. De câți dintre membrii familiei sau alte rude vă simțiți suficient de apropiat astfel încât le puteți cere ajutorul ? *(Notă: inclusiv soț/soție, socri sau oricare alte rude.)*

☐1 9 sau mai mulți ☐4 2
☐2 5 sau 8 ☐5 1
☐3 3 sau 4 ☐6 nici unul

3. Cu câti dintre membrii familiei sau alte rude este comfortabil pentru dvs. să discutați subiecte personale? *(Notă: inclusiv soț/soție, socri sau oricare alte rude.)*

☐1 9 sau mai mulți ☐4 2
☐2 5 sau 8 ☐5 1
☐3 3 sau 4 ☐6 nici unul

*Următoarele 3 întrebări se referă la contactele pe care dvs. le aveți cu prietenii sau vecinii.*

4. Cu câți prieteni/vecini vorbiți sau vă întâlniți <u>cel puțin o dată pe lună</u>?

☐1 9 sau mai mulți e ☐4 2
☐2 5 sau 8 ☐5 1
☐3 3 sau 4 ☐6 nici unul

© 2014 Acest chestionar este protejat prin legea dreptului de autor 

5. De câți prieteni / vecini vă simțiți suficient de apropiați pentru a le cere ajutorul?

| | |
|---|---|
| ☐1 9 sau mai mulți | ☐4 2 |
| ☐2 5 sau 8 | ☐5 1 |
| ☐3 3 sau 4 | ☐6 nici unul |

6. Cu câți dintre prietenii / vecinii dvs. este comfortabil pentru dvs. sa discutați subiecte personale?

| | |
|---|---|
| ☐1 9 sau mai mulți | ☐4 2 |
| ☐2 5 sau 8 | ☐5 1 |
| ☐3 3 sau 4 | ☐6 nici unul |

*Următoarele întrebări se referă la* ***sprijinul și ajutorul social****.*

7. Cât de des aveți pe cineva care să vă arate iubire și afecțiune atunci când aveți nevoie?

| | |
|---|---|
| ☐1 Întotdeauna | ☐4 Câteodată |
| ☐2 Foarte des | ☐5 Foarte rar |
| ☐3 Des | ☐6 Niciodată |

8. Cât de des aveți pe cineva cu care să împărtășiți temerile și îngrijorările dvs. atunci când aveți nevoie?

| | |
|---|---|
| ☐1 Întotdeauna | ☐4 Câteodată |
| ☐2 Foarte des | ☐5 Foarte rar |
| ☐3 Des | ☐6 Niciodată |

9. Cât de des aveți pe cineva care să vă iubească și să vă facă să vă simțiți dorit/ă?

| | |
|---|---|
| ☐1 Întotdeauna | ☐4 Câteodată |
| ☐2 Foarte des | ☐5 Foarte rar |
| ☐3 Des | ☐6 Niciodată |

10. Aveți pe cineva *(soț/soție, partener/ă, cunoștință, vecin)* care la nevoie ar putea să vă îngrijească pentru câteva zile?

| | |
|---|---|
| ☐1 Nu | ☐2 Da |

© 2014 Acest chestionar este protejat prin legea dreptului de autor

11. Cât de des în fiecare lună luați parte la activități organizate *(de exemplu : grupuri de lectură, cluburi, asociații politice sau religioase)*?

- ☐ 1 9 sau mai multe ori
- ☐ 2 5 sau 8 ori
- ☐ 3 3 sau 4 ori
- ☐ 4 2 ori
- ☐ 5 1 dată
- ☐ 6 niciodată

12. Cât de des îi ajutați pe alții, atunci când nu se simt bine, de exemplu cu treburile casei, să le duceți de mâncare, să faceți comisioane, să-i duceți la medic sau să-i ajutați cu copiii?

- ☐ 1 Niciodată
- ☐ 2 Mai puțin de o dată pe lună
- ☐ 3 O dată sau de două ori pe lună
- ☐ 4 O dată sau de două ori pe săptămână
- ☐ 5 În fiecare zi

© 2014 Acest chestionar este protejat prin legea dreptului de autor

# Funcționare

*Incontinența urinară (pierderea de urină) devine o problemă frecventă odată cu înaintarea în vârstă ; totusi, în unele cazuri, se poate interveni pentru ameliorarea acestei probleme.*

1a. Ați avut pierderi de urină și v-ați udat în ultimul an?

☐ 1 Nu — *Mergeți la întrebarea 2 pe această pagină*

☐ 2 Da

1b. Dacă ați răspuns **Da**:

Ați avut pierderi de urină în cel puțin 6 zile pe parcursul ultimelor 12 luni?

☐ 1 Nu

☐ 2 Da

*Următoarele întrebări se referă la activitățile zilnice.* ***Vă rugăm semnalați dacă aveți dificultăți cu oricare dintre acestea, dacă aveți nevoie de ajutorul altei persoane pentru a le face sau dacă ați renunțat complet la ele.***

| | **Puteți desfășura următoarele activități singur/ă sau cu ajutorul altora ?** | **Da, fără dificultate** | **Da, cu dificultate** | **Da, cu ajutorul altora** |
|---|---|---|---|---|
| 2. | vă îndepărtați de casă la o distanță mai mare, de exemplu cu mașina sau cu mijloacele de transport în comun? | ☐ 1 | ☐ 2 | ☐ 3 |
| 3. | faceți cumpărăturile? | ☐ 1 | ☐ 2 | ☐ 3 |
| 4. | pregătiți masa | ☐ 1 | ☐ 2 | ☐ 3 |
| 5. | faceți curățenie | ☐ 1 | ☐ 2 | ☐ 3 |
| 6. | organizați cheltuielile/ veniturile | ☐ 1 | ☐ 2 | ☐ 3 |

© 2014 Acest chestionar este protejat prin legea dreptului de autor

# Mobilitate

*Următoarele întrebări se referă la **mersul pe jos pe o distanță de aproximativ 1 kilometru.***

1. Puteți merge pe jos pe o distanță de 1 kilometru?
   - ☐1 Nu — *Mergeți la întrebarea 5 pe această pagină*
   - ☐2 Da

2. Vă este greu să mergeți pe jos 1 kilometru din cauza problemelor de sănătate sau altor probleme pe care le aveți?
   - ☐1 Nu, nu îmi este greu
   - ☐2 Da, îmi este greu

3. <u>În ultimele 12 luni</u> v-ați modificat modul în care mergeți pe jos pe o distanță de 1 kilometru ? *(de exemplu mergeți mai încet sau mai precaut, v-ați modificat postura sau pasul folosind un baston sau alt dispozitiv similar, sau oprindu-vă mai des decât obișnuiați).*
   - ☐1 Nu
   - ☐2 Da

4. <u>În ultimele 12 luni</u> ați mers pe jos mai rar pe o distanță de 1 kilometru ? *(de exemplu nu ați mai mers în fiecare zi, doar de 3-4 ori pe săptămână, poate probabil din cauză că obosiți mai ușor)*
   - ☐1 Nu
   - ☐2 Da

*Următoarele întrebări se referă la **urcatul a 10 trepte**.*

5. Puteți urca 10 trepte?
   - ☐1 Nu — *Mergeți la pagina 20 (Fumat)*
   - ☐2 Da

6. Vă este greu să urcați 10 trepte din cauza problemelor de sănătate sau altor **probleme** fizice?

☐1 Nu

☐2 Da

7. <u>În ultimele 12 luni</u> v-ați modificat modul în care urcați 10 trepte ?
*(de exemplu mergeți mai încet sau mai precaut, v-ați modificat tehnica, vă opriți mai des să vă sprijiniți de balustradă mai mult)*

☐1 Nu

☐2 Da

8. <u>În ultimele 12 luni</u> ați urcat mai rar 10 trepte ? *(de exemplu nu folosiți scările, de câte ori este posibil folosiți liftul, posibil din cauza durerilor articulare)*

☐1 Nu

☐2 Da

© 2014 Acest chestionar este protejat prin legea dreptului de autor 

# Fumat

1. <u>În prezent</u> sunteți fumător/fumătoare?
   - ☐1 Nu, nu am fumat niciodată
   - ☐2 Nu, am renunțat de mai mult de 6 luni
   - ☐3 Nu, am renunțat în ultimele 6 luni
   - ☐4 Da, în prezent sunt fumător/fumătoare

2. <u>În prezent</u> utilizați tutun în alte forme *(trabuc, pipă)*?
   - ☐1 Nu
   - ☐2 Da

*Următoarele întrebări sunt doar pentru fumători sau foști fumători.* ***Dacă nu ați fumat niciodată vă rugăm mergeți la pagina 22 (Consum de alcool).***

***Această secțiune este doar pentru FUMĂTORI.***

3. De câte ori <u>în ultimele 12 luni</u> nu ați fumat pentru cel puțin 24 de ore?
   - ☐1 Niciodată
   - ☐2 O dată
   - ☐3 De mai multe ori

4. V-ați gândit vreodată în mod serios să renunțați la fumat?
   - ☐1 Da, vreau să renunț în următoarele 30 de zile
   - ☐2 Da, vreau să renunț în următoarele 6 luni
   - ☐3 Nu, nu vreau să renunț la fumat

*Aceasta secțiune este doar pentru FOȘTI FUMĂTORI.*

5. De când ați renunțat la fumatul în mod regulat?
   - ☐ 1 De mai puțin de un an
   - ☐ 2 De mai mult de un an
   - ☐ 3 De mai mult de 5 ani
   - ☐ 4 De mai mult de 10 ani
   - ☐ 5 De mai mult de 15 ani

*Pentru FUMĂTORI și FOȘTI FUMĂTORI*

6. Ați făcut o echografie de aortă abdominală în ultimii 10 ani?
   - ☐ 1 Nu
   - ☐ 2 Da

# Consum de alcool

*Unitățile de alcool sunt definite astfel:*

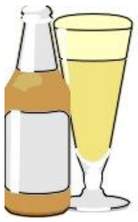

Jumătate de halbă de bere (250ml) = 1 unitate

sau

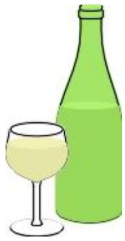

Un pahar mare de vin (175ml) = 2 unități

sau

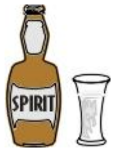


50ml băuturi spirtoase (tărie) = 1 unitate

sau

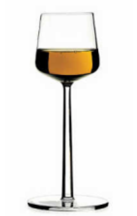

Un pahar mic de coniac/lichior = 1 unitate

*Următoarele întrebări sunt despre cât de des consumați alcool.*

1. Cât de des ați consumat alcool <u>în ultimul an</u>?

☐ 1 Niciodată — *Mergeți la pagina 24 (Activitate fizică)*

☐ 2 În fiecare lună sau mai rar

☐ 3 De 2-4 ori pe lună

☐ 4 De 2-3 ori pe săptămână

☐ 5 De 4 sau mai multe ori pe săptămână

2. Dacă vă gândiți la o zi dintr-o lună obișnuită <u>din ultimul an</u>, în care ați consumat alcool, câte unități ați consumat?

☐ 1 1 sau 2

☐ 2 3 sau 4

☐ 3 5 saur 6

☐ 4 7 - 9

☐ 5 10 sau mai multe

© 2014 Acest chestionar este protejat prin legea dreptului de autor 

3. Într-o lună obișnuită din ultimul an în câte ocazii s-a întâmplat să consumați 6 sau mai multe unități de alcool?

- ☐ 1 Niciodată
- ☐ 2 Mai puțin de o dată pe lună
- ☐ 3 O dată pe lună
- ☐ 4 O dată pe săptămână
- ☐ 5 Zilnic sau aproape zilnic

*Următoarele întrebări se referă la locul pe care consumul de alcool îl are în viața dvs.*

4. Ați avut vreodată senzația că ar trebui să reduceți cantitatea de alcool pe care o consumați?

- ☐ 1 Nu
- ☐ 2 Da

5. V-ați simțit deranjat/ă/enervat/ă când alții v-au criticat pentru consumul de alcool?

- ☐ 1 Nu
- ☐ 2 Da

6. V-ați simțit vinovat/ă pentru că beți alcool?

- ☐ 1 Nu
- ☐ 2 Da

7. Ați consumat vreodată alcool dimineața pentru a vă calma sau pentru a vă trece mahmureala?

- ☐ 1 Nu
- ☐ 2 Da

© 2014 Acest chestionar este protejat prin legea dreptului de autor

# Activitate fizică

*Următoarele întrebări se referă la activitatea dvs. fizică din ultimele 7 zile. Dacă ultimele 7 zile nu au fost obișnuite pentru dvs. , gândiți-vă la o săptămână tipică. Includeți activități pe care le desfășurați la servici, cu munca, în casă sau în grădină, atunci când vă deplasați dintr-o locație în alta, în timpul liber pe care îl petreceți făcând sport sau alte activități recreative. Toate aceste exemple se consideră activitate fizică.*

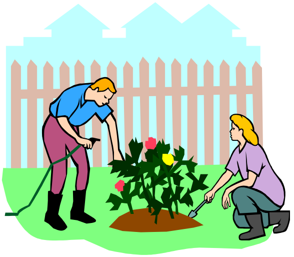
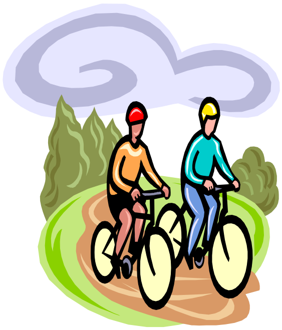
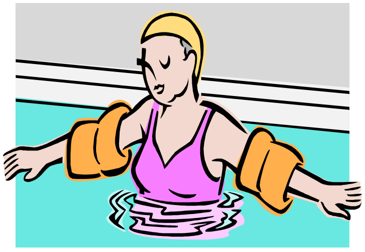
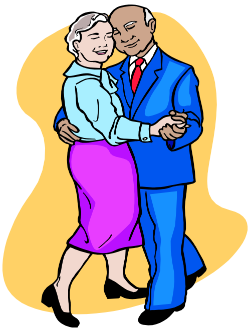

*Gândiți-vă la toate **activitățile fizice viguroase** pe care le-ați desfășurat în ultimele 7 zile. **Activitățile fizice viguroase** sunt cele care necesită un efort fizic intens și vă fac să respirați greu. Acestea includ munca intensă de orice fel, în casă sau în gradină (exemplu : săpat, activități recreative și sport cum este mersul pe bicicletă (inclusiv bicicletă fixă), alergat, aerobic sau fitness). Gândiți-vă doar la acele activități pe care le-ați făcut timp de **cel puțin 10 minute**.*

1. În ultimele 7 zile, în câte zile ați desfășurat **activități fizice viguroase**?

☐ 1 Niciodată *Mergeți la pagina 25 întrebarea 3*
☐ 2 1-2 zile pe săptămână
☐ 3 3-4 zile pe săptămână
☐ 4 5-6 zile pe săptămână
☐ 5 În fiecare zi

2. Cât timp ați petrecut de obicei desfășurând **activități fizice viguroase** într-una din acele zile?

☐ 1 Cam 10 minte pe zi ☐ 2 Cam 20 minte pe zi
☐ 3 Cam 30 minte pe zi ☐ 4 Cam 1 oră pe zi
☐ 5 Cam 2-3 ore pe zi ☐ 6 4 sau mai multe ore pe zi

© 2014 Acest chestionar este protejat prin legea dreptului de autor

*Acum gândiți-vă la **activitățile fizice moderate** pe care le-ați desfășurat <u>în ultimele 7 zile</u>. **Activitățile moderate** necesită un oarecare efort fizic și vă determină să respirați puțin mai greu decât de obicei. Activitățile fizice moderate pot fi cele desfășurate la servici, în casă sau gradină, în timpul liber petrecut cu activități recreative, făcând sport cum este mersul pe bicicletă cu viteză normală (inclusiv bicicletă fixă), mersul pe jos în pas alert, innotul sau dansul, alergat, aerobic sau fitness. Gândiți-vă doar la acele activități pe care le-ați făcut timp de **cel puțin 10 minute**.*

3. <u>În ultimele 7 zile</u>, în câte zile ați desfășurat **activități fizice moderate ?** Nu includeți mersul în pas normal.

- ☐ 1 Niciodată — *Mergeți la întrebarea 5 pe această pagină*
- ☐ 2 1-2 zile pe săptămână
- ☐ 3 3-4 zile pe săptămână
- ☐ 4 5-6 zile pe săptămână
- ☐ 5 În fiecare zi

4. Cât timp ați petrecut de obicei desfășurând **activități fizice moderate** într-<u>una din acele zile</u>?

- ☐ 1 Cam 10 minte pe zi
- ☐ 2 Cam 20 minte pe zi
- ☐ 3 Cam 30 minte pe zi
- ☐ 4 Cam 1 oră pe zi
- ☐ 5 Cam 2-3 ore pe zi
- ☐ 6 4 sau mai multe ore pe zi

*Gândiți-vă la timpul pe care l-ați petrecut **mergând pe jos** <u>în ultimele 7 zile</u>. Aceasta include mersul la servici sau acasă, plimbările pe jos dintr-un loc în altul sau mersul în timpul unor activități recreative sau sport.*

5. <u>În ultimele 7 zile</u>, în câte zile ați **mers pe jos** timp de cel puțin 10 minute?

- ☐ 1 Niciodată — *Mergeți la pagina 26 întrebarea 7*
- ☐ 2 1-2 zile pe săptămână
- ☐ 3 3-4 zile pe săptămână
- ☐ 4 5-6 zile pe săptămână
- ☐ 5 În fiecare zi

© 2014 Acest chestionar este protejat prin legea dreptului de autor

6. Cât timp ați petrecut **mergând pe jos** într-una din acele zile?

- ☐ 1 Cam 10 minte pe zi
- ☐ 2 Cam 20 minte pe zi
- ☐ 3 Cam 30 minte pe zi
- ☐ 4 Cam 1 oră pe
- ☐ 5 Cam 2-3 ore pe zi
- ☐ 6 4 sau mai multe ore pe zi

*Această întrebare se referă la timpul petrecând **stând** în ultimele 7 zile. Includeți timpul petrecut la servici, acasă, la un curs sau cu activități recreative. Includeți timpul petrecut stând la birou, cu prietenii, citind sau uitându-vă la televizor (și în pat).*

7. De obicei, în ultimele 7 zile într-o zi obișnuită, cât timp ați petrecut **stând așezat/ă** ?

- ☐ 1 Mai puțin de o oră pe zi
- ☐ 2 Cam 1-2 ore pe zi
- ☐ 3 Cam 3-4 ore pe zi
- ☐ 4 Mai mult de 4 ore pe zi

*În următoarea întrebare vrem să aflăm dacă v-ați dori să faceți mai multă activitate fizică, iar dacă nu, ce anume vă împiedică.*

8. Intenționați să faceți mai multă activitate fizică?

- ☐ 1 Nu, nu intențînez să fac mai multă activitate fizică
- ☐ 2 Da, intențînez să fac mai multă activitate fizică în următoarele 6 luni
- ☐ 3 Da, voi face mai multă activitate fizică în următoarea lună

9. Care dintre următoarele motive vă împiedică să fiți mai activ/ă fizic?

a. ☐ 1 Sunt deja activ/ă în mod frecvent
b. ☐ 1 Nu am companie pentru aceasta
c. ☐ 1 Nu știu nici o ocazie de a face activitate fizică potrivită vârstei mele
d. ☐ 1 Nu am timp
e. ☐ 1 Este scump
f. ☐ 1 Din cauza vremii
g. ☐ 1 Sufăr de o afecțiune care îmi limitează abilitatea de face activitate fizică
h. ☐ 1 Am o dizabilitate fizică
i. ☐ 1 Am dureri din ce în ce mai mari când sunt activ/ă fizic

© 2014 Acest chestionar este protejat prin legea dreptului de autor

# Nutriție și alimentație

*Următoarele întrebări sunt despre consumul dvs. de fructe și legume din ultima săptămână. Dacă săptămâna trecută nu a fost una obișnuită, gândiți-vă în trecut la o săptămână tipică. Exemplele de mai jos arată ce cantitate de fructe și legume înseamnă o porție.*

| 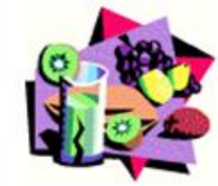 | 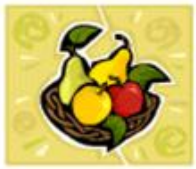 | 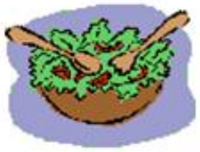 | 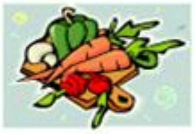 |
|---|---|---|---|
| **Suc de fructe sau legume**<br>200ml<br>neîndulcit | **Fructe și fructe de pădure**<br>cam 120g,<br>cam o mână plină | **Salată**<br>cam 120g,<br>un castron mic<br>sau farfurie | **Legume**<br>cam 120g,<br>un castron de supă,<br>sau jumătate de<br>cană de sos de rosii |

1. Cât de des în ultima săptămână ați băut o porție de **suc de fructe 100%** *(excluzând limonada, băuturi cu vitamine sau alte băuturi ce conțin doar parțial fructe )?* Numărați toate sucurile de fructe pe care le-ați băut în timpul meselor sau între mese..

- ☐ 1 Niciodată
- ☐ 2 O dată sau de două ori în ultima săptămână
- ☐ 3 3 – 6 ori pe săptămână
- ☐ 4 O dată pe zi
- ☐ 5 De două ori pe zi
- ☐ 6 De trei sau mai multe ori pe zi

2. Cât de des în ultima săptămână ați mâncat o porție de **fructe sau fructe de pădure?** *(**Nu includeți** suc de fructe)*. Includeți fructe proaspete, conservate sau congelate. Numărați porțiile de fructe sau fructe de pădure consumate la masă sau între mese.

- ☐ 1 Niciodată
- ☐ 2 O dată sau de două ori în ultima săptămână
- ☐ 3 3 – 6 ori pe săptămână
- ☐ 4 O dată pe zi
- ☐ 5 De două ori pe zi
- ☐ 6 De trei sau mai multe ori pe zi

© 2014 Acest chestionar este protejat prin legea dreptului de autor

3. Cât de des în ultima săptămână ați mâncat o porție de **salată** (*fără carne, pește, cartofi în ea)*?

- ☐ 1 Niciodată
- ☐ 2 O dată sau de două ori în ultima săptămână
- ☐ 3 3 – 6 ori pe săptămână
- ☐ 4 O dată pe zi
- ☐ 5 De două ori pe zi
- ☐ 6 De trei sau mai multe ori pe zi

4. Cât de des în ultima săptămână ați mâncat o porție de **legume** *(nu includeți salată sau cartofi)*? Includeți legume crude, gătite, conservate sau congelate.

- ☐ 1 Niciodată
- ☐ 2 O dată sau de două ori în ultima săptămână
- ☐ 3 3 – 6 ori pe săptămână
- ☐ 4 O dată pe zi
- ☐ 5 De două ori pe zi
- ☐ 6 De trei sau mai multe ori pe zi

5. Cât de des în ultima săptămână ați mâncat o porție de **supă de legume/ ciorbă de legume** ? **Includeți** supă de roșii, ciorbă de carne *(orice fel)* cu legume, sau alte supe de legume.

- ☐ 1 Niciodată
- ☐ 2 O dată sau de două ori în ultima săptămână
- ☐ 3 3 – 6 ori pe săptămână
- ☐ 4 O dată pe zi
- ☐ 5 De două ori pe zi
- ☐ 6 De trei sau mai multe ori pe zi

© 2014 Acest chestionar este protejat prin legea dreptului de autor 

6. Cât de des în ultima săptămână ați mâncat o porție de **sos de roșii** *(nu ketchup)*? *(Includeți sos de roșii pentru paste, cu pizza sau alte preparate)*.

- ☐1 Niciodată
- ☐2 O dată sau de două ori în ultima săptămână
- ☐3 3 – 6 ori pe săptămână
- ☐4 O dată pe zi
- ☐5 De două ori pe zi
- ☐6 De trei sau mai multe ori pe zi

7. Intenționați să includeți mai multe fructe și legume în dieta dvs.?

- ☐1 Nu, nu intenționez să mănânc să mănânc mai multe fructe și legume
- ☐2 Da, intenționez să mănânc să mănânc mai multe fructe și legume în următoarele 6 luni
- ☐3 Da, voi mânca mai multe fructe și legume luna viitoare

*Următoarele sunt întrebări generale despre nutriție și alimentație*.

8. Ați discutat despre nutriție/alimentație cu un medic sau nutriționist în ultimele 12 luni?

- ☐1 Nu
- ☐2 Da

9. În ultimele 3 luni ați mâncat mai puțin din cauza lipsei apetitului, a problemelor de digestie sau dificultăților la mestecat și înghițit?

- ☐1 Nu
- ☐2 Da

© 2014 Acest chestionar este protejat prin legea dreptului de autor

*Următoarele întrebări sunt despre consumul dvs. de **alimente bogate în grăsimi**.*

***Alegeți un singur răspuns pentru fiecare categorie.***

| Cât de des mâncați ... ? | | Niciodată sau mai puțin de o dată pe lună | De 1-3 ori pe lună | De 1-2 ori pe săptămână | De 3-4 ori pe săptămână | De 5 ori sau mai mult pe săptămână |
|---|---|---|---|---|---|---|
| 10. | Carne de vită sau porc prăjit/ friptură | ☐ 1 | ☐ 2 | ☐ 3 | ☐ 4 | ☐ 5 |
| 11. | Carne de pui prăjit cu tot cu piele, pește prăjit | ☐ 1 | ☐ 2 | ☐ 3 | ☐ 4 | ☐ 5 |
| 12. | Hamburger, cheeseburger, carne tocată | ☐ 1 | ☐ 2 | ☐ 3 | ☐ 4 | ☐ 5 |
| 13. | Cârnați | ☐ 1 | ☐ 2 | ☐ 3 | ☐ 4 | ☐ 5 |
| 14. | Brânză/preparate de brânză *(nu cele degresate)* | ☐ 1 | ☐ 2 | ☐ 3 | ☐ 4 | ☐ 5 |
| 15. | Lapte integral, lapte semidegresat, frișcă, smântână | ☐ 1 | ☐ 2 | ☐ 3 | ☐ 4 | ☐ 5 |
| 16. | Margarină, unt | ☐ 1 | ☐ 2 | ☐ 3 | ☐ 4 | ☐ 5 |
| 17. | Sosuri pentru salate, maioneză | ☐ 1 | ☐ 2 | ☐ 3 | ☐ 4 | ☐ 5 |
| 18. | Cartofi prăjiți sau chipsuri | ☐ 1 | ☐ 2 | ☐ 3 | ☐ 4 | ☐ 5 |
| 19. | Grisine/sticksuri, floricele de porumb/ biscuiți | ☐ 1 | ☐ 2 | ☐ 3 | ☐ 4 | ☐ 5 |
| 20. | Ciocolată, bomboane, biscuiți | ☐ 1 | ☐ 2 | ☐ 3 | ☐ 4 | ☐ 5 |
| 21. | Înghețată, gogoși, produse de patiserie, plăcinte, prăjituri, biscuiți, covrigi | ☐ 1 | ☐ 2 | ☐ 3 | ☐ 4 | ☐ 5 |

22. Intenționați să reduceți cantitatea de alimente bogate în grăsimi din dieta dvs.?

☐ 1 Nu, nu intenționez să mănânc mai puține alimente bogate în grăsimi

☐ 2 Da, intenționez să mănânc mai puține alimente bogate în grăsimi în următoarele 6 luni

☐ 3 Da, voi mânca mai puține alimente bogate în grăsimi în următoarea lună

© 2014 Acest chestionar este protejat prin legea dreptului de autor 

# Prevenția căderilor

1. Vă limitați activitatea deoarece vă este teamă că o să cădeți?

☐1 Nu
☐2 Da

2a. În ultimele 12 luni ați căzut pe jos?

☐1 Nu — *Mergeți la capitolul următor*
☐2 Da

2b. De câte ori ați căzut?

☐1 O dată
☐2 De mai multe ori

# Îngrijiri preventive

*Următoarele întrebări se referă la servicii medicale de care dvs. probabil ați beneficiat.* ***Vă rugăm să răspundeți fiecărei întrebări cu „NU" sau „DA"***

| **Ați avut...** | | **Nu** | **Da** |
|---|---|---|---|
| 1. | O măsurare a tensiunii arteriale în ultimul an ? | ☐1 | ☐2 |
| 2. | O determinare a glicemiei à jeun (dimineața pe nemâncate) în ultimii 3 ani ? | ☐1 | ☐2 |
| 3. | O determinare a colesterolului în ultimii 5 ani ? | ☐1 | ☐2 |
| 4. | Un consult oftalmologic în ultimii 2 ani ? | ☐1 | ☐2 |
| 5. | O evaluare a auzului în ultimul an ? | ☐1 | ☐2 |
| 6. | Vaccin antigripal în ultimul an ? | ☐1 | ☐2 |

© 2014 Acest chestionar este protejat prin legea dreptului de autor

# Despre dumneavoastră

1. Unde v-ați născut?
   - ☐1 România
   - ☐2 În altă țară

2. Care este limba dvs. maternă?
   - ☐1 Română
   - ☐2 Altă limbă

3. Cu cine locuiți în prezent *(alegeți doar **un singur răspuns** din următoarele)*?
   - ☐1 Locuiesc singur/ă
   - ☐2 Locuiesc cu soț/soție, partener/ă
   - ☐3 Locuiesc cu alt (alți) membri ai familiei
   - ☐4 Locuiesc cu un/o prieten/ă sau cu altcineva
   - ☐5 Altele

4. Unde locuiți?
   - ☐1 Apartament *(la parter sau am lift)*
   - ☐2 Apartament *(alte situații)*
   - ☐3 Casă

5. Ce educație aveți?
   - ☐1 Facultate
   - ☐2 Postliceală
   - ☐3 Liceu
   - ☐4 Școală gimnazială *(8 clase)*
   - ☐5 Școală primară *(4 clase)*
   - ☐6 Nu am școală *(analfabet)*

6. Ce ați muncit / unde ați lucrat cea mai mare parte din viață?

   ***Vă rugăm completați UN SINGUR răspuns***
   - ☐1 Angajat cu funcție de conducere
   - ☐2 Angajat fără funcție de conducere
   - ☐3 Ați avut propria afacere/ ocupație
   - ☐4 Casnic/ă

© 2014 Acest chestionar este protejat prin legea dreptului de autor

# Chestionar de Acceptabilitate

*Acest scurt chestionar vă întreabă despre experiența pe care ați avut-o completând chestionarul Profil de Sănătate pentru Persoane Vârstnice HRA-O. Răspunsurile dvs. sincere ne vor ajuta să îmbunătățim chestionarul HRA-O pentru a fi utilizat în viitor și pentru alte persoane.*

1. În general cât de dificil a fost pentru dvs. să înțelegeți întrebările și instrucțiunile din chestionar?

☐1 Foarte dificil ☐4 Destul de ușor

☐2 Destul de dificil ☐5 Foarte ușor

2. Au fost anumite întrebări sau capitole din chestionar care v-au iritat/supărat?

☐1 Nu ☐2 Da

**Dacă da:** Care au fost acestea?

---

3. Există anumite subiecte care **lipsesc** din acest chestionar și care considerați că ar trebui incluse?

☐1 Nu ☐2 Da

**Dacă da:** Care ar fi acestea?

---

4. Au fost anumite întrebări din chestionar neclare sau de neînțeles?

☐1 Nu ☐2 Da

**Dacă da:** Care au fost acestea?

---

*Vă rugăm să vă gândiți un moment și să verificați dacă sunt întrebări sau pagini pe care le-ați sărit.*

## Vă mulțumim pentru că ați completat acest chestionar!

© 2014 Geriatric Research Fund, Spital Netz Bern AG, Inselspital, PO Box 20, CH-3010 Bern, Switzerland. Acest chestionar este protejat prin legea dreptului de autor și nu poate fi (parțial sau total) reprodus sau utilizat în alte scopuri fără aprobarea prealabilă scrisă a deținătorilor dreptului de autor.

## Annex VI

## Abstraction form for health record information Romania

| | |
|---|---|
| Type of report | ☐1 begin of the study ☐2 end of the study |
| ID-number | |
| Study Group | ☐1 A Intervention ☐2B Placebo |
| Location | ☐1 Hospital ☐2 Out patient |
| First name | |
| Surname | |
| Date of examination | |

| | |
|---|---|
| Income | |
| Body weight | |
| ***Baseline only:*** Body height | |
| Systolic Blood pressure | |
| Diastolic Blood pressure | |
| Antihypertensive medication (name, dose) | |
| Antidiabetic medication (name, dose) | |
| Anticholesterol medication (name, dose) | |
| ***End of study only:*** Influenza vaccination 2014 (yes/ no) | |

## Annex VII

## Documentation of special events

| | |
|---|---|
| ID-number | |
| First name | |
| Surname | |
| Location | ☐$_1$ Hospital ☐$_2$Out patient |
| Study Group | ☐$_1$ A Intervention ☐$_2$B Placebo |

| | | |
|---|---|---|
| ☐$_1$ | Patient died (Full stop) | Date of death: ___________________ |
| ☐$_2$ | Patient withdraws informed consent (full stop) | Date if withdrawal: ___________________ |
| ☐$_2$ | Patient admitted to permanent nursing home (intervention is stopped, outcome data is collected) | Date of admission: ___________________ |
| ☐$_2$ | Patient is missing unknown reason<br>(cannot be reached by telephone or address) | |

**Annex VIII**

# 6 Month Follow-up Questionnaire Romania

*Name or stamp of Geriatrician*:

| | |
|---|---|
| ID-number | |
| Study Group | ☐1 A Intervention ☐2B Placebo |
| Location | ☐1 Hospital ☐2 Out patient |
| First name | |
| Surname | |
| Date of Birth (DD/MM/YY) | |
| Date of examination | |

*Please complete this Health and Well-being Questionnaire. Based on your answers, you will receive a report on your health and well-being and how to improve it.*

*Please do the following:*
- *Read each question carefully and follow the instructions.*
- *Put a cross in the relevant box for each answer, like this:* ☒
- *Do not write any comments in blank spaces or in the margins, they will not be considered.*
- *Answer the questions in each section even if the topic does not apply to you directly.*
- *Please check at the end that you have completed every page and every question on each page.*

© 2013 This questionnaire is protected by copyright

# Medications/Prescriptions

*The following questions ask about problems that you may have in taking medicines.* ***If <u>at the moment</u> you DO NOT take any medicines, go to page 3.***

1. Do you have a medication plan that lists your current medicines?
   - ☐ 1 No
   - ☐ 2 Yes

2. Do you use a 'Dosette box' (organiser for your pills) or blister pack?
   - ☐ 1 No
   - ☐ 2 Yes

3. Do you have difficulties in understanding when and how you should take your medicines?
   - ☐ 1 No
   - ☐ 2 Yes

4. Are you uncertain why you are taking any of your current medicines?
   - ☐ 1 No
   - ☐ 2 Yes

5. Are you prescribed medicines by more than one doctor?
   - ☐ 1 No
   - ☐ 2 Yes

6. Do you think that you are having any side-effects due to your medications?
   - ☐ 1 No
   - ☐ 2 Yes

© 2013 This questionnaire is protected by copyright

# Psychosocial Health & Well Being

*These questions are about how you feel and how things have been with you <u>during the past month</u>.*

| **How much of the time, <u>during the last month</u>, have you …** | | **None of the time** | **A little of the time** | **Some of the time** | **A good bit of the time** | **Most of the time** | **All of the time** |
|---|---|---|---|---|---|---|---|
| 1. | ... felt calm and peaceful? | ☐1 | ☐2 | ☐3 | ☐4 | ☐5 | ☐6 |
| 2. | ... been a very nervous person? | ☐1 | ☐2 | ☐3 | ☐4 | ☐5 | ☐6 |
| 3. | ... felt so down in the dumps that nothing could cheer you up? | ☐1 | ☐2 | ☐3 | ☐4 | ☐5 | ☐6 |
| 4. | ... felt downhearted and low? | ☐1 | ☐2 | ☐3 | ☐4 | ☐5 | ☐6 |
| 5. | ... been a happy person? | ☐1 | ☐2 | ☐3 | ☐4 | ☐5 | ☐6 |

© 2013 This questionnaire is protected by copyright

# Social Network

*People sometimes look to others for friendship, assistance, or other types of support.*

*The following three questions are about your family members, including your spouse, in-laws, and any other relatives.*

1. How many relatives or family members do you see or hear from at least once a month? (Note: Include spouse, in-laws, and any other relatives.)

| | |
|---|---|
| ☐1 Nine or more | ☐4 Two |
| ☐2 Five to eight | ☐5 One |
| ☐3 Three or four | ☐6 None |

2. How many relatives or family members do you feel close to that you can call on for help? (Note: Include spouse, in-laws, and any other relatives.)

| | |
|---|---|
| ☐1 Nine or more | ☐4 Two |
| ☐2 Five to eight | ☐5 One |
| ☐3 Three or four | ☐6 None |

3. With how many relatives or family members can you comfortably discuss private matters? (Note: Include spouse, in-laws, and any other relatives.)

| | |
|---|---|
| ☐1 Nine or more | ☐4 Two |
| ☐2 Five to eight | ☐5 One |
| ☐3 Three or four | ☐6 None |

*The following three questions are about contacts with friends or neighbours.*

4. How many friends/neighbours do you see or hear from at least once a month?

| | |
|---|---|
| ☐1 Nine or more | ☐4 Two |
| ☐2 Five to eight | ☐5 One |
| ☐3 Three or four | ☐6 None |

5. How many friends/neighbours do you feel close to that you can call on for help?

| | |
|---|---|
| ☐1 Nine or more | ☐4 Two |
| ☐2 Five to eight | ☐5 One |
| ☐3 Three or four | ☐6 None |

6. With how many friends/neighbours can you comfortably discuss private matters?

| | |
|---|---|
| ☐1 Nine or more | ☐4 Two |
| ☐2 Five to eight | ☐5 One |
| ☐3 Three or four | ☐6 None |

© 2013 This questionnaire is protected by copyright

*The next questions are about **social activities**.*

7. How often each month do you take part in organised activities (for example hobby or leisure groups, clubs, religious or political associations)?

   - ☐1 Nine times or more
   - ☐2 Between 5 and 8 times
   - ☐3 3 or 4 times
   - ☐4 Twice
   - ☐5 Once
   - ☐6 Never

8. How often do you help others, for example, if they are unwell, doing housework, providing meals, running errands, taking people to hospital, providing child care?

   - ☐1 Never
   - ☐2 Less than once a month
   - ☐3 Once or twice a month
   - ☐4 Once or more a week
   - ☐5 Daily

© 2013 This questionnaire is protected by copyright

# Tobacco Use

1. Are you currently a cigarette smoker?
   - ☐ 1 No, I have never smoked cigarettes
   - ☐ 2 No, I stopped more than 6 months ago
   - ☐ 3 No, I stopped in the last 6 months
   - ☐ 4 Yes, I am currently a cigarette smoker

2. Do you presently use tobacco in other forms (cigar or pipe)?
   - ☐ 1 No
   - ☐ 2 Yes

© 2013 This questionnaire is protected by copyright

# Alcohol Use

*Units of alcohol are counted as follows:*

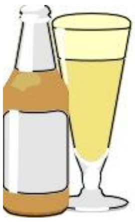

Half a pint (250ml) of medium strength beer = 1 unit

or

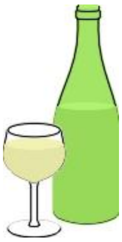

Medium size (175ml) wine glass = 2 units

or

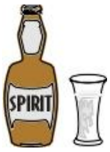


A single pub measure of spirits = 1 unit

or

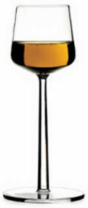

A small glass of sherry = 1 unit

*The next questions ask about how often you drink alcohol.*

1. In the last year how often have you drunk alcohol?
   - ☐1 Never → *Skip to page 8 (Physical Activity)*
   - ☐2 Monthly or less
   - ☐3 Two to four times a month
   - ☐4 Two to three times a week
   - ☐5 Four or more times a week

2. Thinking about a day in a typical month during the last year, when you did drink alcohol, how many units did you have (on average)?
   - ☐1 1 or 2
   - ☐2 3 or 4
   - ☐3 5 or 6
   - ☐4 7 to 9
   - ☐5 10 or more

3. In a typical month in the last year, on how many occasions did you have 6 or more units of alcohol?
   - ☐1 None
   - ☐2 Less than once a month
   - ☐3 Once a month
   - ☐4 Once a week
   - ☐5 Daily or almost daily

© 2013 This questionnaire is protected by copyright

# Physical Activity

*The following questions are about your physically activity <u>in the last 7 days</u>. If the last seven days were not typical for you, think of a more typical week. Include activities you do at work, as part of your housework and gardening, to get from place to place, and in your spare time for leisure, exercise or sport. All the examples shown count as physical activity.*

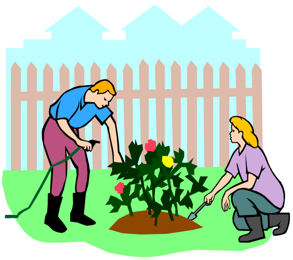
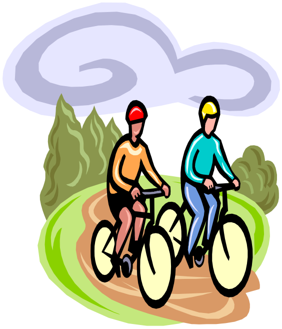
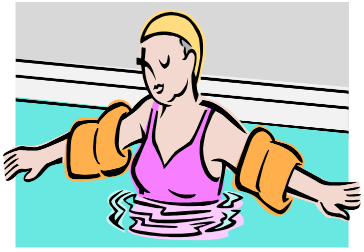
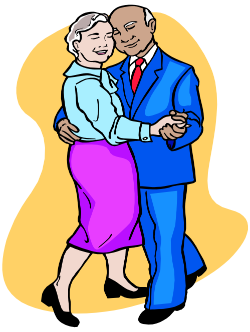

*Think about all the* ***vigorous physical activities*** *that you did <u>in the last 7 days</u>.* ***Vigorous activities*** *take hard physical effort and make you breathe much harder than normal. This includes heavy work of any sort, including in and around the home or outdoors such as heavy gardening (eg digging), leisure activities and sports like fast cycling (including on an exercise bike), jogging, aerobics or fitness training. Think only about those physical activities that you did for* ***at least 10 minutes*** *at a time.*

1. <u>During the last 7 days</u>, on how many days did you do **vigorous physical activities**?

   - ☐1 Not at all → *Skip to page 9 question 3*
   - ☐2 1 or 2 days a week
   - ☐3 3 or 4 days a week
   - ☐4 5 to 6 days a week
   - ☐5 Everyday

2. How much time did you typically spend doing **vigorous physical activities** on <u>one of those days</u>?

   - ☐1 About 10 minutes a day
   - ☐2 About 20 minutes a day
   - ☐3 About 30 minutes a day
   - ☐4 About an hour a day
   - ☐5 About 2 – 3 hours a day
   - ☐6 4 or more hours a day

© 2013 This questionnaire is protected by copyright

*Now, think about all the **moderate physical activities** that you did in the last 7 days. **Moderate activities** take some physical effort and make you breathe a bit harder than normal. Moderately vigorous activities can be done at work, in and around the house, in leisure time or sports like cycling at normal speed, brisk walking, swimming or dancing. Think only about those physical activities that you did for **at least 10 minutes** at a time.*

3. During the last 7 days, on how many days did you do **moderate physical activities**? Do not include walking at a normal pace?
   - ☐1 Not at all → *Skip to question 5 on this page*
   - ☐2 1 or 2 days a week
   - ☐3 3 or 4 days a week
   - ☐4 5 to 6 days a week
   - ☐5 Everyday

4. How much time did you typically spend doing **moderate physical activities** on one of those days?
   - ☐1 About 10 minutes a day
   - ☐2 About 20 minutes a day
   - ☐3 About 30 minutes a day
   - ☐4 About an hour a day
   - ☐5 About 2 – 3 hours a day
   - ☐6 4 or more hours a day

*Think about the time you spent **walking** in the last 7 days. This includes at work and at home, walking to travel from place to place, and any other walking that you might do solely for sport, exercise or leisure.*

5. During the last 7 days, on how many days did you **walk** for at least 10 minutes at a time?
   - ☐1 Not at all → *Skip to page 10 question 7*
   - ☐2 1 or 2 days a week
   - ☐3 3 or 4 days a week
   - ☐4 5 to 6 days a week
   - ☐5 Everyday

6. How much time did you usually spend **walking** on one of those days?
   - ☐1 About 10 minutes a day
   - ☐2 About 20 minutes a day
   - ☐3 About 30 minutes a day
   - ☐4 About an hour a day
   - ☐5 About 2 – 3 hours a day
   - ☐6 4 or more hours a day

© 2013 This questionnaire is protected by copyright

*The last question is about the time you spent **sitting** <u>during the last 7 days</u>. Include time spent at work, at home, during a course or during leisure time. This may include time spent sitting at a desk, with friends, reading, or sitting (or lying down) to watch television.*

7. During the <u>last 7 days</u>, how much time did you spend **sitting** on a typical day?

    ☐1 Less than one hour a day
    ☐2 About one to two hours a day
    ☐3 About three to four hours a day
    ☐4 More than four hours a day

© 2013 This questionnaire is protected by copyright

# Nutrition

*The next questions are about your consumption of **fruit and vegetables** in the last week. If the last week was not a typical one, think back to a typical week. The examples below show how much fruit and vegetables make up a portion.*

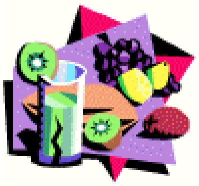

**Fruit or vegetable juice**
200ml of unsweetened juice

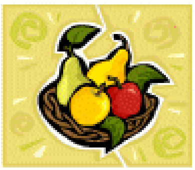

**Fruit and berries**
about 120g, about one handful

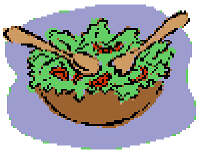

**Salad**
about 120g, a small plate/bowl

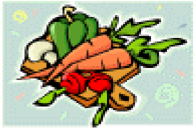

**Vegetables**
about 120g, or one bowl of soup, or half a cup of tomato sauce

1. How often in the last week have you drunk a portion of **100% pure fruit juice** (***excluding*** *lemonade, vitamin drinks or other drinks that only contain some fruit)? Count all the fruit juices that you have had at or between main meals.*
   - ☐1 None in the last week
   - ☐2 Once or twice in the last week
   - ☐3 Three to six times per week
   - ☐4 Once a day
   - ☐5 Twice a day
   - ☐6 Three or more times a day

2. How often have you eaten a portion of **fruit or berries** in the last week? (***Not counting*** *fruit juice) Include fresh, tinned and frozen fruits. Count all fruit and berries, eaten at or between main meals*
   - ☐1 None in the last week
   - ☐2 Once or twice in the last week
   - ☐3 Three to six times per week
   - ☐4 Once a day
   - ☐5 Twice a day
   - ☐6 Three or more times a day

3. How often in the last week have you eaten a portion of **salad** (***without*** *sausage, cheese, potato or tuna in it)?*
   - ☐1 None in the last week
   - ☐2 Once or twice in the last week
   - ☐3 Three to six times per week
   - ☐4 Once a day
   - ☐5 Twice a day
   - ☐6 Three or more times a day

© 2013 This questionnaire is protected by copyright

4. How often in the last week have you eaten a portion of **vegetables** *(**not including** salad or potatoes)? Count raw, cooked, tinned and frozen vegetables.*

- ☐1 None in the last week
- ☐2 Once or twice in the last week
- ☐3 Three to six times per week
- ☐4 Once a day
- ☐5 Twice a day
- ☐6 Three or more times a day

5. How often in the last week have you eaten a portion of **vegetable soup**? *(**Include** tomato soup, meat soup with vegetables, minestrone or other vegetable soups)*

- ☐1 None in the last week
- ☐2 Once or twice in the last week
- ☐3 Three to six times per week
- ☐4 Once a day
- ☐5 Twice a day
- ☐6 Three or more times a day

6. How often in the last week have you eaten a portion of **tomato sauce (not ketchup)**? *(**Include** tomato sauce eaten with pasta, on a pizza or in any other dish)*

- ☐1 None in the last week
- ☐2 Once or twice in the last week
- ☐3 Three to six times per week
- ☐4 Once a day
- ☐5 Twice a day
- ☐6 Three or more times a day

© 2013 This questionnaire is protected by copyright

*The following questions are about your consumption of **high-fat foods.***
***Tick ONE answer for each food category.***

| How often do you eat . . .? | | Never or less than once per MONTHS | 1-3 times per MONTHS | 1-2 times per WEEK | 3-4 times per WEEK | 5 times or more per WEEK |
|---|---|---|---|---|---|---|
| 1. | Beef or pork, such as steak, roasts, chops | ☐1 | ☐2 | ☐3 | ☐4 | ☐5 |
| 2. | Fried chicken with skin, fried fish | ☐1 | ☐2 | ☐3 | ☐4 | ☐5 |
| 3. | Hamburgers, cheeseburgers, minced beef | ☐1 | ☐2 | ☐3 | ☐4 | ☐5 |
| 4. | Sausages | ☐1 | ☐2 | ☐3 | ☐4 | ☐5 |
| 5. | Cheese spread or cheese (not low-fat) | ☐1 | ☐2 | ☐3 | ☐4 | ☐5 |
| 6. | Whole milk, semi-skimmed milk or cream | ☐1 | ☐2 | ☐3 | ☐4 | ☐5 |
| 7. | Margarine, butter or gravy | ☐1 | ☐2 | ☐3 | ☐4 | ☐5 |
| 8. | Salad cream (not diet) or mayonnaise | ☐1 | ☐2 | ☐3 | ☐4 | ☐5 |
| 9. | French fries, fried potatoes, or chips | ☐1 | ☐2 | ☐3 | ☐4 | ☐5 |
| 10. | Crisps, popcorn, cracker biscuits | ☐1 | ☐2 | ☐3 | ☐4 | ☐5 |
| 11. | Chocolates, sweets, biscuits | ☐1 | ☐2 | ☐3 | ☐4 | ☐5 |
| 12. | Ice cream (not low-fat), doughnuts, pastries, pies, cake, cookies, biscuits | ☐1 | ☐2 | ☐3 | ☐4 | ☐5 |

© 2013 This questionnaire is protected by copyright

# Falls Prevention

1. Do you limit your activities because you are afraid you will fall?

☐1 No
☐2 Yes

# Preventive Care

*The next questions are about health care services that you may have had.* ***Please answer each question with NO or YES.***

| **Have you had a ...** | | **No** | **Yes** |
|---|---|---|---|
| 1. | Eyesight checkup within the past 6 months? | ☐1 | ☐2 |
| 2. | Hearing checkup within the past 6 months? | ☐1 | ☐2 |
| 3. | Immunisation against influenza ("flu") within the past 6 months? | ☐1 | ☐2 |

**Thank you for completing this questionnaire!**

© 2013 Geriatric Research Fund, Spital Netz Bern AG, Inselspital, PO Box 20, CH-3010 Bern, Switzerland. This questionnaire is protected by copyright and may not (as a whole or in part) be reproduced or used for other purposes without prior written permission of the copyright owners.

# Profil Chestionar Final al Stării de Sănătate HRA-O

*Va rugăm să nu completați acest tabel.*

| | |
|---|---|
| ID-number | ☐☐☐☐☐☐☐☐☐☐☐☐☐☐☐☐☐☐ |
| Study Group | ☐ 1 A Intervention ☐ 2 B Placebo |
| Location | ☐ 1 Hospital ☐ 2 Out patient |
| First name | ☐☐☐☐☐☐☐☐☐☐☐☐☐☐☐☐☐☐ |
| Surname | ☐☐☐☐☐☐☐☐☐☐☐☐☐☐☐☐☐☐ |
| Date of examination | Day ☐☐ Month ☐☐ Year ☐☐☐☐ |

*Vă rugăm să completați acest chestionar.*

*Vă rugăm să:*

- *Citiți fiecare întrebare cu atenție și urmăriți instrucțiunile.*
- *Puneți un X în pătratul relevant pentru fiecare întrebare astfel:* ☒
- *Nu scrieți comentarii în spațiile libere sau pe margini, nu vor fi luate în considerare.*
- *Răspundeți întrebărilor de la fiecare capitol, chiar dacă subiectul respectiv nu se aplică în cazul dvs.*
- *La sfârșit verificați dacă ați completat fiecare pagină și dacă ați răspuns la toate întrebările de pe fiecare pagină.*

© 2014 Acest chestionar este protejat prin legea dreptului de autor

# Medicamente/Rețete

*Următoarele întrebări sunt despre eventualele probleme sau dificultăți pe care le-ați putea avea atunci când luați medicamente*

***Dacă <u>în prezent</u> NU luați nici un medicament, mergeți la pagina 3.***

1. Aveți o listă și un plan *(schemă)* cu medicamentele pe care trebuie să le luați?

☐1 Nu

☐2 Da

2. Folosiți un organizator *(cutie)* de medicamente?

☐1 Nu

☐2 Da

3. Întâmpinați dificultăți în a înțelege când și cum trebuie să vă luați medicamentele?

☐1 Nu

☐2 Da

4. Există vreun medicament pe care nu sunteți sigur/ă de ce îl luați?

☐1 Nu

☐2 Da

6. Credeți că suferiți vreo reacție adversă sau simptome cauzate de medicamentele pe care le luați?

☐1 Nu

☐2 Da

© 2014 Acest chestionar este protejat prin legea dreptului de autor

# Starea de sănătate psihosocială și starea de bine

*Aceste întrebări sunt despre cum v-ați simțit și cum v-a mers **în ultima lună**.*

| Cât de des în ultima lună... | | Niciodată | Rareori | Câteodată | Destul de des | Aproape tot timpul | Tot timpul |
|---|---|---|---|---|---|---|---|
| 1. | ... v-ați simțit calm și împăcat? | ☐ 1 | ☐ 2 | ☐ 3 | ☐ 4 | ☐ 5 | ☐ 6 |
| 2. | ... ați fost nervos /nervoasă? | ☐ 1 | ☐ 2 | ☐ 3 | ☐ 4 | ☐ 5 | ☐ 6 |
| 3. | ... v-ați simțit trist și deprimat și nimic nu v-a putut înveseli? | ☐ 1 | ☐ 2 | ☐ 3 | ☐ 4 | ☐ 5 | ☐ 6 |
| 4. | ... v-ați simțit descurajat și fără energie? | ☐ 1 | ☐ 2 | ☐ 3 | ☐ 4 | ☐ 5 | ☐ 6 |
| 5. | ... v-ați simțit fericit/ă? | ☐ 1 | ☐ 2 | ☐ 3 | ☐ 4 | ☐ 5 | ☐ 6 |

# Rețea socială

*Uneori oamenii privesc către ceilalți căutând prieteni, suport sau alt fel de ajutor.*

*Următoarele întrebare sunt despre familia dvs. , inclusiv soț/soție, socri și alte rude.*

1. Cu câți dintre membrii familiei sau alte rude vorbiți sau vă întâlniți cel puțin o dată pe lună ?
   *(Notă: inclusiv soț/soție, socri sau oricare alte rude.)*

| | | | |
|---|---|---|---|
| ☐ 1 | 9 sau mai mulți | ☐ 4 | 2 |
| ☐ 2 | 5 sau 8 | ☐ 5 | 1 |
| ☐ 3 | 3 sau 4 | ☐ 6 | nici unul |

© 2014 Acest chestionar este protejat prin legea dreptului de autor

*Următoarele întrebare se referă la contactele pe care dvs. le aveți cu prietenii sau vecinii.*

4. Cu câți prieteni/vecini vorbiți sau vă întâlniți cel puțin o dată pe lună?

- ☐ 1 9 sau mai mulți
- ☐ 2 5 sau 8
- ☐ 3 3 sau 4
- ☐ 4 2
- ☐ 5 1
- ☐ 6 nici unul

*Următoarele întrebări sunt despre* ***activitățile sociale***..

11. Cât de des în fiecare lună luați parte la activități organizate *(de exemplu : grupuri de lectură, cluburi, asociații politice sau religioase)*?

- ☐ 1 9 sau mai multe ori
- ☐ 2 5 sau 8 ori
- ☐ 3 3 sau 4 ori
- ☐ 4 2 ori
- ☐ 5 1 dată
- ☐ 6 niciodată

12. Cât de des îi ajutați pe alții, atunci când nu se simt bine, de exemplu cu treburile casei, să le duceți de mâncare, să faceți comisioane, să-i duceți la medic sau să-i ajutați cu copiii?

- ☐ 1 Niciodată
- ☐ 2 Mai puțin de o dată pe lună
- ☐ 3 O dată sau de două ori pe lună
- ☐ 4 O dată sau de două ori pe săptămână
- ☐ 5 În fiecare zi

© 2014 Acest chestionar este protejat prin legea dreptului de autor

# Fumat

1. În prezent sunteți fumător/fumătoare?
   - ☐ 1 Nu, nu am fumat niciodată
   - ☐ 2 Nu, am renunțat de mai mult de 6 luni
   - ☐ 3 Nu, am renunțat în ultimele 6 luni
   - ☐ 4 Da, în prezent sunt fumător/fumătoare

2. În prezent utilizați tutun în alte forme *(trabuc, pipă)*?
   - ☐ 1 Nu
   - ☐ 2 Da

3. De câte ori în ultimele 6 luni nu ați fumat pentru cel puțin 24 de ore?
   - ☐ 1 Niciodată
   - ☐ 2 O dată
   - ☐ 3 De mai multe ori

© 2014 Acest chestionar este protejat prin legea dreptului de autor

# Consum de alcool

*Unitățile de alcool sunt definite astfel:*

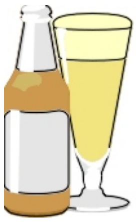

Jumătate de halbă de bere (250ml) = 1 unitate

sau

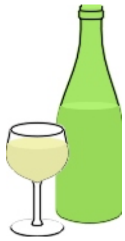

Un pahar mare de vin (175ml) = 2 unități

sau

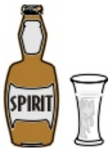


50ml băuturi spirtoase (tărie) = 1 unitate

sau

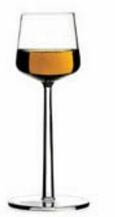

Un pahar mic de coniac/lichior = 1 unitate

*Următoarele întrebări sunt despre cât de des consumați alcool.*

1. Cât de des ați consumat alcool în ultimele 6 luni?

- ☐ 1 Niciodată → *Mergeți la pagina 8 (Activitate fizică)*
- ☐ 2 În fiecare lună sau mai rar
- ☐ 3 De 2-4 ori pe lună
- ☐ 4 De 2-3 ori pe săptămână
- ☐ 5 De 4 sau mai multe ori pe săptămână

2. Dacă vă gândiți la o zi dintr-o lună obișnuită din ultimele 6 luni, în care ați consumat alcool, câte unități ați consumat?

- ☐ 1 1 sau 2
- ☐ 2 3 sau 4
- ☐ 3 5 saur 6
- ☐ 4 7 - 9
- ☐ 5 10 sau mai multe

© 2014 Acest chestionar este protejat prin legea dreptului de autor

3. Într-o lună obișnuită din ultimele 6 luni în câte ocazii s-a întâmplat să consumați 6 sau mai multe unități de alcool ?

- ☐ 1 Niciodată
- ☐ 2 Mai puțin de o dată pe lună
- ☐ 3 O dată pe lună
- ☐ 4 O dată pe săptămână
- ☐ 5 Zilnic sau aproape zilnic

© 2014 Acest chestionar este protejat prin legea dreptului de autor 

# Activitate fizică

*Următoarele întrebări se referă la activitatea dvs. fizică din ultimele 7 zile. Dacă ultimele 7 zile nu au fost obișnuite pentru dvs. , gândiți-vă la o săptămână tipică.  Includeți activități pe care le desfășurați la servici, cu munca, în casă sau în grădină, atunci când vă deplasați dintr-o locație în alta, în timpul liber pe care îl petreceți făcând sport sau alte activități recreative. Toate aceste exemple se consideră activitate fizică.*

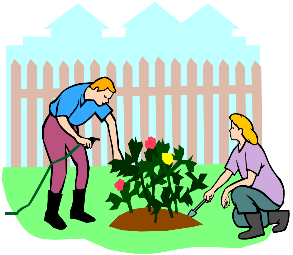
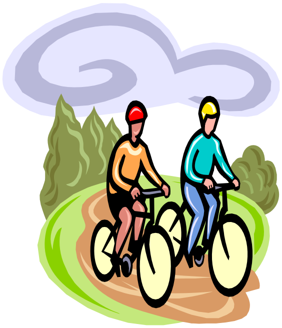
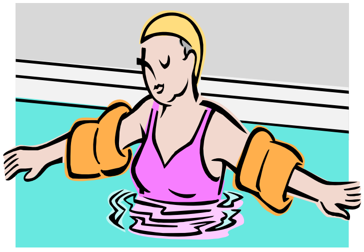
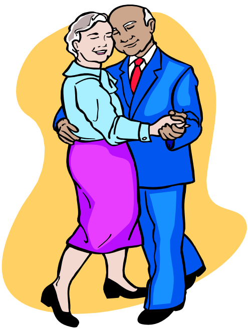

*Gândiți-vă la toate **activitățile fizice viguroase** pe care le-ați desfășurat în ultimele 7 zile. **Activitățile fizice viguroase** sunt cele care necesită un efort fizic intens și vă fac să respirați greu. Acestea includ munca intensă de orice fel, în casă sau în gradină (exemplu : săpat, activități recreative și sport cum este mersul pe bicicletă (inclusiv bicicletă fixă), alergat, aerobic sau fitness). Gândiți-vă doar la acele activități pe care le-ați făcut timp de **cel puțin 10 minute**.*

1. În ultimele 7 zile, în câte zile ați desfășurat **activități fizice viguroase**?

☐ 1 Niciodată → *Mergeți la pagina 9 întrebarea 3*
☐ 2 1-2 zile pe săptămână
☐ 3 3-4 zile pe săptămână
☐ 4 5-6 zile pe săptămână
☐ 5 În fiecare zi

2. Cât timp ați petrecut de obicei desfășurând **activități fizice viguroase** într-una din acele zile?

☐ 1 Cam 10 minte pe zi
☐ 2 Cam 20 minte pe zi
☐ 3 Cam 30 minte pe zi
☐ 4 Cam 1 oră pe zi
☐ 5 Cam 2-3 ore pe zi
☐ 6 4 sau mai multe ore pe zi

© 2014 Acest chestionar este protejat prin legea dreptului de autor

*Acum gândiți-vă la **activitățile fizice moderate** pe care le-ați desfășurat în ultimele 7 zile. **Activitățile moderate** necesită un oarecare efort fizic și vă determină să respirați puțin mai greu decât de obicei. Activitățile fizice moderate pot fi cele desfășurate la servici, în casă sau gradină, în timpul liber petrecut cu activități recreative, făcând sport cum este mersul pe bicicletă cu viteză normală (inclusiv bicicletă fixă), mersul pe jos în pas alert, innotul sau dansul, alergat, aerobic sau fitness. Gândiți-vă doar la acele activități pe care le-ați făcut timp de **cel puțin 10 minute**.*

3. În ultimele 7 zile, în câte zile ați desfășurat **activități fizice moderate ?** Nu includeți mersul în pas normal.

- ☐ 1 Niciodată ⟶ *Mergeți la întrebarea 5 pe această pagină*
- ☐ 2 1-2 zile pe săptămână
- ☐ 3 3-4 zile pe săptămână
- ☐ 4 5-6 zile pe săptămână
- ☐ 5 În fiecare zi

4. Cât timp ați petrecut de obicei desfășurând **activități fizice moderate** într-una din acele zile?

| | |
|---|---|
| ☐ 1 Cam 10 minte pe zi | ☐ 2 Cam 20 minte pe zi |
| ☐ 3 Cam 30 minte pe zi | ☐ 4 Cam 1 oră pe zi |
| ☐ 5 Cam 2-3 ore pe zi | ☐ 6 4 sau mai multe ore pe zi |

*Gândiți-vă la timpul pe care l-ați petrecut **mergând pe jos** în ultimele 7 zile. Aceasta include mersul la servici sau acasă, plimbările pe jos dintr-un loc în altul sau mersul în timpul unor activități recreative sau sport.*

5. În ultimele 7 zile, în câte zile ați **mers pe jos** timp de cel puțin 10 minute?

- ☐ 1 Niciodată ⟶ *Mergeți la pagina 10 întrebarea 7*
- ☐ 2 1-2 zile pe săptămână
- ☐ 3 3-4 zile pe săptămână
- ☐ 4 5-6 zile pe săptămână
- ☐ 5 În fiecare zi

© 2014 Acest chestionar este protejat prin legea dreptului de autor

6. Cât timp ați petrecut **mergând pe jos** într-una din acele zile?

- ☐ 1 Cam 10 minte pe zi
- ☐ 2 Cam 20 minte pe zi
- ☐ 3 Cam 30 minte pe zi
- ☐ 4 Cam 1 oră pe
- ☐ 5 Cam 2-3 ore pe zi
- ☐ 6 4 sau mai multe ore pe zi

*Această întrebare se referă la timpul petrecând **stând** în ultimele 7 zile. Includeți timpul petrecut la servici, acasă, la un curs sau cu activități recreative. Includeți timpul petrecut stând la birou, cu prietenii, citind sau uitându-vă la televizor (și în pat).*

7. De obicei, în ultimele 7 zile într-o zi obișnuită, cât timp ați petrecut **stând așezat/ă** ?

- ☐ 1 Mai puțin de o oră pe zi
- ☐ 2 Cam 1-2 ore pe zi
- ☐ 3 Cam 3-4 ore pe zi
- ☐ 4 Mai mult de 4 ore pe zi

# Nutriție și alimentație

*Următoarele întrebări sunt despre consumul dvs. de fructe și legume din ultima săptămână. Dacă săptămâna trecută nu a fost una obișnuită, gândiți-vă în trecut la o săptămână tipică. Exemplele de mai jos arată ce cantitate de fructe și legume înseamnă o porție.*

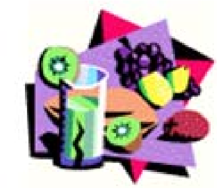

**Suc de fructe sau legume**
200ml
neîndulcit

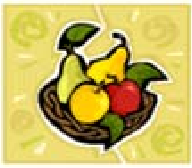

**Fructe și fructe de pădure**
cam 120g,
cam o mână plină

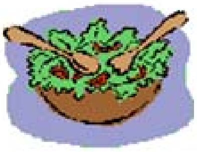

**Salată**
cam 120g,
un castron mic
sau farfurie

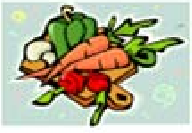

**Legume**
cam 120g,
un castron de supă,
sau jumătate de
cană de sos de rosii

1. Cât de des în ultima săptămână ați băut o porție de **suc de fructe 100%** *(**excluzând** limonada, băuturi cu vitamine sau alte băuturi ce conțin doar parțial fructe )?* Numărați toate sucurile de fructe pe care le-ați băut în timpul meselor sau între mese..

☐ 1 Niciodată
☐ 2 O dată sau de două ori în ultima săptămână
☐ 3 3 – 6 ori pe săptămână
☐ 4 O dată pe zi
☐ 5 De două ori pe zi
☐ 6 De trei sau mai multe ori pe zi

2. Cât de des în ultima săptămână ați mâncat o porție de **fructe sau fructe de pădure?** *(**Nu includeți** suc de fructe)*. Includeți fructe proaspete, conservate sau congelate. Numărați porțiile de fructe sau fructe de pădure consumate la masă sau între mese.

☐ 1 Niciodată
☐ 2 O dată sau de două ori în ultima săptămână
☐ 3 3 – 6 ori pe săptămână
☐ 4 O dată pe zi
☐ 5 De două ori pe zi
☐ 6 De trei sau mai multe ori pe zi

© 2014 Acest chestionar este protejat prin legea dreptului de autor

3. Cât de des în ultima săptămână ați mâncat o porție de **salată** (*fără carne, pește, cartofi în ea)*?

- ☐ 1 Niciodată
- ☐ 2 O dată sau de două ori în ultima săptămână
- ☐ 3 3 – 6 ori pe săptămână
- ☐ 4 O dată pe zi
- ☐ 5 De două ori pe zi
- ☐ 6 De trei sau mai multe ori pe zi

4. Cât de des în ultima săptămână ați mâncat o porție de **legume** *(nu includeți salată sau cartofi)*? Includeți legume crude, gătite, conservate sau congelate.

- ☐ 1 Niciodată
- ☐ 2 O dată sau de două ori în ultima săptămână
- ☐ 3 3 – 6 ori pe săptămână
- ☐ 4 O dată pe zi
- ☐ 5 De două ori pe zi
- ☐ 6 De trei sau mai multe ori pe zi

5. Cât de des în ultima săptămână ați mâncat o porție de **supă de legume/ ciorbă de legume** ? **Includeți** supă de roșii, ciorbă de carne *(orice fel)* cu legume, sau alte supe de legume.

- ☐ 1 Niciodată
- ☐ 2 O dată sau de două ori în ultima săptămână
- ☐ 3 3 – 6 ori pe săptămână
- ☐ 4 O dată pe zi
- ☐ 5 De două ori pe zi
- ☐ 6 De trei sau mai multe ori pe zi

© 2014 Acest chestionar este protejat prin legea dreptului de autor 

6. Cât de des în ultima săptămână ați mâncat o porție de **sos de roșii** *(nu ketchup)*? ***(Includeți** sos de roșii pentru paste, cu pizza sau alte preparate)*.

- ☐ 1 Niciodată
- ☐ 2 O dată sau de două ori în ultima săptămână
- ☐ 3 3 – 6 ori pe săptămână
- ☐ 4 O dată pe zi
- ☐ 5 De două ori pe zi
- ☐ 6 De trei sau mai multe ori pe zi

© 2014 Acest chestionar este protejat prin legea dreptului de autor

*Următoarele întrebări sunt despre consumul dvs. de* ***alimente bogate în grăsimi****.*

***Alegeți <u>un singur</u> răspuns pentru fiecare categorie.***

| Cât de des mâncați ... ? | | Niciodată sau mai puțin de o dată pe lună | De 1-3 ori pe lună | De 1-2 ori pe săptămână | De 3-4 ori pe săptămână | De 5 ori sau mai mult pe săptămână |
|---|---|---|---|---|---|---|
| 7. | Carne de vită sau porc prăjit/ friptură | ☐ 1 | ☐ 2 | ☐ 3 | ☐ 4 | ☐ 5 |
| 8. | Carne de pui prăjit cu tot cu piele, pește prăjit | ☐ 1 | ☐ 2 | ☐ 3 | ☐ 4 | ☐ 5 |
| 9. | Hamburger, cheeseburger, carne tocată | ☐ 1 | ☐ 2 | ☐ 3 | ☐ 4 | ☐ 5 |
| 10. | Cârnați | ☐ 1 | ☐ 2 | ☐ 3 | ☐ 4 | ☐ 5 |
| 11. | Brânză/preparate de brânză *(nu cele degresate)* | ☐ 1 | ☐ 2 | ☐ 3 | ☐ 4 | ☐ 5 |
| 12. | Lapte integral, lapte semidegresat, frișcă, smântână | ☐ 1 | ☐ 2 | ☐ 3 | ☐ 4 | ☐ 5 |
| 13. | Margarină, unt | ☐ 1 | ☐ 2 | ☐ 3 | ☐ 4 | ☐ 5 |
| 14. | Sosuri pentru salate, maioneză | ☐ 1 | ☐ 2 | ☐ 3 | ☐ 4 | ☐ 5 |
| 15. | Cartofi prăjiți sau chipsuri | ☐ 1 | ☐ 2 | ☐ 3 | ☐ 4 | ☐ 5 |
| 16. | Grisine/sticksuri, floricele de porumb/ biscuiți | ☐ 1 | ☐ 2 | ☐ 3 | ☐ 4 | ☐ 5 |
| 17. | Ciocolată, bomboane, biscuiți | ☐ 1 | ☐ 2 | ☐ 3 | ☐ 4 | ☐ 5 |
| 18. | Înghețată, gogoși, produse de patiserie, plăcinte, prăjituri, biscuiți, covrigi | ☐ 1 | ☐ 2 | ☐ 3 | ☐ 4 | ☐ 5 |

© 2014 Acest chestionar este protejat prin legea dreptului de autor

# Îngrijiri preventive

*Următoarele întrebări se referă la servicii medicale de care dvs. probabil ați beneficiat.* ***Vă rugăm să răspundeți fiecărei întrebări cu „NU” sau „DA”***

| Ați avut... | | Nu | Da |
|---|---|---|---|
| 1. | O măsurare a tensiunii arteriale în ultimele 6 luni? | ☐ 1 | ☐ 2 |
| 2. | O determinare a glicemiei à jeun (dimineața pe nemâncate) în ultimele 6 luni? | ☐ 1 | ☐ 2 |
| 3. | O determinare a colesterolului în ultimele 6 luni? | ☐ 1 | ☐ 2 |
| 4. | Un consult oftalmologic în ultimele 6 luni? | ☐ 1 | ☐ 2 |
| 5. | O evaluare a auzului în ultimele 6 luni? | ☐ 1 | ☐ 2 |
| 6. | Vaccin antigripal în ultimele 6 luni? | ☐ 1 | ☐ 2 |

## Vă mulțumim pentru că ați completat acest chestionar!

© 2014 Geriatric Research Fund, Spital Netz Bern AG, Inselspital, PO Box 20, CH-3010 Bern, Switzerland. Acest chestionar este protejat prin legea dreptului de autor și nu poate fi (parțial sau total) reprodus sau utilizat în alte scopuri fără aprobarea prealabilă scrisă a deținătorilor dreptului de autor.

**Annex IX**

# Feedback Questionnaire

In this study we have evaluated the potential benefits of using the HRA-O questionnaire in clinical practice to guide counseling on preventive health measures and health education and promotion. Based on your responses to the HRA-O questions, your doctor has offered a counseling session tailored to your health and lifestyle needs in order to improve your general health status.

This brief survey asks questions about your experience being part of this study. Your responses will help us improve recommendations and practice.

Please do not complete this table:

| | |
|---|---|
| ID-number | |
| Location | ☐1 Hospital ☐2 Out-patient |
| First name | |
| Surname | |
| Date of examination | |

1. Do you believe the advices and information your doctor has given you during counseling sessions helped you take measures to improve your health and well being?

☐1 Yes, a lot → Please go to question no.3 on next page

☐2 Yes, somewhat → Please go to question no.3 on next page

☐3 No

© 2014 This questionnaire is protected by copyright.

2. Why do you feel the counseling sessions did NOT helped you improve your health and well being?

☐1 Because I find the recommendations too difficult to follow

☐2 Because I did not received any new information

☐3 Because I do not believe that my health will improve if I follow these recommendations

☐4 Because of financial reasons

☐5 Other reasons (please specify)

______________________________________________________________________
______________________________________________________________________
______________________________________________________________________

3. Do you believe that your scheduled visits to the doctor have become more regular as a result of the advices and information your doctor has given you during counseling sessions?

☐1 Yes, a lot

☐2 Yes, somewhat

☐3 No

Please take a moment to look back and make sure you have not missed any pages or questions.

**Thank you for completing this survey!**

© 2014 This questionnaire is protected by copyright.

**Annex X**

**Intervention documentation form:**

**Reason why one or more intervention elements did not take place**

| | |
|---|---|
| ID-number | |
| First name | |
| Surname | |
| Location | ☐1 Hospital ☐2 Out patient |

| Element | Date conducted | Duration | If not conducted, Reason why not conducted<br>Death<br>Nursing home<br>In Hospital<br>Refusal<br>Other |
|---|---|---|---|
| Baseline | | | |
| 1-month | | | |
| 2-month | | | |
| 3-month | | | |
| 4-month | | | |
| 5-month | | | |
| 6-month | | | |

## Health domains where patient received written recommendations

| Intervention | Health Domain | | | | | | | | |
|---|---|---|---|---|---|---|---|---|---|
| | M/P | PSHW | SN | TU | AU | PA | N | FP | PC |
| Baseline | | | | | | | | | |
| 1-month | | | | | | | | | |
| 2-month | | | | | | | | | |
| 3-month | | | | | | | | | |
| 4-month | | | | | | | | | |
| 5-month | | | | | | | | | |
| 6-month | | | | | | | | | |

M/P = Medications/Prescriptions
PSHW = Psychosocial Health & Well Being
SN = Social Network
TU = Tobacco Use
AU = Alcohol Use
PA = Physical Activity
N = Nutrition
FP = Falls Prevention
PC = Preventive Care
