# Supplementary material for: Effects of health risk assessment and counselling on physical activity in older people: A pragmatic randomised trial
Source: PLoS One. 2017 Jul 20;12(7):e0181371. doi: 10.1371/journal.pone.0181371 (PMC5519086; doi:10.1371/journal.pone.0181371)
Supplement: S3 Text — (PDF) [file pone.0181371.s006.pdf]

Detailed Analysis Plan Romania: RAHEO: Study on Physical Activity

1. Comparison Baseline Characteristics Intervention Versus Control Group

1. A. General Table

Table with p-values, no effect measures, for the following variables.

Variable Name	Description for Table	Technical Comment
**AGE**	Age	
**PE0100**	Gender	
**A6INCOMEGEQ848**	Income	>847 (above average pension)
**AY0500DICH**	Level of education	Categories high school or more
**AY0300DICH**	Living alone	Category living alone
	Number of chronic conditions	Number of chronic conditions based on positive answers page 3 of baseline questionnaire part 1 (19 items)
**HM0100DICH**	Fair or poor self-perceived health	Categories fair or poor (first question page 2 (baseline questionnaire, part 1)
	Presence of moderate to severe pain	Severity of pain >=3 based on items 1 or 2 page 4 (baseline questionnaire, part 2)
**IADLCATHELP**	Need for help in IADL	Need for help in IADL (category 3 in one or more of the 5 IADL items (No. 7 to 11) page 11 of the baseline questionnaire part 2))

1. B. PA specific Table

Variable<br>Name	Description for Table
**IPAQMET (main variable)**	MET minutes per week
**IPAQMET ≥ 450**	Physical activity of ≥ 450 MET minutes per week
**IPAQMET ≥ 900**	Physical activity of ≥ 900 MET minutes per week
**VIGACTCAT**	Vigorous physical activity ≥ once per week
**MODACTCAT**	Moderately vigorous or vigorous physical activity ≥1 once per week
**WALKACTMINWEEK**	Minutes of walking per week
**PA0700**	Sitting ≥4 hours per day during last week
**PA0800**	Intention to increase PA (based on answer to item 8, page 18, baseline questionnaire, part 2)
**PA0900**	Reasons for not increasing PA among those with no intention to increase PA (based on answer to item 9, page 18, baseline questionnaire, part 2)

2. Primary and Secondary Outcomes

2.A. Primary Outcome

We hypothesize that the intervention results in an increased level of physical activity of the older persons. Specifically, we hypothesize that persons in the intervention group have a higher number of MET minutes as compared to persons in the control group.

**Technical Name or Primary Outcome Variable**	**Description for Table**
**IPAQMET (main variable)**	MET minutes per week

2.B. Secondary outcomes: Alternate definitions of primary outcome:

A higher proportion of persons in the intervention group has a high level of physical activity compared to persons in the control group (more than 450 MET minutes per week/ more than 900 MET minutes per week)

**Technical Name of Secondary Outcome Variable**	**Description for Table**
**IPAQMET ≥ 450**	PA of ≥ 450 MET minutes per week
**IPAQMET ≥ 900**	PA of ≥ 900 MET minutes per week

2.C. Secondary Outcomes: Types of Physical Activity

The intervention has favourable effects on different types of physical activity, as measured with the IPAQ:

- Persons in the intervention group engage more often in vigorous physical activity as compared to persons in the control group
- Persons in the intervention group engage more often in moderately vigorous physical activity as compared to persons in the control group
- Persons in the intervention group engage more often in walking activity as compared to persons in the control group
- Persons in the intervention group spend less time sitting as compared to persons in the control group

Technical Name of Secondary Outcome Variable	Description for Table
**VIGACTCAT**	Vigorous PA ≥1/week
**MODACTCAT**	Moderately vigorous or vigorous PA ≥1/week
**WALKACTMINWEEK**	Minutes of walking per week
**PA0700**	Sitting ≥4 hours per day during last week

2.D. Pre-Post Analysis

We will conduct additional analyses for the primary variable to check whether there is a statistically significant difference between baseline and follow-up in intervention group, and in control group.

3. Sensitivity Analyses

Possible selection bias will be assessed by repeating analyses of the primary care outcome with an inverse probability of censoring weighting (IPCW) approach.

4. A priori Subgroup Analyses

- If overall favourable effects, then the question is: Does the intervention have favourable effects in the following subgroups as well?
- If overall no favourable effects, then the question is: Does the intervention have favourable effects in certain subgroups, but not in the entire group?

The question is not, whether effects differ between subgroups (sample size limitation).

The following subgroups should be analysed:

Subgroup category: Variable name	Subgroup Category: Descriptor	Definition of Subgroups
**PE0100**	Gender	Men vs women
**A6income**	Income	<847 vs >847 RON (percent below vs. above average pension)
**PA0800**	Intention to change PA	1 vs 2/3 (no intention vs with intention)
**HM0100-DICH**	Self-perceived health	3/4 vs 1/2 (fair poor vs good)

No plan for using statistical tests (P-values or effect measures), descriptive results.

5. Statistical Methods

Group differences will be tested by a chi-squared test for binary variables, and by a Kruskal-Wallis test for continuous variables. For binary variables we will report odds ratios (OR) with 95% confidence intervals (CI) from logistic regression models as effect measure. For continuous variables we will report median group differences (MD) from quantile regression models [Koenker R. and Bassett G.: Regression Quantiles; Econometrica; Vol. 46, No. 1 (Jan., 1978), pp. 33-50] with 95% CI. All p-values are two-sided.

Possible selection bias will be assessed by an inverse probability of censoring weighting (IPCW) approach [Robins JM, Hernán MA, Brumback B. Marginal structural models and causal inference in epidemiology. Epidemiology 2000;11:550-560; Weuve J, Tchetgen Tchetgen EJ, Glymour MM, Beck TL, Aggarwal NT, Wilson RS, Evans DA, Mendes de Leon CF. Accounting for bias due to selective attrition: the example of smoking and cognitive decline. Epidemiology 2012;23:119-128]. Statistical analyses will be performed in R V.3.1.1 (R Project, University of Vienna, Austria).
