# Supplementary material for: Effects of health risk assessment and counselling on physical activity in older people: A pragmatic randomised trial
Source: PLoS One. 2017 Jul 20;12(7):e0181371. doi: 10.1371/journal.pone.0181371 (PMC5519086; doi:10.1371/journal.pone.0181371)
Supplement: S4 Text — (PDF) [file pone.0181371.s007.pdf]

Ministerul Mediului, Apelor şi Pădurilor
ADMINISTRAȚIA NAȚIONALĂ DE METEOROLOGIE
METEO ROMANIA

Nr. Înreg.: 536/09.02.2016

Către: Doamna Doctor Anna Marie HERGHELEGIU

Referitor la: Număr lunar de zile cu precipitații atmosferice ≥0.1mm, temperatura maximă a aerului ≥30°C și temperatura minimă a aerului ≤0°C.

Stimată doamnă doctor Anna Marie Herghelegiu,

Urmare a solicitării dumneavoastră, înregistrată la Administrația Națională de Meteorologie cu nr. 536/09.02.2016, vă furnizăm date lunare privind numărul total lunar de zile cu precipitații atmosferice ≥0.1mm înregistrate în perioadele mai-iulie 2014, noiembrie-decembrie 2014 și ianuarie-februarie 2015; temperatura maximă a aerului ≥30°C înregistrată în perioada mai-iulie 2014 și temperatura minimă a aerului ≤0°C înregistrată în perioadele noiembrie-decembrie 2014 și ianuarie-februarie 2015 la stația meteorologică București Băneasa.

Datele meteorologice se transmit în format fizic și electronic conform solicitării dumneavoastră.

Director Executiv,

Dr. Elena MATEESCU

Șef Secție Climatologie,

Dr. Roxana BOJARIU

Întocmit,

Ciurlău Doruța

Şos. Bucureşti - Ploieşti nr. 97, sector 1, 013686, Bucureşti, România
Telefon: 021 316 31 16 / Fax: 021 316 31 43
e-mail:relatii@meteoromania.ro / www.meteoromania.ro

Date meteorologice înregistrate la stația meteorologică București Băneasa

Anul	Luna	Numărul lunar de zile cu precipitații atmosferice ≥ 0.1mm	Numărul lunar de zile cu temperatura maximă a aerului ≥ 30°C	Numărul lunar de zile cu temperatura minimă a aerului ≤ 0°C
2014	Mai	10	0	
	Iunie	19	2	
	Iulie	16	12	
	Noiembrie	12		10
	Decembrie	16		22
2015	Ianuarie	13		23
	Februarie	12		20

Responsabil de contract:
Ciurlău Doruța
Ciurlău D.
